# Lifetime effects and cost-effectiveness of standard and higher-intensity statin therapy across population categories in the UK: a microsimulation modelling study

**DOI:** 10.1016/j.lanepe.2024.100887

**Published:** 2024-03-22

**Authors:** Borislava Mihaylova, Runguo Wu, Junwen Zhou, Claire Williams, Iryna Schlackow, Jonathan Emberson, Christina Reith, Anthony Keech, John Robson, Richard Parnell, Jane Armitage, Alastair Gray, John Simes, Colin Baigent

**Affiliations:** a Health Economics Research Centre, Nuffield Department of Population Health, University of Oxford, Oxford, UK; b Health Economics and Policy Research Unit, Wolfson Institute of Population Health, Queen Mary University of London, London, UK; c Clinical Trial Service Unit and Epidemiological Studies Unit, Nuffield Department of Population Health, University of Oxford, Oxford, UK; d MRC Population Health Research Unit, University of Oxford, UK; e NHMRC Clinical Trials Centre, University of Sydney, Sydney, Australia; f Clinical Effectiveness Group, Wolfson Institute of Population Health, Queen Mary University of London, London, UK; g Patient and Public Representative, UK

**Keywords:** Cardiovascular diseases, Statin, Cost-effectiveness, Quality-adjusted life years, Health care costs, Microsimulation model

## Abstract

**Background:**

Cardiovascular disease incidence and mortality have declined across developed economies and granular up-to-date cost-effectiveness evidence is required for treatments targeting large populations. To assess the health benefits and cost-effectiveness of standard and higher intensity statin therapy in the contemporary UK population 40–70 years old.

**Methods:**

A cardiovascular disease microsimulation model, developed using the Cholesterol Treatment Trialists' Collaboration data (117,896 participants; 5 years follow-up), and calibrated in the UK Biobank cohort (501,854 participants; 9 years follow-up), projected risks of myocardial infarction, stroke, coronary revascularization, diabetes, cancer and vascular and nonvascular death for all UK Biobank participants without and with statin treatment. Meta-analyses of trials and cohort studies informed statins’ relative effects on cardiovascular events, incident diabetes, myopathy and rhabdomyolysis. UK healthcare perspective was taken (2020/2021 UK£) with costs per 28 tablets of £1.10 for standard (35%–45% LDL cholesterol (LDL-C) reduction) and £1.68 for higher intensity (≥45% LDL-C reduction) generic statin.

**Findings:**

Across categories by sex, age, LDL-C, and cardiovascular disease history/10-year cardiovascular risk, lifetime standard statin increased survival by 0.28–1.85 years (0.20–1.09 quality-adjusted life years (QALYs)), and higher intensity statin by further 0.06–0.40 years (0.03–0.20 QALYs) per person. Standard statin was cost-effective across all categories with incremental cost per QALY from £280 to £8530, with higher intensity statin cost-effective at higher cardiovascular risks and higher LDL-C levels. Stopping statin early reduced benefits and was not cost-effective.

**Interpretation:**

Lifetime low-cost statin therapy is cost-effective across all 40–70 years old in UK. Strengthening and widening statin treatment could cost-effectively improve population health.

**Funding:**

UK 10.13039/501100000664NIHR Health Technology Assessment Programme (17/140/02).

**Research in context**

**Evidence before this study**

Cardiovascular disease remains a leading cause of morbidity and mortality worldwide. Statin therapy has been reliably shown to reduce LDL cholesterol (LDL-C) and the incidence of cardiovascular disease by about a fifth for every 1 mmol/L reduction in LDL-C across different patients irrespective of their age, sex, cardiovascular risk of LDL-C level, with more intensive regimens achieving larger cardiovascular risk reductions. Generically available, statins are now a cornerstone in cardiovascular disease prevention worldwide with guidelines recommending it for people with history of cardiovascular disease or at an increased cardiovascular risk, or with high LDL-C level. Given the broad relevance of statin treatment to the population it is pertinent to provide contemporary evidence for the value of statin therapy in categories of individuals to inform future guidelines. We last searched PubMed on 17th September 2023 using the following search terms ((cost-effective∗ OR cost-utility OR economic evaluation OR cost-benefit) AND (statin therapies OR hydroxymethylglutaryl-CoA reductase inhibitors [MeSH Terms])) and selected articles published in English. 227 publications were screened and 15 studies on cost-effectiveness of generic statin therapy versus control in population categories ≥40 years old in high-income settings were selected. Five studies in populations with cardiovascular disease history, reported standard statin therapy and high intensity statin therapy being cost-effective. Ten studies in populations without history of cardiovascular or coronary heart disease reported statin therapy being cost-effective in populations at increased cardiovascular disease risk and LDL-C level. However, previous studies often used now outdated population risks data, did not validate their disease models, did not include considerations of statin adverse events and did not present results in distinct population categories.

**Added value of this study**

This is the most detailed study to date to investigate the value of statin therapy using granular data from a contemporary population cohort. This novel study utilised detailed data from individuals and explicitly integrated individualised risk of incident diabetes and risks of myopathy and rhabdomyolysis and their dose–response associations with statin intensity. The study provides definitive, detailed, high-quality evidence that standard statin therapy cost-effectively reduces cardiovascular risk and enhances quality-of life-adjusted survival in all categories of people 40–70 years old independently of their age, sex, cardiovascular risk or LDL-C level with higher intensity statin therapy providing further benefits cost-effectively in categories at higher cardiovascular risk or with higher LDL-C level. It extends the evidence to categories of men and women with low to moderate cardiovascular risks and lower levels of LDL–C not currently considered for statin treatment. The study also underlines the importance of LDL-C level in modulating the benefit achieved with statin therapy.

**Implications of all the available evidence**

Many people are suffering preventable cardiovascular disease due to suboptimal use of effective low cost therapies such as statin. Our findings importantly update and extend previous evidence to distinct population categories and show that long-term statin therapy improves health, lengthens life and is good value for money for all categories of adults 40–70 years old in UK. By focussing on statins’ value in distinct categories, our study strengthens the public health message of value of statin therapy to individuals such as women and younger middle-aged people where suboptimal statin use perseveres and the added benefit with higher intensity statin therapy among people without previous cardiovascular disease but at higher cardiovascular risk or with higher LDL-C level. Our findings suggest that strengthening and widening statin use has the potential to cost-effectively improve population health.

## Introduction

Cardiovascular disease, primarily myocardial infarction or stroke, is a leading cause of morbidity and mortality worldwide.^1^ Hydroxymethylglutaryl–coenzyme A reductase inhibitors (statins) reduce low-density lipoprotein cholesterol (LDL-C), a major causal risk factor of atherosclerotic cardiovascular disease. Statins reduce the incidence of major vascular events, defined as non-fatal myocardial infarction or coronary death, any stroke, or coronary revascularisation procedure), by around a fifth for every 1 mmol/L reduction in LDL-C achieved across a wide range of patients irrespective of their age, sex, previous history of cardiovascular disease or diabetes, or cardiovascular risk.^2^^,^^3^ Indeed the proportional reduction in major vascular events was at least as big in the lowest risk category (i.e. patients without substantial risk factors at 5-year risk of major vascular event <5%) as in the higher risk categories.^3^ More intensive statin regimens achieve larger reductions in LDL-C and prevent more cardiovascular events.^2^ Widely available as generic formulations, statins are now a cornerstone in cardiovascular disease prevention worldwide.

The ESC [2021]^4^ and US AHA/ACC [2018]^5^ guidelines recommended that statin therapy is used by individuals with a history of cardiovascular disease and those without previous cardiovascular disease depending on pre-treatment cardiovascular risk and LDL-C level, comorbidities or risk modifiers. In England and Wales, the National Institute for Health and Care Excellence (NICE) recommends statins for all individuals with a history of previous cardiovascular disease and, since 2014, for those without such a history who have a 10-year cardiovascular risk ≥10%.^6^ However, while millions of such individuals in UK take statins, many do not, and persistent gaps in treatment continue to be reported with discontinuation rate of about 30% reported in primary^7^ and 10% in secondary^8^ cardiovascular disease prevention by the end of the first year of treatment. The recent NICE guideline update also noted that statin treatment should not be ruled out if the estimated 10-year cardiovascular risk was less than 10% if the patient had an informed preference for taking statin or their risk might be underestimated,^6^ opening the possibility for wider statin use but stopping short of concrete guidance.

Statins have been shown to be remarkably safe across high quality studies. However, rarely they can cause significant muscle damage (i.e. myopathy and rhabdomyolysis),^9^ and a small excess of mild muscle symptoms^10^ and excess of new onset diabetes diagnoses^11^ have been reported. The risks of adverse events appear greater with more intensive statin regimen.^12^ Statin intolerance has been reported in about 5–10% of statin therapy initiators.^13^

Over the last few decades developed economies have seen remarkable reductions in cardiovascular disease incidence and mortality.^1^ In the UK the rates of cardiovascular mortality in 2019 were half and rates of cardiovascular disease incidence three-quarters of the levels observed twenty years previously with reductions in risks likely due to a range of life-style and therapeutic factors. Declines in total and non-HDL cholesterol have been reported across high income countries, including UK, and largely attributed to dietary changes.^14^ These reductions in risks are expected to lead to declining absolute benefits obtained from statin therapy which may affect the balance between benefits and risks from therapy. Current cost-effectiveness studies are, however, limited by either use of outdated data on cardiovascular disease risks and mortality in their long-term cost-effectiveness models,^6^^,^^15^ focus on population-wide results with lack of detailed evidence of long-term effectiveness and cost-effectiveness to inform policy in distinct patient categories,^15^^,^^16^ or lack consideration of individuals’ risks of adverse effects such as diabetes on statin therapy.^17^ Evidence for long-term net effects and cost-effectiveness of statins of different intensity in different categories of individuals is lacking.

Therefore, we aimed to estimate the net health effects and cost-effectiveness of statin therapies of different intensity across a wide range of distinct population categories in the contemporary UK population, an analysis highly relevant to both policy-makers and people considering statin therapy.

## Methods

**Fig. 1:**
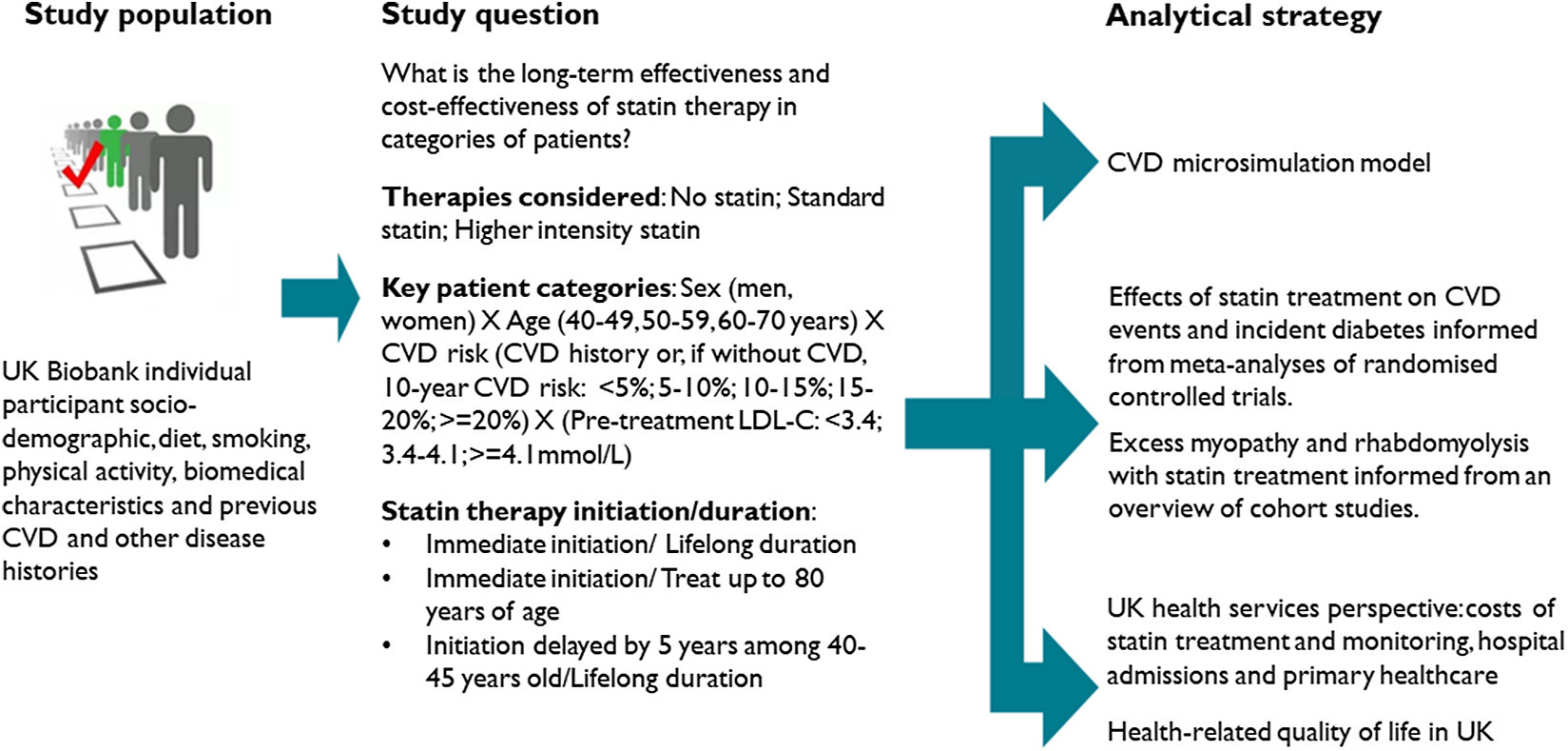
**Schematic of the evaluation**. UK, United Kingdom; LDL-C, low density lipoprotein cholesterol.

Our approach to the evaluation is summarised in Fig. 1.

### Study population

We assessed the lifetime effectiveness and cost-effectiveness of statin therapy in categories of UK adults 40–70 years old at start of treatment. To simulate the effects of statin therapies, we used the individual profiles of participants in the UK Biobank, which is a large prospective cohort of more than 500,000 UK volunteers aged 40–70 years at recruitment between 2006 and 2010, with detailed characteristics including demographic, behavioural, physical, and clinical characteristics and disease histories. Missing data was limited to socioeconomic characteristics (0.1%–2.2% across characteristics), blood pressure (0.3%), cholesterol (6.7% LDL-C, 14.5% HDL cholesterol) and creatinine (6.6%) and missing values were imputed. All UK Biobank participants, except a small number with end stage kidney disease, were included in the analyses. We summarise results across categories of participants by sex, age, LDL-C level without statin therapy (i.e. untreated LDL-C), history of previous cardiovascular disease, and for those without history of cardiovascular disease, by their estimated 10-year risk of cardiovascular disease, defined as a composite outcome of coronary heart disease, ischaemic stroke, or transient ischaemic attack, using the QRISK3 risk score. Further details on specification of participant characteristics, including imputation of missing data and estimation of QRISK3, are provided in the Supplementary methods.

### Effects and costs of statin therapy

Individual participant data meta-analysis of large randomised statin trials^18^ informed the relative reductions of the risks of cardiovascular events per 1 mmol/L LDL-C reduction with statin therapy in the decision-analytic model (Table 1). We assessed the effects of standard statin therapy, defined as a therapy that achieves about 35%–45% LDL-C reduction (e.g. atorvastatin 20 mg/day, rosuvastatin 5–10 mg/day or simvastatin 40–80 mg/day), and higher intensity statin therapy achieving ≥45% reduction (e.g. atorvastatin 40–80 mg/day, rosuvastatin 20–40 mg/day) (Supplementary Table S1). In model analysis, the absolute LDL-C reduction achieved with standard or higher intensity statin therapy was derived using the therapy's proportional LDL-C reduction and participant's untreated LDL-C level (with effects of any ongoing statin therapy removed). Meta-analyses of statin therapies informed excess new-onset diabetes with standard^11^ or higher intensity^12^ statin therapy. An overview of cohort studies informed excess rates of myopathy and rhabdomyolysis with statin therapy.^9^ Generic statin medication costs^20^ and costs of consultations^21^ and blood lipids tests^22^ for initiation and monitoring of statin prescribing in the National Health Service were included (Table 1). Further details about the treatment effects in the decision-analytic model are provided in the Supplementary methods.

**Table 1:** Statin treatment effects and statin treatment costs.

Item	Value	Source
**Effects of statin therapy on cardiovascular events per 1 mmol/L reduction in LDL-C**, Rate Ratio (RR) (95% confidence interval (CI))		Cholesterol Treatment Trialists' Collaboration individual participant data meta-analysis^18^
Myocardial infarction	RR 0.76 (0.73–0.79)	
Stroke	RR 0.84 (0.80–0.89)	
Coronary revascularisation	RR 0.75 (0.73–0.78)	
Cardiovascular death	RR 0.88 (0.85–0.91)	
**Adverse effects of statin therapy on:**		
Incident diabetes, Odds Ratio (OR) (95% CI)		
With standard statin therapy compared to no statin treatment	OR 1.09 (1.02, 1.17)	Meta-analyses of randomised controlled trials^11^
With higher intensity statin therapy compared to standard statin therapy	OR 1.12 (1.04–1.22)	Meta-analyses of randomised controlled trials^12^
Myopathy		
Excess per 100,000 treated with statin therapy (95% CI)	11 (4, 27)	Overview of cohort studies^9^
Occurrence of myopathy is associated with reduction in quality of life over 30 days recovery period. Statin treatment is stopped.	0.017 QALY reduction in year	Modelling study^15^
Rhabdomyolysis		
Excess rate per 100,000 treated with statin therapy (95% CI)	3.4 (1.6, 6.5)	Overview of cohort studies^9^
Case fatality	10%	Overview of cohort studies^9^
Reduction in quality of life	50% over 7.5 days hospital admission and by 20% for further 30 days recovery	Modelling study^15^
**LDL-C reductions with statin therapy:**		
With standard statin therapy (e.g. atorvastatin 20 mg/day, rosuvastatin 5–10 mg/day or simvastatin 40–80 mg/day)	37%–43%; 43% used in base-case	Meta-analysis of randomised controlled trials^19^
With higher intensity statin therapy (e.g. atorvastatin 40–80 mg/day, rosuvastatin 20–80 mg/day)	48%–58%; 55% used in base-case	Meta-analysis of randomised controlled trials^19^
**Statin therapy costs**		
Standard statin therapy (e.g. atorvastatin 20 mg/day, rosuvastatin 5–10 mg/day or simvastatin 40–80 mg/day)	£14.09–£19.57 per year; £14.35 used in base-case	NHS Drug tariff, December 2021^20^
Higher intensity statin therapy (e.g. atorvastatin 40–80 mg/day, rosuvastatin 20–40 mg/day)	£15.91–£27.91 per year; £21.91 used in base-case	NHS Drug tariff, December 2021^20^
**Statin initiation and monitoring healthcare costs**		
In year of initiation (doctor and nurse consultations; tests of blood lipids, HbA1c, thyroid function)	£54.65	Unit costs for Health and Social Care^21^ NHS reference costs^22^
In subsequent years: a nurse consultation and a blood lipids test (for people with history of cardiovascular disease)	£12.05	Unit costs for Health and Social Care^21^ NHS reference costs^22^

CI, confidence interval; LDL-C, low density lipoprotein cholesterol; NICE, National Institute for Health and Care Excellence; NHS, National Health Service England; OR, odds ratio; QALY, quality adjusted life year.

### Cardiovascular disease microsimulation policy model

The publicly available cardiovascular disease microsimulation policy model has been reported elsewhere.^23^ Briefly, this decision-analytic model was developed using the individual participant data of 16 large statin versus control randomised clinical trials, and validated and calibrated using the individual participant data of the 500,000-large UK Biobank study. The model employs a broad range of patient characteristics (age, sex, ethnicity, physical activity, diet quality, quintile of socio-economic deprivation, body mass index (BMI), smoking status, blood pressure, serum lipid and creatinine levels, treated hypertension, and histories of previous cardiovascular disease, diabetes, cancer or mental illness) to annually project the first occurrence of four major cardiovascular events: myocardial infarction, stroke, coronary revascularisation, vascular death, and three key non-vascular events: incident diabetes, incident cancer and non-vascular death with the occurrence of any of these events impacting the risks of subsequent events. The model was validated across categories of participants in UK Biobank and Whitehall II cohorts, and against national mortality and cancer incidence rates and other published data. Participant characteristics and incident adverse events determine health-related quality of life^23^ and primary care and hospital admission costs^24^ in each year of the model. The cardiovascular disease microsimulation model was used to project event risks and survival and summarise life years, quality-adjusted life years (QALYs), and primary and hospital care costs over individuals’ remaining lifetimes (i.e. death or reaching 110 years of age) without and with statin therapy, and to assess the cost-effectiveness of different statin therapies in categories of individuals. Further details of the model, including model validation, are reported in the Supplementary methods.

### Cost-effectiveness of statin therapy

#### Base-case analysis

In our base-case analysis, we assessed the cost-effectiveness of lifetime statin therapy from the perspective of the UK National Health Service under a number of key assumptions based on current evidence. First, the reduction in individual's LDL-C level with a particular statin therapy was assumed to correspond to the average proportional reduction achieved with that therapy. Second, we assumed the relative effects of a particular statin therapy on event risks were independent of duration of therapy or individual person characteristics (e.g. the same in younger and older people and in men and women). Third, disease events were assumed not to differ in severity or otherwise, irrespective of statin treatment status. Finally, statin therapy was assumed not to affect the risks of cancer or other non-vascular events,^2^ nor confer any discomfort or disutility beyond the adverse events specified above.

#### Assessment of uncertainty

We ran 500 microsimulations per individual for each set of parameters. We summarised the parameter uncertainty, including uncertainty in effects of statin therapy on vascular and nonvascular events, all event risk equations in the decision-analytic model, and quality of life and healthcare costs related to participant characteristics and events, using 500 and 1000 sets of parameter values in participant categories without and with previous cardiovascular disease, respectively. Values for treatment effects were sampled from lognormal distributions corresponding to the natural logarithm of the relative risk reductions with statin therapy (Table 1). A bootstrap approach, employing sampling with replacement from respective populations, was used to derive the parameter sets across (1) the event risk equations and healthcare cost equations from the UK Biobank study population, and (2) the quality of life equation from the Health Survey of England data, while respecting correlations between equations and parameters therein.

We report life years and QALYs gained, the additional statin and other healthcare costs (2020/2021 UK£) and the additional or incremental costs per QALY gained with standard and higher intensity statin therapy. We followed the NICE manual for health technology evaluations and discounted future life years, QALYs and costs at 3.5% per year in summary measures of cost-effectiveness.^25^ We report cost-effectiveness acceptability curves and probability of cost-effectiveness for willingness to pay of up to £40,000 per QALY. The impact of the increased risk of incident diabetes with statin therapy was also quantified.

#### Sensitivity and scenario analyses

The following parameters were varied in sensitivity analyses. Firstly, we assumed that the relative risk reductions of cardiovascular events were not constant but, as suggested by Mendelian randomisation studies, increased annually. Therefore, following the first five years of treatment, we applied a further 1.5% proportional reduction of cardiovascular events per 1 mmol/L LDL-C reduction with statin therapy each year. A scenario analysis with declining relative reductions of cardiovascular events with duration of statin therapy was also added. Secondly, in view of higher uncertainty in the effects of statin therapy in older people, we applied relative risk reductions in cardiovascular risks above age 75 years, informed from data only among people older than 75 years of age in the individual participant data meta-analysis.^18^ Thirdly, we studied the impact of lower than expected LDL-C reduction with statin therapy. Fourthly, to assess sensitivity to variation in incident cancer risk, we ran scenario analyses with small detrimental or beneficial statin effect on incident cancer, informed by the 95% confidence interval limits reported in an individual participant data meta-analysis of randomised statin trials. Fifthly, to assess sensitivity to potentially higher mortality risk among categories at greater socioeconomic deprivation, a scenario analysis with 20% higher risk of nonvascular death is included. Sixthly, in acknowledgement of substantial rates of statin discontinuation and re-initiation over time, a scenario analysis assessed statin cost-effectiveness under real-world compliance with statin therapy over time, derived from routine UK data, with statin effects and costs discontinued with therapy discontinuation. Seventhly, to acknowledge the uncertainty concerning any quality of life disutility from taking a daily statin pill, we included analyses with disutility equal to 0.001, 0.002 or 0.003 each year.

In further sensitivity analyses, we varied effects of cardiovascular events or diabetes adverse effects on quality of life by decreasing them by 50% and used discount rates of 1.5% instead of 3.5% per year. We also present results of a scenario analysis where only healthcare costs for cardiovascular disease and incident diabetes are retained and a sensitivity analysis with higher costs of statin therapy (as generic drug prices may vary over time and vary internationally^26^). In a further scenario analysis we relaxed the assumption that individuals achieve the average proportional reduction by introducing variability around the proportional reduction in LDL-C with statin therapy. As part of the microsimulations for individuals, we sample the % reduction in LDL-C from normal distributions with mean 43% (standard deviation 14.5%) for standard statin and mean 55% (standard deviation 17.3%) for higher intensity statin therapy. Further details on the specification of sensitivity analyses are provided in the Supplementary methods.

Two scenario analyses addressed the question of when to initiate and how long to treat with statin therapy. We report cost-effectiveness of delaying statin therapy by 5 years in people without previous cardiovascular disease who were 40–45 years old. We also report cost-effectiveness of stopping statin therapy (and its treatment effects and costs) at 80 years of age instead of continuing treatment until death.

### Patient and public involvement

Members of the public were involved from the outset of this research. Two lay persons were members of the study management team, and an independent lay person was a member of the study steering group. In addition to these groups’ meetings, we held three separate patient and public involvement (PPI) sessions with our lay members to discuss our methods and results. Results from the research were also presented to and discussed with a wider group of members of the Nuffield Department of Population Health Public Advisory Panel, University of Oxford. PPI members helped us refine our methodology, our communication of individual versus population effects and our approach to presenting our findings to the public.

### Role of the funding source

This study was funded by the UK NIHR Health Technology Assessment (HTA) Programme (17/140/02). Further support from the British Heart Foundation (PG/18/16/33570 and CH/1996001/9454), the UK Medical Research Council (MC_UU_00017/4), the National Institute for Health Research Barts Biomedical Research Centre (NIHR203330) and NHMRC, Australia is acknowledged. The study funders had no role in the study design, analyses, interpretation of data, writing the manuscript, approval, or decision to publish the results. The views expressed are those of the author(s) and not necessarily those of the NIHR, the Department of Health and Social Care or any other funder.

## Results

### Baseline characteristics

The baseline characteristics of the 501,854 UK Biobank study participants are presented in Table 2 in categories by previous history of cardiovascular disease and, among those without cardiovascular disease history, by 10-year cardiovascular risk. Women, younger participants, participants with lower socio-economic deprivation and with healthier behaviours and positive risk factors, including lower LDL-C and higher HDL cholesterol levels, clustered into the lower risk categories. The higher cardiovascular risk categories contained more people on statin therapy, with treated hypertension or with diabetes. There were no 60–70 year old men with estimated 10-year cardiovascular risk<5%, and the small numbers of 40–49 year old men and women with 10-year cardiovascular risk >15% were combined with participants with 10-year cardiovascular risk of 10–15% in categories by LDL-C in summarising results (Supplementary Table S2).

**Table 2:** Baseline characteristics of UK Biobank participants.

Number of participants	Participants without cardiovascular disease history, by 10-year cardiovascular risk (QRISK3)										Participants with history of cardiovascular disease	
	<5%		5%–10%		10%–15%		15%–20%		≥20%			
	140,304		112,301		75,451		47,905		68,615		57,278	
Age, years	48.2	(5.4)	56.1	(6.1)	60.1	(5.7)	62.0	(5.3)	63.2	(5.1)	60.4	(7.0)
40–49	86,615	(61.7%)	18,423	(16.4%)	4034	(5.3%)	1463	(3.1%)	1596	(2.3%)	5612	(9.8%)
50–59	50,295	(35.8%)	54,445	(48.5%)	25,144	(33.3%)	11,098	(23.2%)	11,155	(16.3%)	14,783	(25.9%)
60–70	3394	(2.4%)	39,433	(35.1%)	46,273	(61.3%)	35,344	(73.8%)	55,864	(81.4%)	36,883	(64.5%)
Male sex	33,515	(23.9%)	41,969	(37.4%)	36,863	(48.9%)	30,336	(63.3%)	52,313	(76.2%)	33,734	(59.0%)
Ethnicity												
White	129,347	(92.2%)	106,869	(95.2%)	72,622	(96.3%)	46,197	(96.4%)	65,374	(95.3%)	54,488	(95.0%)
Black	4033	(2.9%)	1605	(1.4%)	753	(1.0%)	375	(0.8%)	502	(0.7%)	770	(1.3%)
South Asian	1866	(1.3%)	1480	(1.3%)	981	(1.3%)	719	(1.5%)	1900	(2.8%)	1053	(1.8%)
Other^a^	5058	(3.6%)	2347	(2.1%)	1095	(1.5%)	614	(1.3%)	839	(1.2%)	967	(1.7%)
Townsend socioeconomic deprivation												
Quintile 1 (least deprived)	56,420	(40.2%)	43,328	(38.6%)	28,189	(37.4%)	16,942	(35.4%)	21,262	(31.0%)	18,960	(33.2%)
Quintile 2	28,600	(20.4%)	22,762	(20.3%)	15,275	(20.2%)	9706	(20.3%)	13,054	(19.0%)	10,957	(19.2%)
Quintile 3	23,166	(16.5%)	18,333	(16.3%)	12,237	(16.2%)	7790	(16.3%)	11,100	(16.2%)	9034	(15.8%)
Quintile 4	18,964	(13.5%)	15,752	(14.0%)	11,019	(14.6%)	7285	(15.2%)	11,428	(16.7%)	9178	(16.1%)
Quintile 5	13,154	(9.4%)	12,126	(10.8%)	8731	(11.6%)	6182	(12.9%)	11,771	(17.2%)	9149	(16.0%)
Smoking												
Never	96,434	(68.7%)	66,546	(59.3%)	40,168	(53.2%)	22,386	(46.7%)	24,727	(36.0%)	25,137	(44.0%)
Former smoker	35,095	(25.0%)	35,165	(31.3%)	27,422	(36.3%)	19,417	(40.5%)	31,213	(45.5%)	25,211	(44.1%)
Current smoker	8775	(6.3%)	10,590	(9.4%)	7861	(10.4%)	6102	(12.7%)	12,675	(18.5%)	6930	(12.1%)
Physical activity												
High	47,746	(34.0%)	36,671	(32.7%)	24,263	(32.2%)	15,559	(32.5%)	20,967	(30.6%)	16,780	(29.3%)
Moderate	47,303	(33.7%)	36,783	(32.8%)	24,489	(32.5%)	15,438	(32.2%)	22,143	(32.3%)	17,679	(30.9%)
Low	20,788	(14.8%)	16,385	(14.6%)	10,848	(14.4%)	6860	(14.3%)	11,051	(16.1%)	10,105	(17.7%)
Missing	24,467	(17.4%)	22,462	(20.0%)	15,851	(21.0%)	10,048	(21.0%)	14,454	(21.1%)	12,714	(22.2%)
Unhealthy diet (incl. uncertain)	47,749	(34.0%)	37,622	(33.5%)	26,296	(34.9%)	17,962	(37.5%)	28,940	(42.2%)	21,705	(37.9%)
Body mass index (kg/m^2^)	26	(4.4)	27.1	(4.6)	27.7	(4.6)	28.1	(4.6)	29.2	(4.9)	29	(5.2)
On statin treatment	1015	(0.7%)	4935	(4.4%)	8159	(10.8%)	9667	(20.2%)	31,392	(45.8%)	31,325	(54.8%)
LDL-C (mmol/L), untreated^b^	3.4	(0.7)	3.8	(0.8)	4	(0.8)	4	(0.9)	4.3	(1.1)	4.1	(1.1)
<3.4	68,974	(49.2%)	32,142	(28.6%)	18,456	(24.5%)	10,961	(22.9%)	13,037	(19.0%)	15,674	(27.4%)
3.4–4.1	47,751	(34.0%)	43,253	(38.5%)	27,946	(37.0%)	16,706	(34.9%)	20,304	(29.6%)	17,011	(29.8%)
≥4.1	23,579	(16.8%)	36,906	(32.9%)	29,049	(38.5%)	20,238	(42.2%)	35,274	(51.4%)	24,593	(43.0%)
HDL-C (mmol/L)	1.6	(0.4)	1.5	(0.4)	1.5	(0.4)	1.4	(0.3)	1.3	(0.3)	1.3	(0.4)
Creatinine (μmol/L)	67.6	(12.3)	70.1	(13.5)	72.3	(14.2)	75	(15.0)	77.9	(18.4)	76.9	(19.3)
Systolic blood pressure (mmHg)	126.4	(14.4)	136.3	(15.9)	142.4	(16.7)	146.7	(17.3)	151.8	(18.6)	138.9	(18.8)
Diastolic blood pressure (mmHg)	78.8	(9.4)	82.4	(9.7)	84	(9.7)	85.2	(9.7)	86.3	(10.1)	80.9	(10.4)
Treated hypertension	3200	(2.3%)	11,144	(9.9%)	14,233	(18.9%)	13,104	(27.4%)	30,249	(44.1%)	26,184	(45.8%)
Prior diabetes	250	(0.2%)	975	(0.9%)	1667	(2.2%)	2375	(5.0%)	16,300	(23.8%)	8171	(14.3%)
Prior cancer	7316	(5.2%)	8256	(7.4%)	6421	(8.5%)	4215	(8.8%)	6505	(9.5%)	5861	(10.3%)
Severe mental illness	10,528	(7.5%)	9117	(8.1%)	6179	(8.2%)	3817	(8.0%)	6446	(9.4%)	6324	(11.1%)
History of cardiovascular disease												
None	140,304	(100%)	112,301	(100%)	75,451	(100%)	47,905	(100%)	68,615	(100%)	0	(0%)
Myocardial infarction only											2071	(3.6%)
Peripheral arterial disease only											6806	(11.9%)
Other coronary heart disease^c^ only											28,973	(50.6%)
Stroke only											5137	(9.0%)
Two or more of myocardial infarction, peripheral arterial disease, other coronary heart disease or stroke											14,291	(25.0%)

Values are mean (standard deviation) or number (%) following imputation of missing data (see supplementary methods for further details). HDL-C, high density lipoprotein cholesterol; LDL-C, low density lipoprotein cholesterol.

a Other ethnicity includes Chinese, Mixed, White and Black Caribbean, White and Black African, White and Asian, Any other mixed background and other ethnic group.

b Adjusted for use of statin treatment at baseline by statin type and dose.

c Other coronary heart disease includes acute rheumatic fever, chronic rheumatic heart diseases, hypertensive heart disease, angina pectoris, other acute ischaemic heart disease, chronic ischaemic heart disease, pulmonary heart disease and other form of heart disease.

### Base-case results for statin cost-effectiveness

In participant categories defined by age, sex, history of cardiovascular disease and 10-year cardiovascular risk, standard statin therapy was projected to increase individual survival (undiscounted) by 0.38–1.76 life years (0.28–1.10 QALYs), and higher intensity statin therapy by further 0.08 to 0.38 life years (0.04–0.23 QALYs) (Supplementary Figure S1). Across these categories, standard statin therapy had incremental cost per QALY gained ranging from £1090 to £6390 and higher intensity statin therapy from £2890 to £25,220 per QALY gained. At £20,000 willingness to pay per QALY, higher intensity statin therapy had the highest probability of being cost-effective across most categories of men and women, whilst standard statin therapy had the highest probability of being cost-effective only in some categories of younger women at lower cardiovascular risk (Supplementary Figure S2).

**Fig. 2:**
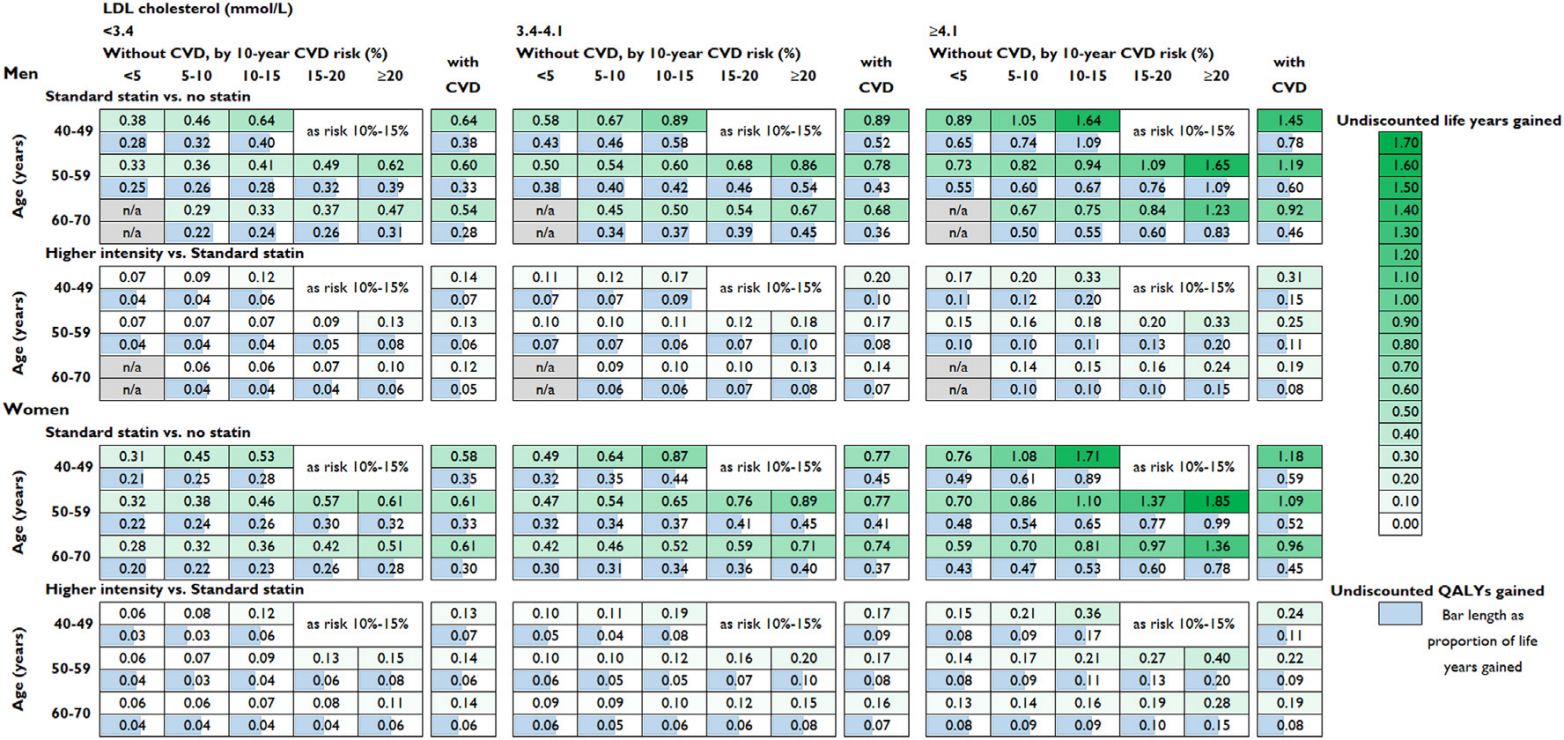
**Life years and QALYs gained per person with long-term statin therapy in categories by sex, age, pre-treatment LDL cholesterol level and cardiovascular risk**. QALY, quality-adjusted life year; LDL, low density lipoprotein; CVD, cardiovascular disease; n/a, not applicable (no UK Biobank participants in this category). Standard statin achieves 35%–45% LDL cholesterol reduction and higher intensity statin achieves ≥45% LDL cholesterol reduction.

**Fig. 3:**
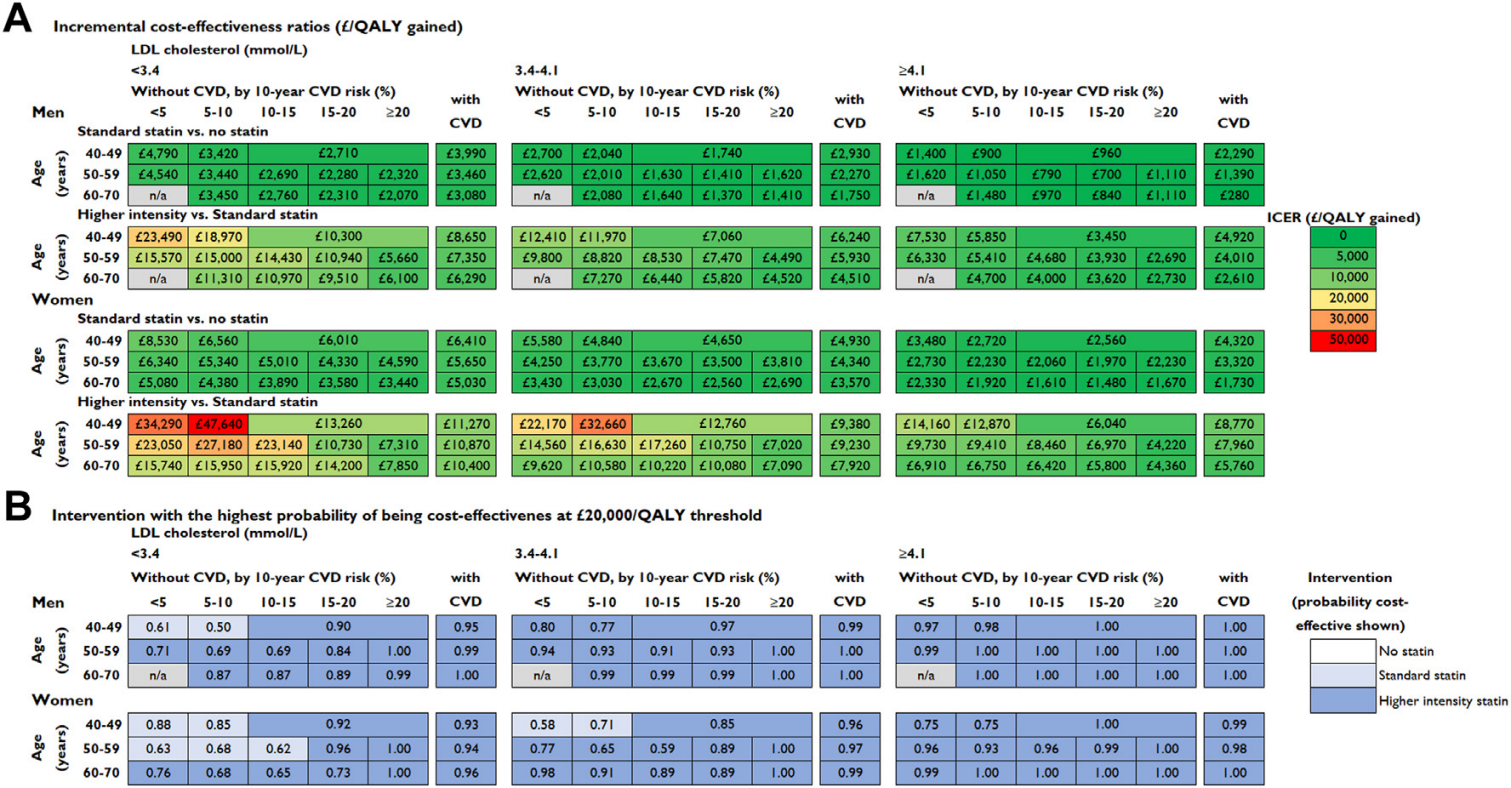
**Cost-effectiveness of long-term statin therapy in categories by sex, age, pre-treatment LDL cholesterol level and cardiovascular risk**. LDL, low density lipoprotein; CVD, cardiovascular disease; ICER, incremental cost-effectiveness ratio with costs and QALYs discounted at 3.5% per year. QALY, quality-adjusted life year; n/a, not applicable. Standard statin achieves 35%–45% LDL cholesterol reduction and higher intensity statin achieves ≥45% LDL cholesterol reduction.

We further stratified participant categories by LDL-C level prior to statin treatment. Across categories, lifetime use of standard statin therapy was projected to increase survival by 0.28–1.85 life years (0.20–1.09 QALYs), and higher intensity statin therapy by a further 0.06 to 0.40 life years (0.03–0.20 QALYs) per person, with larger gains among participants at higher cardiovascular risk and with higher LDL-C level at initiation (Fig. 2). Across population categories, standard statin therapy had incremental cost per QALY ranging from £280 to £8530/QALY with higher intensity statin therapy realising additional QALYs at an incremental cost per QALY gained ranging from £2610 to £47,640 (Fig. 3A, Supplementary Table S3). At a £20,000/QALY threshold, higher intensity statin therapy had the highest probability of being cost-effective in most participant categories and standard statin therapy was most likely cost-effective in categories with lower LDL-C and/or lower cardiovascular risk levels (Fig. 3B). The probability of standard or higher intensity statin therapy being cost-effective was high across all participant categories at a £10,000/QALY threshold and statin therapy remained highly likely cost-effective across most categories studied at a £5000/QALY threshold (Supplementary Figure S3). The excess risk of diabetes with standard statin therapy was evaluated to have reduced QALY gains by about 0.02–0.03 QALYs and with higher intensity statin therapy by further 0.03–0.05 QALYs (Supplementary Table S4).

### Sensitivity analyses

These cost-effectiveness results remained robust in a wide range of sensitivity analyses in most categories of participants studied (Supplementary Figures S4–S6 and Supplementary Table S5). However, the cost-effectiveness of standard statin therapy in younger individuals at low cardiovascular risk and with low LDL-C level was sensitive to the assumptions of declining relative risk reductions beyond year 5, an increase in cancer incidence with statin therapy, more than doubling of the cost of statin therapy, and quality of life disutility of a daily pill 0.002/year or above. For example, an added disutility of a daily pill of 0.002/year substantially reduced the benefits and cost-effectiveness of statin therapy for younger men and women with lower LDL-C and at low cardiovascular risk but did not materially affect statin cost-effectiveness in other participant categories (Supplementary Figure S7 and Supplementary Table S5). Results remained similar with the inclusion of variability around the proportional reduction in LDL-C (Supplementary Figure S6).

In scenario analyses, stopping statin therapy at 80 years of age substantially reduced QALY gains projected with long-term therapy and was not cost-effective (Supplementary Table S6). Delaying statin therapy by 5 years among 40–45 year olds, however, forgoes a small share of the QALYs gained compared with immediate initiation. Nevertheless, for all patient categories, except women at 10-year cardiovascular risk<5%, it remains cost-effective to initiate statin therapy immediately (Supplementary Table S7).

## Discussion

Our study re-evaluated the UK cost-effectiveness of statin therapy in categories of men and women 40–70 years old in present-day UK, by level of cardiovascular risk and pre-treatment LDL-C. We report that lifetime statin therapy increased quality of life adjusted survival in all patient categories studied, and, at the current UK cost of generic statin therapy and threshold for cost per QALY gained, was highly cost-effective across all individuals 40–70 years old in the contemporary UK society. Higher intensity statin therapy was cost-effective in many categories at higher cardiovascular risk or with higher LDL-C levels. Results remained robust in sensitivity analyses and at lower acceptable cost per QALY gained. Timely initiation and long-term persistence with statin therapy led to optimal benefits.

The study is a comprehensive and robust cost-effectiveness analysis of statin treatment for people 40–70 years old building on i) the best randomised evidence, ii) validated risk models in contemporary populations, and iii) well set-out assumptions when extrapolating beyond the randomised data. Unlike the cardiovascular disease decision-analytic modelling informing the current NICE guideline,^6^ the present study used recent individual participant data to model multivariable disease risks and integrated the adverse effects of statin therapy on diabetes incidence, myopathy and rhabdomyolysis. The study is unique also in reporting comprehensive results across many distinct population categories by age, sex, cardiovascular risk and LDL-C and for both standard and higher intensity statin regimens, directly informing decision-makers’ trade-offs, unlike previous analyses focusing on population-wide cost-effectiveness only^16^ or without consideration of statin intensity or individualised risks of adverse effects.^17^ Our findings for the value of higher intensity statin therapy extend previous findings in secondary prevention^27^ to higher cardiovascular risk and/or higher LDL-C primary prevention and report considerable value of higher intensity statin regimens in many primary prevention individuals at higher cardiovascular risk or with higher LDL-C levels. This detailed evidence will be highly informative not only for future guidelines but also in shared decision-making consultations between clinicians and patients where data about statins' effectiveness and adverse effects and patient's preferences are discussed.

Suboptimal statin adherence has been reported both in primary and secondary cardiovascular disease prevention.^7^^,^^8^ Muscle symptoms are often cited as a reason for statin discontinuation.^13^ However, only a small excess in muscle symptoms was reported with statin treatment across randomised studies with most of the perceived symptoms (>90%) likely not due to statin and any excess largely confined to the first year of treatment.^10^ Our study reports that, while statin discontinuation reduced health gains, it did not materially affect the cost-effectiveness of statin therapy as both health benefits and additional costs were similarly reduced. However, to optimise health benefits from statin treatment, its long-term use needs to improve. Re-challenging individuals to restart statin therapy has been shown effective but would require further effort and resources.

The study's cost-effectiveness findings remained robust across most sensitivity analyses though three areas of uncertainty should be noted. Firstly, the assumption of persistent relative reductions in cardiovascular events beyond the first 5 years exceeds the treatment duration in randomised trials. In sensitivity analysis, a substantial hypothetical decline in treatment effects over time reduced the value of statin treatment among younger people at lower cardiovascular risk and with lower LDL-C levels. Secondly, previous cost-effectiveness analyses have suggested that statin cost-effectiveness was sensitive to ‘disutility’ of taking a daily statin pill.^15^^,^^28^^,^^29^ Pill-taking disutility is difficult to quantify reliably with studies reporting high heterogeneity with the majority of patients experiencing zero disutility and a small proportion experiencing very high disutility (i.e. unlikely to persist with treatment).^30^ Due to the high level of heterogeneity our base-case analysis did not include pill-taking disutility. Our sensitivity analysis, however, indicated that large pill-taking disutility materially affects the value of statin therapy only among younger women at lower cardiovascular risk and with lower LDL-C levels. Thirdly, the proportional response to statin therapy vary across individuals.^31^ This variability did not materially affect the results we report across categories of patients. Nevertheless, if novel factors determining response to statin therapy emerge, these need to be included into future consideration of the value of statin therapy across populations.

**Fig. 4:**
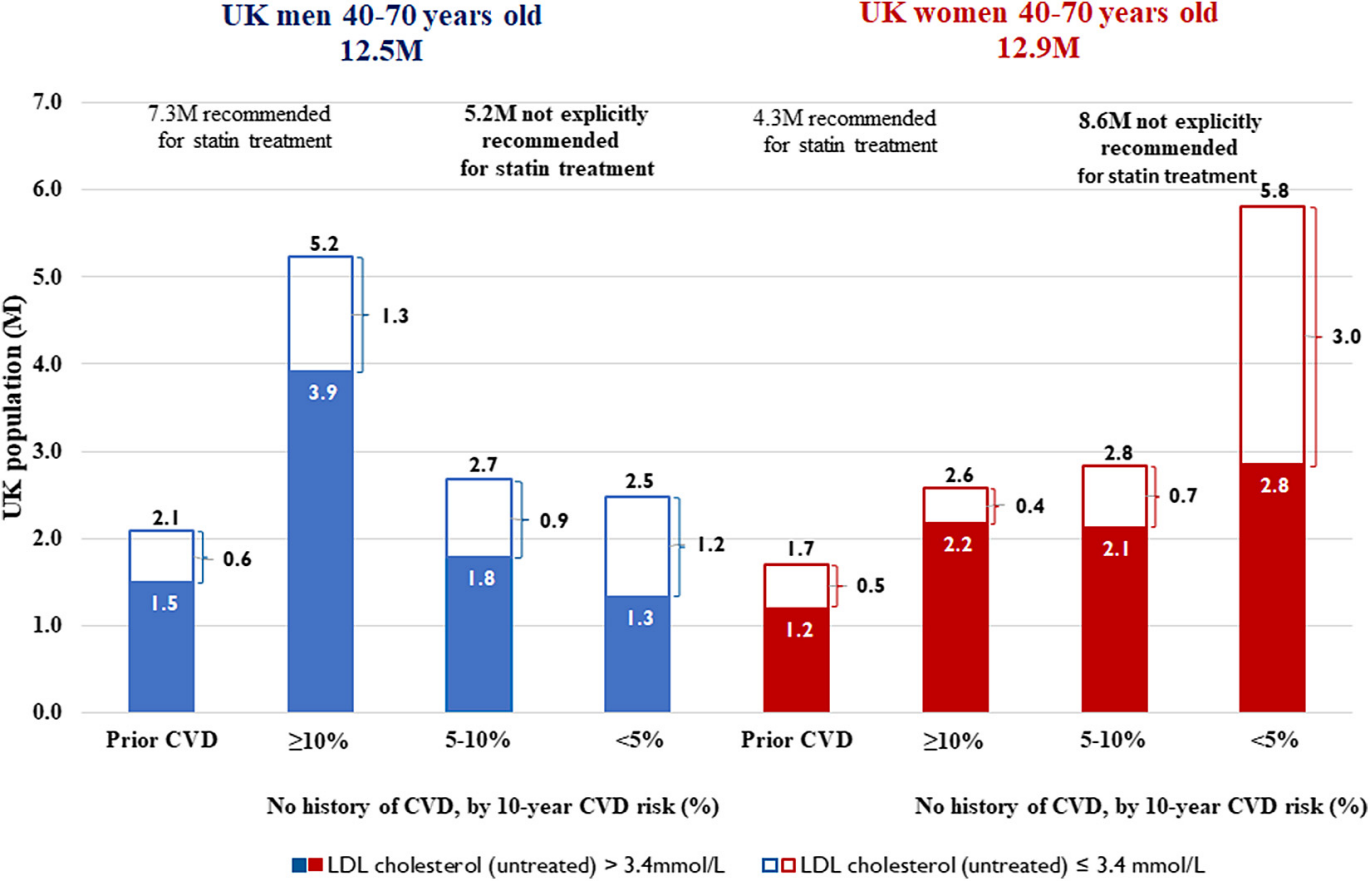
**UK population 40–70 years old, bycardiovascularrisk and untreated LDL cholesterol level**. Mid 2020 population estimates (ONS, 2021). Proportion of people with history of cardiovascular disease (CVD) sourced from Health Survey for England 2017. Distribution of people by 10-year CVD risk (QRISK3 <5%, 5–10%, ≥10%) and by LDL cholesterol level (≥3.4 mmol/L), by age and sex, based on UK Biobank data using participant characteristics at entry with untreated LDL cholesterol levels adjusted for reported statin use. In 2017, according Health Survey for England data, among people 40–74 years old about 50% of those with history of CVD and 15% of those without history of CVD were using statin therapy.

Our findings are relevant to the entire UK population 40–70 years old and have important implications for current policy. While about 46% of the 25.4 million UK adults 40–70 years old at mid-2020 population estimate are already recommended for statin therapy, the remaining 54% or 13.8 million, many of whom have raised LDL-C levels and among whom about 30% of cardiovascular events occur,^32^ are mostly not (Fig. 4). The recent update of the NICE guideline opens the possibility for statin treatment in this population.^6^ Our study, building upon the randomised evidence for reductions in cardiovascular events among people at low cardiovascular risks,^3^ suggests that, unless statin intolerant, they are likely to benefit from statin therapy cost-effectively, with those with high LDL-C levels likely to derive benefits comparable or exceeding benefits in patient categories currently treated. However, our findings pose a dilemma for policy makers faced with constrained resources. It is unclear whether the UK primary care has the resources to expand statin prescribing services at the same per person cost of initiating and monitoring treatment. Although statin treatment would remain cost-effective at somewhat higher costs, it is likely that substantial improvements and extensions to statin treatment would require prioritization and inclusion of broader patient and health service considerations.

The study findings, although informed from UK data, are likely highly relevant to other high-income country settings and the ranking of patient categories by additional cost per QALY could inform statin treatment priorities in middle and lower income country settings. Despite similar cardiovascular risk reductions and similar international prices, statin uptake is much lower than antihypertensive and diabetes treatment in low and middle income countries. Statin use in high-risk primary (10-year cardiovascular risk≥20%) and secondary prevention populations was reported to be 2% and 8.2% in low income countries, 6.3% and 21.2% in lower middle income countries, and 13.8% and 31.6% in upper middle income countries,^33^ well under the 50% target of the World Health Organisation and with clearly lower use in high-risk people without previous cardiovascular disease. The lack of comprehensive cost-effectiveness assessments in low and middle-income countries is one of the reasons for the underuse of effective low-cost statin. Our findings in the UK context indicate that the absolute benefits and cost-effectiveness of statin therapy for people without history of cardiovascular disease but with higher cardiovascular risk and with higher LDL-C level are similar to those in secondary prevention.

The key strengths of our study include use of high quality evidence and robust methodology. Effects of statin therapies were informed from individual participant meta-analysis of all large statin trials that have reported similar relative risk reductions per 1 mmol/L LDL-C reduction with statin therapy in participant categories by age, sex, history of cardiovascular disease or other risk factors and over different years of follow-up in the trials.^2^^,^^3^ Furthermore, we used the percentage reduction in LDL-C with statin regimens of different intensity together with individuals’ pre-treatment LDL-C level to derive the expected reductions in LDL-C individuals achieve and to further inform the expected relative risk reductions for individuals. The cardiovascular disease microsimulation model was derived and validated using large and rich individual participant data with substantial duration of follow up, enabling us to reliably model disease risks, quality of life adjusted survival and healthcare costs of individuals over time using their characteristics at entry. The model integrated the underlying risk of incident diabetes, which enabled us to project consequences of excess incident diabetes with statin therapy and quantify the trade-offs between cardiovascular risk reductions and excess diabetes risks with statin therapy of different intensity for different categories of individuals. It also integrated the risk of incident cancer, the largest competing risk affecting health-related quality of life and survival of individuals in parallel with any benefits from cardiovascular risk reductions. Ability to report results in distinct categories of actual person profiles and assess parameter uncertainty is a further strength.

The study limitations are rooted primarily in the limited current evidence about statin effects in older people and lack of randomised data on long-term effects of statin therapy. We used a number of assumptions informed from available evidence but found limited sensitivity of our findings to plausible alternative assumptions in sensitivity analyses. The analytical framework is based on projected risks for people 40–70 years old, and therefore, we do not report results of initiating treatment in younger and older individuals. The study reports results using the UK Biobank participants’ profiles, a healthier cohort than the general UK population. Nevertheless, we present results across a wide range of participant categories, which are likely to generalize well to similar categories in the general population. Furthermore, cost-effectiveness results for statin therapy did not differ materially in participant categories by quintile of socio-economic deprivation or lifestyle defined by diet quality and level of physical activity (results not shown). Though lifestyle modification and appropriate attention to other risk factors should accompany statin use,^4^, ^5^, ^6^ these are not likely to materially affect the value of statin therapy.

Future research should strengthen the evidence of effects of statin therapy in older people without history of cardiovascular disease. It has been hypothesised that many people younger than 40 years are also likely to benefit; data on the effects of statins in younger people and their commitment to take statin long-term, beyond the category of familial hypercholesterolemia, are however limited. In addition to strengthening evidence for effects of treatment, decision-analytic models, informed by data in older or younger people, and shown suitable for projecting disease risks in these population categories, will also be informative. Finally, disease risks in populations evolve and decision models will require continuous validation and calibration in contemporary population cohorts.

In conclusion, using an analytical framework based on contemporary UK population data and current best evidence for the beneficial and adverse effects of statin therapy, this study has shown that lifetime statin therapy is highly cost-effective across all adults 40–70 years old, suggesting that both widening of statin eligibility and improvements in statin uptake among eligible people need to be pursued.

## Contributors

BM and CB conceived the study. BM, IS, JE, CR, JR, AG, JA, CB secured funding. All authors contributed to study design and data interpretation. BM, RW, JZ, CW, IS performed the analyses. BM drafted the paper with support from RW, JZ and CW. All authors provided comments on the paper. BM acts as guarantor. The corresponding author attests that all listed authors meet authorship criteria and that no others meeting the criteria have been omitted.

The lead author (BM) affirms that the manuscript is an honest, accurate, and transparent account of the study being reported, and that no important aspects of the study have been omitted.

## Data sharing statement

Requests for individual patient data from trials contributing into the Cholesterol Treatment Trialists' Collaboration should be made directly to the data custodians of each trial (see Cholesterol Treatment Trialists’ data policy on https://www.cttcollaboration.org/). Other datasets used in the current study may be obtained from third parties (UK Biobank https://www.ukbiobank.ac.uk/; Whitehall II study www.ucl.ac.uk/epidemiology-health-care/research/epidemiology-and-public-health/research/whitehall-ii) and are not publicly available. Researchers can apply to use the UK Biobank resource and Whitehall II study data.

## Ethics statements

Ethics committee approval not required.

## Declaration of interests

BM reports research grant from the UK National Institute for Health Research and is a member (unpaid) of the European Society of Cardiology Clinical Practice Guidelines committee. RW, JZ and IS reports research support from the UK National Institute for Health Research. JE reports research funding from the UK National Institute for Health Research, British Heart Foundation, UK Medical Research Council. CR reports research funding from the UK National Institute for Health Research, British Heart Foundation, UK Medical Research Council and NHMRC Australia. AK research support from NHMRC Australia, Abbott, Amgen, Bayer, Mylan, Novartis, Sanofi, Viatris; speaker fees from Novartis; and is a Data Safety Monitoring Board member for Kowa. JA reports receiving a grant to their research institution from Novartis for the ORION 4 trial of inclisiran and. JS reports receiving grants for his institution from NHMRC Australia, Amgen, Bayer, BMS, MSD, Pfizer and Roche; consulting fees from FivepHusion, and is a chair (unpaid) of STAREE DSMB. CB reports research grants from UK National Institute for Health Research, UK Medical Research Council, Boehringer Ingelheim and Health Data Research UK, is a chair (unpaid) of a Data Safety Monitoring Board for Merck and a chair (unpaid) of the European Society of Cardiology Clinical Practice Guidelines committee. All other authors declare no competing interests.
