# Supplementary material for: Lifetime effects and cost-effectiveness of standard and higher-intensity statin therapy across population categories in the UK: a microsimulation modelling study
Source: Lancet Reg Health Eur. 2024 Mar 22;40:100887. doi: 10.1016/j.lanepe.2024.100887 (PMC10973720; doi:10.1016/j.lanepe.2024.100887)
Supplement: Supplementary methods, Tables S1–S7 and Figures S1–S7 [file mmc1.docx]

**Lifetime effects and cost-effectiveness of standard and higher-intensity statin therapy across population categories in the UK: a microsimulation modelling study**

**SUPPLEMENTARY MATERIAL**

Table of Contents

[Supplementary methods 2](#_Toc161384158)

[Supplementary Table S1: Proportional reductions in LDL cholesterol with statin regimens 12](#_Toc161384159)

[Supplementary Table S2: Number of UK Biobank participants, by sex, age, history of cardiovascular disease, 10-year cardiovascular risk and LDL cholesterol 13](#_Toc161384160)

[Supplementary Figure S1 Life years and QALYs gained with long-term statin therapy, by sex, age and 10-year cardiovascular risk 14](#_Toc161384161)

[Supplementary Figure S2 (a) Incremental cost-effectiveness ratios and (b) intervention with the highest probability of being cost-effective in categories of UK Biobank participants by sex, age, and 10-year cardiovascular risk 15](#_Toc161384162)

[Supplementary Table S3 Undiscounted and discounted life years and QALYs gained with statin therapy and incremental discounted healthcare costs (£) 16](#_Toc161384163)

[Supplementary Figure S3 Probability of statin therapy being cost-effective at different thresholds of cost-effectiveness (£/QALY) 23](#_Toc161384164)

[Supplementary Table S4 Impact of excess new diabetes due to statin therapy on QALYs gained 27](#_Toc161384165)

[Supplementary Figure S4 Sensitivity analyses of cost-effectiveness of standard statin versus no statin therapy 28](#_Toc161384166)

[Supplementary Figure S5 Sensitivity analyses of cost-effectiveness of higher intensity versus standard statin therapy 30](#_Toc161384167)

[Supplementary Figure S6 QALYs gained per person and cost-effectiveness of long-term statin therapy in categories by sex, age, pre-treatment LDL cholesterol level and cardiovascular risk: comparison of basecase and scenario analysis with variability around the proportional reduction in LDL-C with statin therapy 32](#_Toc161384168)

[Supplementary Table S5 Sensitivity analyses for cost-effectiveness (£/QALY) of statin therapy 33](#_Toc161384169)

[Supplementary Figure S7 Scenario analysis of QALYs gained and cost-effectiveness with long-term standard statin therapy in categories by sex, age, pre-treatment LDL cholesterol level and 10-year cardiovascular risk with added hypothetical disutility of daily pill of 0.002 QALYs/year 38](#_Toc161384170)

[Supplementary Table S6 A scenario analysis of stopping statin treatment at 80 years of age 39](#_Toc161384171)

[Supplementary Table S7 A scenario analysis of delaying statin treatment in 40 to 44 years old people without history of cardiovascular disease 41](#_Toc161384172)

[Supplementary references 42](#_Toc161384173)

## Supplementary methods

**Handling missing values in UK Biobank**

Following specification of participant characteristics, including those required for QRISK calculation, several characteristics, such as ethnicity, smoking status/cigarettes per day, BMI (including height, weight), total cholesterol, LDL cholesterol (LDL-C), HDL cholesterol, creatinine and blood pressure measures, and Townsend score, had some missing values (see Supplementary methods table 1). Missing ethnicities were imputed as white, the majority category. Missing smoking statuses were imputed with the majority smoking status by sex, age category and education level. Missing cigarettes per day were imputed with the majority level, i.e. 10-20. Missing Townsend scores were imputed by regressing on index of multiple deprivation (IMD) scores, years and sources (England, Wales or Scotland) if IMD scores were available, and imputed by looking up an average Townsend score according to the rounded ordnance survey coordinates if IMD scores were missing, or, finally, imputed by looking up an average Townsend score for the areas of the participant UK Biobank assessment centres if IMD scores and ordnance survey coordinates were both missing. The remaining continuous variables with missing values were imputed using multiple imputation by chained equations with 20 imputations and 10 iterations for each using the package “mice” in R, with weight, height, LDL-C, HDL cholesterol, triglycerides, creatinine, systolic blood pressure (two measures) and diastolic blood pressure (two measures), and with age, sex, (imputed) ethnicity, (imputed) smoking status, baseline cardiovascular diseases, treated hypertension, statin treatment status and diabetes added as auxiliary variables. Acknowledging the computational intensity of microsimulation model development and execution,^1^ the missing values for continuous factors were replaced with the mean values across the 20 imputed values. After missing data imputation, participants’ QRISK3 scores were calculated using an external R package “QRISK3”.^2^

**Supplementary methods table 1 Participants with missing data at baseline in UK Biobank (N = 501,854)**

|  | **Number participants with missing values** | **Participants with missing values (%)** |
| --- | --- | --- |
| Physical activity* | 99,996 | 19.9% |
| HDL cholesterol | 72,530 | 14.5% |
| LDL cholesterol | 33,744 | 6.7% |
| Creatinine | 33,092 | 6.6% |
| Diet quality | 11,018 | 2.2% |
| Smoking | 2,946 | 0.6% |
| Body mass index | 3,087 | 0.6% |
| Ethnicity | 2,771 | 0.6% |
| Systolic blood pressure | 1,319 | 0.3% |
| Diastolic blood pressure | 1,317 | 0.3% |
| Townsend score | 622 | 0.1% |
| *For QRISK3 calculation only* | | |
| Height (cm) | 3,087 | 0.6% |
| Weight (kg) | 2,757 | 0.5% |
| Total cholesterol/HDL | 72,530 | 14.5% |
| Standard deviation of systolic blood pressure | 1,826 | 0.4% |

*Missing values for physical activity level were not imputed but coded as a separate level. HDL, high-density lipoprotein; LDL, low-density lipoprotein

**Physical activity and diet specifications**

The **physical activity** level is indicated by the International Physical Activity Questionnaire (IPAQ) activity group, the UK Biobank derived variable based on Metabolic Equivalent Task (MET) scores and categorised following the IPAQ guidelines into three levels^3^:

**Low:** no activity or not enough to meet moderate or high level;

**Moderate:** 3 or more days of vigorous-intensity activity of at least 20 min/day/week, or

5 or more days of moderate-intensity activity and/or walking of at least 30 min/day/week, or

5 or more days of any combination of walking, moderate or vigorous-intensity activities achieving a minimum of at least 600 MET-min/week;

**High:** vigorous-intensity activity on at least 3 days and accumulating at least 1500 MET-min/week, or

7 or more days of any combination of walking, moderate or vigorous-intensity activities achieving a minimum of at least 3000 MET-min/week.

About 20% of the IPAQ values at entry into UK Biobank were missing; these are used in analyses as a separate category.

**Daily diet** is considered to be healthy if it meets at least 4 of the following 7 components^4^, otherwise unhealthy:

| **Component of food frequency** |
| --- |
| 1. Fruits: ≥ 3 servings/day |
| 1. Vegetables: ≥ 3 servings/day |
| 1. Fish: ≥2 servings/week |
| 1. Processed meats: ≤ 1 serving/week |
| 1. Unprocessed red meats: ≤ 1.5 servings/week |
| 1. Whole grains: ≥ 3 servings/day |
| 1. Refined grains: ≤1.5 servings/day |

The 11018 (2%) uncertain cases due to missing data in some categories of food intake were combined with the unhealthy diet category, as an early model indicated similar associations.

**Derivation of 10-year cardiovascular risk using QRISK3^5^ for participants at entry into the UK Biobank**

Further to participant characteristics previously defined or derived from UK Biobank, the following assumptions were made (**Supplementary methods table 2)**. First, we assumed that all unspecified black ethnicity (n=171) were black Caribbean. Second, at the entry into UK Biobank (2006-2010) few histories of CKD were coded as CKD stages 3-5 and many were coded as unspecified CKD; the whole chronic renal failure category were used to mean “CKD stage 3-5” for calculation of QRISK3. Third, the categories of depressive episode and recurrent depressive disorder were used to mean “moderate/severe depression” required for calculation of QRISK3, because many were coded as unspecified depression in UK Biobank. Fourth, we used history of heart disease of father, mother and sibling to substitute angina or heart attack in a 1st degree relative <60 required in QRISK3, as this is the closest information available in UK Biobank. The code lists of medications for antihypertension, erectile dysfunction treatment, regular steroid tablets and atypical antipsychotic treatment were sourced from a published study using UK Biobank data^6^.

**Supplementary methods table 2 QRISK3 risk factors’ specification using UK Biobank data**

| **QRISK3 risk factor** | **Risk factor specification in UK Biobank** |
| --- | --- |
| Sex | Original UK Biobank variable |
| Age | Must be 25-84. All UK Biobank participants are eligible at entry into UK Biobank. |
| Height (cm) | Original UK Biobank variable |
| Weight (kg) | Derived from BMI and height |
| Ethnicity | 1. White or not stated: white, British, Irish, any other white background, prefer not to answer, do not know 2. Indian: Indian 3. Pakistani: Pakistani 4. Bangladeshi: Bangladeshi 5. Chinese: Chinese 6. Other Asian: Asian or Asian British, any other Asian background 7. Black Caribbean: Caribbean, Black or Black British, any other Black background 8. Black African: African 9. Others: mixed, other ethnic group, white and black Caribbean, white and black African, white and Asian, any other mixed background |
| Townsend score | Original UK Biobank variable |
| Smoking status | 1. Non-smoker 2. Ex-smoker 3. Light smoker: <10 cigarettes/day 4. Moderate smoker: 10-19 cigarettes/day, or did not report 5. Heavy smoker: 20+ cigarettes/day |
| Diabetes type 1 | UK Biobank algorithm, ICD10: E10 insulin-dependent diabetes mellitus, E14 Unspecified diabetes mellitus (diagnosis age<=20 years) |
| Diabetes type 2 | UK Biobank algorithm, ICD10: E11 Non-insulin-dependent diabetes mellitus; E12 Malnutrition-related diabetes mellitus; E13 Other specified diabetes mellitus; E14 Unspecified diabetes mellitus (diagnosis age>20 years) |
| Atrial fibrillation | UK Biobank algorithm, ICD10: I48 Atrial fibrillation and flutter |
| Chronic kidney disease (stage 3,4 or5) | ICD10: N18 Chronic renal failure |
| Rheumatoid arthritis | UK Biobank algorithm, ICD10: M05 Seropositive rheumatoid arthritis; M06 Other rheumatoid arthritis |
| Migraine | UK Biobank algorithm, ICD10: G43 Migraine |
| Systemic lupus erythematosus | UK Biobank algorithm, ICD10: M32 Systemic lupus erythematosus |
| Severe mental illness (schizophrenia, bipolar disorder and moderate/severe depression) | UK Biobank algorithm, ICD10: F20 Schizophrenia; F23 Acute and transient psychotic disorders; F31 Bipolar affective disorder; F32 Depressive episode; F33 Recurrent depressive disorder |
| Erectile dysfunction or treatment | Combination of nurse interview medical conditions and medications data.^3^ |
| Angina or heart attack in a 1^st^ degree relative <60 | Including history of “heart disease” of father, mother and siblings. There is no information about whether the disease happened before 60 years old. Therefore, there are two assumptions: 1) heart disease were angina or heart attack, and 2) all the diseases happened under 60. |
| Total cholesterol/HDL | Total cholesterol/HDL |
| Systolic blood pressure | Mean, if there are two successive measures |
| Standard deviation of systolic blood pressure | Standard deviation of two successive measures |
| Blood pressure treatment | As defined in the main text |
| Regular steroid tablets | From the nurse interview’s medications data^6^ |
| Atypical antipsychotic medication | From the nurse interview’s medications data^6^ |

**Derivation of pre-treatment LDL-C levels for statin-treated UK Biobank participants**

We adjusted upwards the LDL-C levels of participants who were on statin treatment at entry into UK Biobank to derive “pre-treatment” LDL-C levels using the potency of their statin regimen (see Supplementary Table 1).

Statin dosage information was not collected at UK Biobank baseline interview but for participants with linked primary care prescription records both type of statin and dosage were available. However, more than half of the UK Biobank participants did not have linked primary care data, and, therefore, no linked primary care prescription records. Additionally, there were some discrepancies between reported statin use at UK Biobank recruitment and available statin prescription records; for such cases a report of statin use in either source was accepted and, if more than one source available, the more intensive regimen was used.

Reports of statin treatment with an exact statin medication and dosage were directly associated with the proportional LDL-C reductions according to Supplementary Table 1. Statin medications with unknown dosage were assumed to have the average proportional LDL-C reduction according to weighted frequencies, separately for categories of participants without and with history of cardiovascular disease, from respective participant categories for which exact statin medication and dose were available. There were few cases with combinations of two statins, where for those with known dosages, the more potent regimen was retained (one case with simvastatin 40mg and atorvastatin 10mg, we keep the latter), and for those with unknown dosage, the statin regimen retained followed the following hierarchy in decreasing order of rosuvastatin, atorvastatin, simvastatin, pravastatin, fluvastatin.

The **pre-treatment LDL cholesterol (LDL-C_pre-treatment_)** of statin-treated participants was calculated as:

LDL-C_pre-treatment_ = LDL-C_entry_ / (1 - %LDL-C reduction on statin treatment)

**The cardiovascular disease microsimulation policy model**

*Model structure*

Details of the cardiovascular disease microsimulation policy model are reported elsewhere^1^. Briefly, this cardiovascular disease microsimulation model projects cardiovascular disease, and key competing risks of diabetes, cancer and nonvascular death, over peoples’ lifetimes using a set of individual characteristics. The model simulates annually the first occurrences of seven disease endpoints: myocardial infarction (MI), stroke, coronary revascularisation (CRV), incident cancer, incident diabetes, vascular death, and nonvascular death (**Supplementary methods figure S1**). Risks of these endpoints are informed from parametric proportional hazards risk equations. These risk equations, except for diabetes, were initially estimated using individual participant data in the Cholesterol Treatment Trialists' (CTT) Collaboration (117,896 participants; 5 years follow-up).^7^ Subsequently, the risk equations were calibrated using the contemporary UK Biobank population cohort,^8^ comprising 501,854 participants followed up until March 2017. Separate risk equations were estimated for individuals without and with history of cardiovascular disease at baseline for model endpoints except for incident cancer and incident diabetes. These risk equations take into account baseline characteristics of individuals, including age, sex, ethnicity, body mass index (BMI), smoking status, blood pressure, lipids, Hemoglobin A1c (HbA1c) and creatinine levels, previous history of cardiovascular disease, treated hypertension, diabetes and cancer, mental illness, physical activity, diet quality, and socioeconomic deprivation.

In addition to the risk equations for the model's endpoints, the pooled data from the 2006, 2011, and 2017 Health Survey for England informed a linear regression model of the health-related quality of life (QoL) associated with participant characteristics, disease and event histories. The QoL was measured using the EuroQoL-5 Dimension (EQ-5D) utility, where a value of 0 represents death, a value of 1 perfect health and negative values represent health states worse than death. The linear regression model was integrated into the microsimulation model to annually assess QoL and, together with survival, inform quality-adjusted life years (QALYs).

Healthcare cost models, predicting annual primary and hospital care costs (2020£) associated with individual characteristics, disease histories and endpoints in the model, were integrated into the model to project long-term healthcare costs.^9^

*Model validation*

The cardiovascular disease policy model was validated using the UK Biobank cohort data, with about three further years of follow-up until February 2020, or up to 12 years of follow-up. Furthermore, the model was externally validated using the Whitehall II cohort (from phase 9 in the study), involving 6,761 individuals with about 10 years follow-up. In these validations, individual participant characteristics at baseline were used to simulate future disease risks and mortality. There was a generally strong agreement between the model's predicted cumulative incidence rates and the observed rates of endpoints across participant categories (**Supplementary methods** **figure S2**). While some overprediction of stroke risk is noted for the Whitehall II cohort, the overall microsimulation model performance, including of vascular and non-vascular mortality, was good. Whitehall II cohort was much smaller than UK Biobank and the identification of the stroke endpoint was exclusively based on linked hospital admissions and death registry data while a more comprehensive strategy, including some primary care data, was employed in UK Biobank. In view of the small cohort size, we did not consider further calibration of the stroke risk equation and the model was indicated.

*Model simulations*

For the purpose of model execution, the model inputs are provided in the form of individual patient baseline characteristics. Each individual is simulated on an annual basis, progressing through the model until they reach either death or the age of 110 years. Within each annual cycle, the model predicts in a random order the first occurrences of MI, stroke, CRV, incident diabetes and incident cancer, followed by the prediction in a random order of the vascular and nonvascular death endpoints. Age and event history are updated annually for each individual. The occurrence of events within the model influences the subsequent risks of events in a time-dependent manner. Individuals who have a history of cancer and/or diabetes at the beginning of the simulation are not considered at risk for incident cancer or diabetes in the model. To minimize the Monte Carlo error, we implemented 500 first-order simulations for each individual and averaged the outcomes across these simulations for each year.

The model was implemented using R 4.2.1. The R code of the cardiovascular disease policy model (License: MIT License) are available at http://www.herc.ox.ac.uk/downloads/supportingmaterial. To enhance the usability of the cardiovascular disease policy model, we have developed a visual user interface. The interface, along with a user guide, can be accessed at <https://livedataoxford.shinyapps.io/shiny_ctt_ukb_model/>. The model interface facilitates users to simulate long-term outcomes for individual profiles or a group of individuals.

**Supplementary methods figure S1 Schematic of the cardiovascular disease microsimulation model**


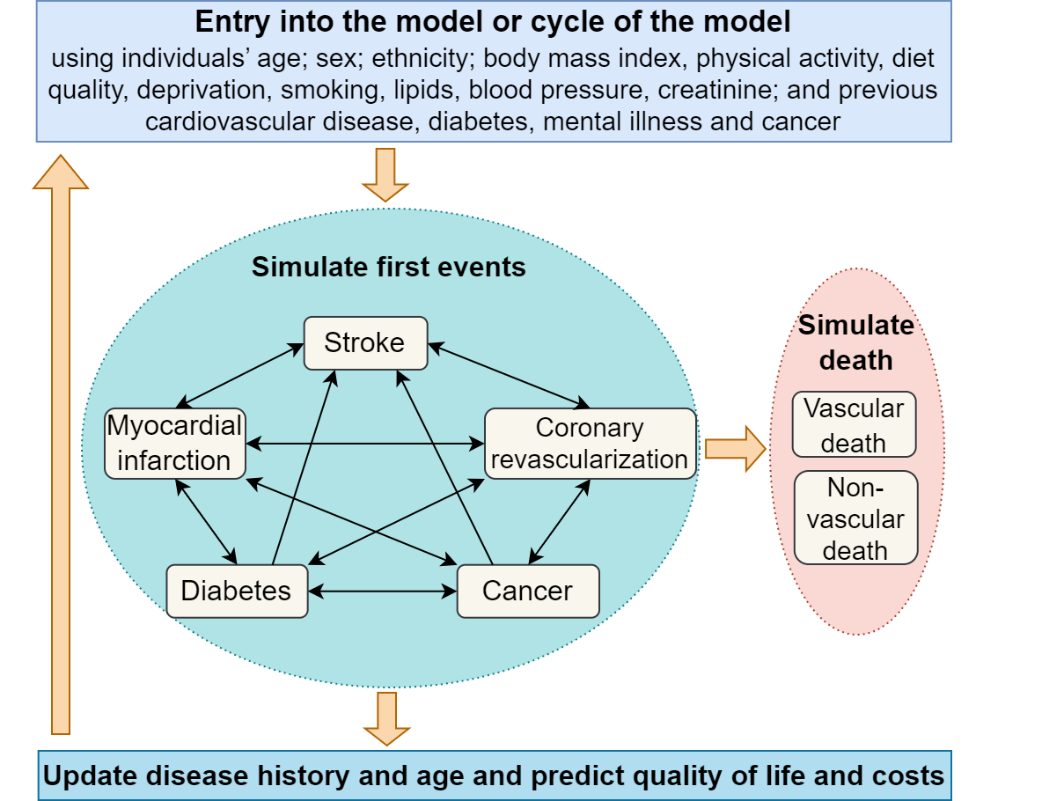


**Supplementary methods figure S2 Validation of the cardiovascular disease policy model**

**A. In UK Biobank cohort**


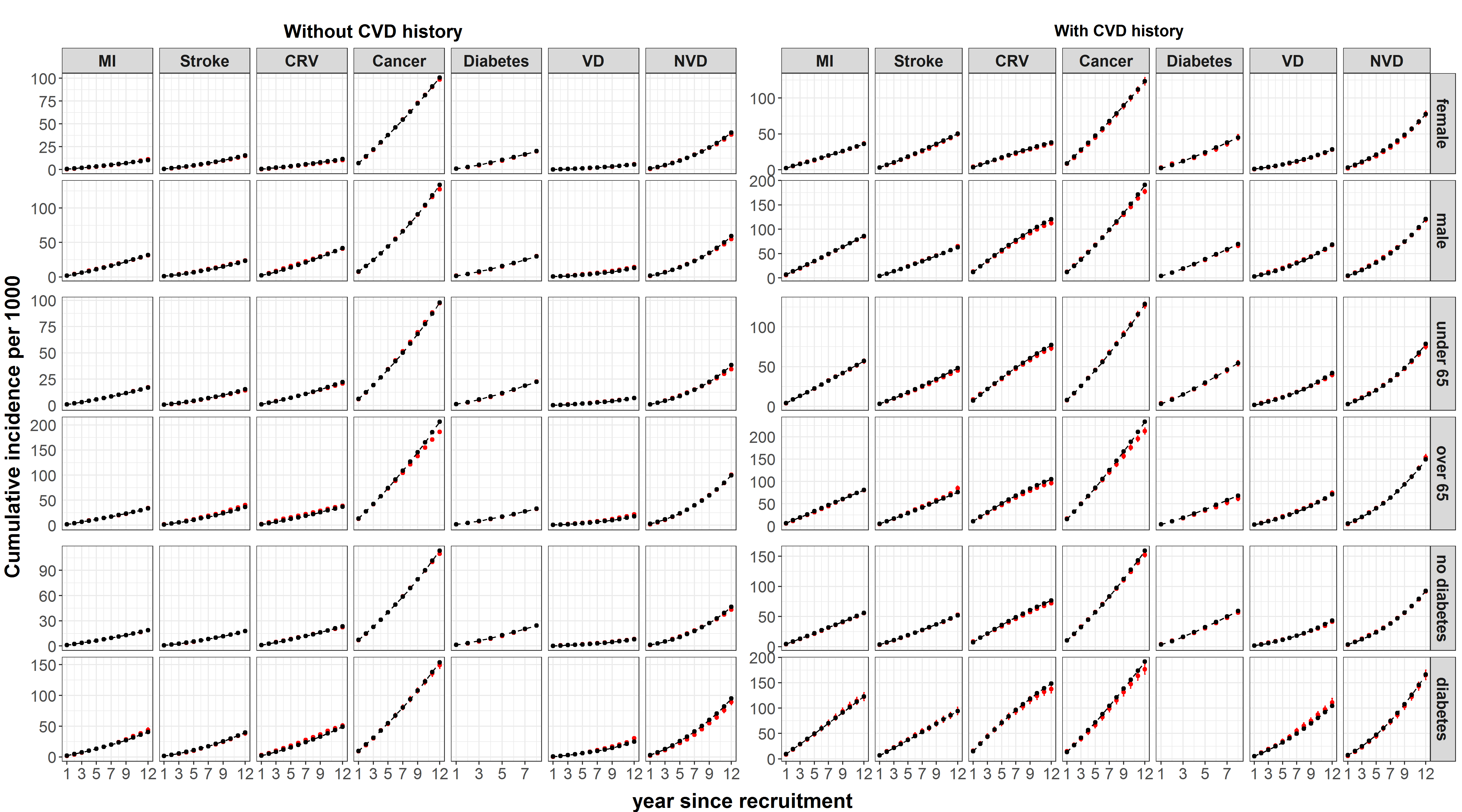


**B. In the Whitehall II cohort (Phase 9)**


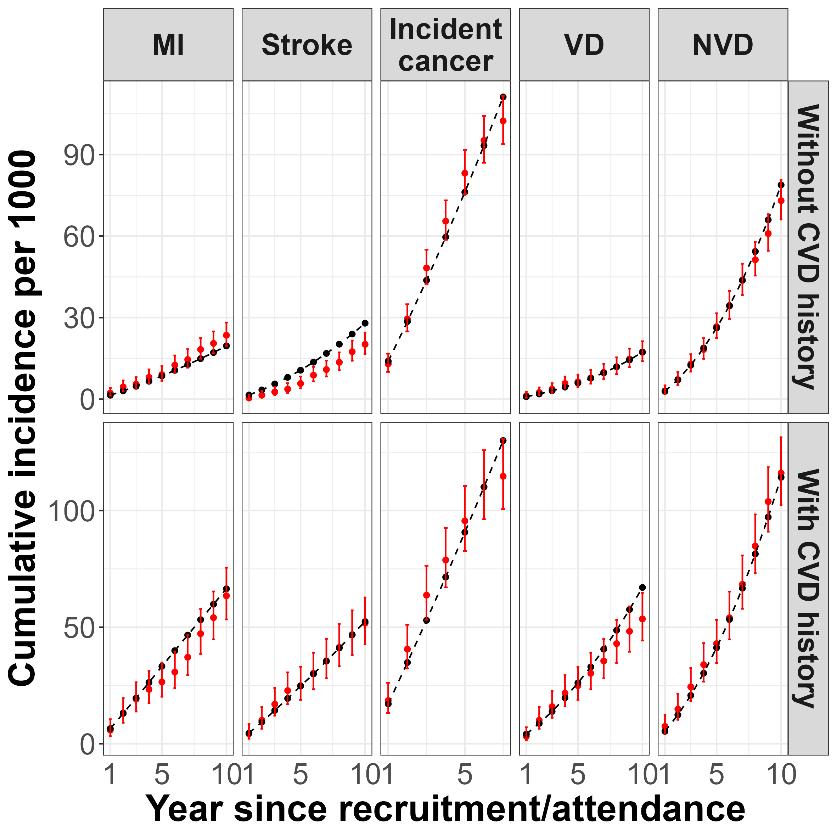


**RED=Observed; BLACK=model.** In the UK Biobank, validation covers 12 years including three extra years that were not used to calibrate the model. Follow-up of incident diabetes partly relies on primary care records, which ended earlier than other data types in the UK Biobank. In the Whitehall II Phase 9 data, validation covers 10 years (7 years for incidence cancer due to stopping follow-up earlier), and fewer categories were divided due to the relatively small number of this cohort. The endpoints of incident diabetes and CRV were not included in the validation in the Whitehall II data due to the lack of reliable follow-up data. MI, myocardial infarction; CRV, coronary revascularisation; VD, vascular death; NVD, non-vascular death.

**Integrating treatment effects of statin therapy in the cardiovascular disease microsimulation model**

The rate ratios (RR (CI)) per 1mmol/L reduction in LDL-C with statin therapy, as reported by Cholesterol Treatment Trialists’ Collaborative meta-analysis of individual participant data from large randomised controlled trials of statin therapy,^10^ informed effects on cardiovascular events. It is of note that each of the trials in the meta-analysis had at least 1000 people and, therefore, the uncertainty in mean LDL-C reduction between arms is negligible. Further meta-analyses of randomised controlled trials of statin therapy informed effects of statin therapy on incident diabetes in the model^11, 12^ (see Table 1 in the manuscript). In the base case analysis, it was assumed that statins do not affect cancer incidence and non-vascular death.

The transition probabilities (tp) of events in the absence of statin treatment in the model in each cycle are calculated as:

**tp(t_u_) = 1 – exp[H(t-u) – H(t)]**, where

u is the length of the cycle (i.e. 1 year), H(t-u) and H(t) are the cumulative hazards at time t-u and t, respectively, and t_u_ is the cycle from t-u to t.

The treatment effects of statin (tx) are calculated as: **tx = exp[ALR * ln(RR)]**, where RR is the rate ratio per 1 mmol/L LDL-C reduction with statin (see Table 1 in the manuscript) and ALR is the absolute LDL-C reduction with the statin therapy, which is product of pre-treatment LDL-C level and the proportional reduction in LDL-C with corresponding statin regimen (see Supplementary Table 1). It has been previously shown that the absolute reductions in LDL-C (in mmol/l) were greater in individuals with higher pre-treatment LDL-C concentrations and the percentage reductions were independent of pre-treatment concentrations and therefore more generalizable.^13^

The transition probabilities for events with statin treatment (tp_tx_) in each cycle of the model is calculated as:

**tp_tx_(t_u_) = 1 – exp[H(t-u) – H(t)]^tx^**

The excess rates on myopathy and rhabdomyolysis of statin treatment (see Table 1 in the manuscript) were applied as constant annual rate each year on statin treatment in the model.

**Specification of sensitivity and scenario analyses**

**Supplementary methods table 3: Sensitivity analyses parameterisation in the model**

| **Scenario** | **Parameters** |
| --- | --- |
| **Relative risk reduction in cardiovascular events with statin therapy increase/decrease annually** | Increase: further 1.5% relative risk reduction per 1 mmol/L reduction of LDL-C^14^ added each year from year 6 onwards.  Decrease: Relative risk reduction (RRR) reduced by 5% each year from year 6 onwards.  Lifetime statin use and statin costs retained in simulations. |
| **Reduced statin treatment effects on cardiovascular events in the elderly** | Rate ratios (RR) per 1mmol/L LDL-C reduction based on effects only among participants >75 years of age in the Cholesterol Treatment Trialists’ individual participant data meta-analysis^10^ were applied in people from 76 years of age onward in model simulation. The applied effects were:  Myocardial infarction: Major coronary event RR 0.82 (99% confidence interval (CI) 0.70–0.96)  Stroke: RR 0.89 (99% CI 0.71, 1.10)  Coronary revascularisation procedure: RR 1.02 (99% CI 0.75, 1.40)  Vascular death: RR 0.95 (99% CI 0.83, 1.07) |
| **LDL reduction with statin therapy lower than expected** | Proportional reduction in LDL-C with statin therapy assumed 80% of expected reduction described in Supplementary table 1. |
| **Vary statin treatment effect on cancer incidence** | Rate ratio (RR) of 0.96 or 1.05, respectively, applied for incident cancer with statin therapy based on 95% confidence interval of the Cholesterol Treatment Trialists’ individual participant data meta-analysis reporting RR of 1.00 [95% CI 0.96-1.05].^15^ |
| **Real-world compliance with statin therapy** | Using observed statin discontinuation and restarting rates for the first discontinuation and first restarting^16^, the derived probabilities of complying with statin therapy (Supplementary methods table 3) were applied to each individual in the respective years in model simulation. Both statin effects and costs discontinued with no statin use. |
| **Quality of life disutilities of daily statin pill** | 0.001, 0.002 or 0.003 QALYs were deducted each year in the model^17^ |
| **Quality of life disutilities of cardiovascular events** | 50% or 150% of base-case decrements in quality of life related to cardiovascular events were applied |
| **Quality of life disutilities of diabetes** | Apply 50% of basecase decrement in quality of life related to diabetes |
| **Discount rates for costs and outcomes of 1.5%** | Annual discount rates to 1.5% were used for costs and QALYs (instead of the 3.5% base-case rates)^18^ |
| **Include healthcare costs only for cardiovascular disease and incident diabetes** | Healthcare costs associated with cardiovascular disease and incident diabetes only included (i.e., unrelated healthcare costs were excluded). |
| **Increased cost of statin therapy** | The base-case costs of statin therapy increased 1.5, 2 or 5 times. |
| **Including variability around the proportional reductions in LDL-C with statin therapies** | The % LDL-C reductions sampled from normal distributions with mean 43% (standard deviation 14.5%) for standard statin and mean 55% (standard deviation 17.3%) for higher intensity statin therapy with standard deviations sourced from meta-analysis^19^. |

**Supplementary methods table 4: Probabilities for first discontinuation and first restarting of statin treatment and the derived probabilities of compliance with statin therapy over the first 10 years**

|  | Cumulative probability (%) | | On statin treatment (%) | |
| --- | --- | --- | --- | --- |
| Year | Discontinuation | Restarting | On | Off |
| 1 | 30% | 50% | 70% | 30% |
| 2 | 38% | 59% | 77% | 23% |
| 3 | 43% | 64% | 79% | 21% |
| 4 | 47% | 68% | 80% | 20% |
| 5 | 50% | 70% | 81% | 19% |
| 6 | 52% | 72% | 81% | 19% |
| 7 | 54% | 74% | 82% | 18% |
| 8 | 56% | 76% | 82% | 18% |
| 9 | 58% | 77% | 83% | 17% |
| 10 | 60% | 79% | 83% | 17% |

The first two columns present cumulative probabilities for the first discontinuation and first restarting of statin treatment^16^, followed by the derived compliance with statin treatment in first 10 years of treatment.

## Supplementary Table S1: Proportional reductions in LDL cholesterol with statin regimens

|  | **% reduction in LDL cholesterol^1^** | | | | |
| --- | --- | --- | --- | --- | --- |
| **Dose (mg/day)** | **5mg** | **10mg** | **20mg** | **40mg** | **80mg** |
| Fluvastatin | 10% | 15% | 21% | 27% | 33% |
| Pravastatin | 15% | 20% | 24% | 29% | 33% |
| Simvastatin | 23% | 27% | 32% | 37% | 42% |
| Atorvastatin | 31% | 37% | 43% | 49% | 55% |
| Rosuvastatin | 38% | 43% | 48% | 53% | 58% |

^1^Based on Law et al.^13^

LDL, low density lipoprotein

## Supplementary Table S2: Number of UK Biobank participants, by sex, age, history of cardiovascular disease, 10-year cardiovascular risk and LDL cholesterol

1. By sex, age, history of CVD and 10-year CVD risk

|  | **Without CVD, by 10-year CVD risk (%)** | | | | | **with CVD** |
| --- | --- | --- | --- | --- | --- | --- |
|  | **<5** | **5-10** | **10-15** | **15-20** | **≥20** |  |
| **Men** |  |  |  |  |  |  |
| **40-49** | 29,556 | 14,976 | 3,415 | 1,216 | 1,177 | 2,798 |
| **50-59** | 3,959 | 24,290 | 18,565 | 8,970 | 9,035 | 8,161 |
| **60-70** | n/a | 2,703 | 14,883 | 20,150 | 42,101 | 22,775 |
| **Women** |  |  |  |  |  |  |
| **40-49** | 57,059 | 3,447 | 619 | 247 | 419 | 2,814 |
| **50-59** | 46,336 | 30,155 | 6,579 | 2,128 | 2,120 | 6,622 |
| **60-70** | 3,394 | 36,730 | 31,390 | 15,194 | 13,763 | 14,108 |

1. By sex, age, LDL cholesterol, history of CVD and 10-year CVD risk

|  | **LDL cholesterol (mmol/L)** | | | | | | | | | | | | | | | | | | | |
| --- | --- | --- | --- | --- | --- | --- | --- | --- | --- | --- | --- | --- | --- | --- | --- | --- | --- | --- | --- | --- |
|  | **<3.4** | | | | | | **3.4-4.1** | | | | | | | **≥4.1** | | | | | |  |
|  | **Without CVD, by 10-year CVD risk (%)** | | | | | **with CVD** | **Without CVD, by 10-year CVD risk (%)** | | | | | **with CVD** | **Without CVD, by 10-year CVD risk (%)** | | | | | | **with CVD** |  |
|  | **<5** | **5-10** | **10-15** | **15-20** | **≥20** |  | **<5** | **5-10** | **10-15** | **15-20** | **≥20** |  | **<5** | | **5-10** | **10-15** | **15-20** | **≥20** |  |  |
| **Men** |  |  |  |  |  |  |  |  |  |  |  |  |  | |  |  |  |  |  |  |
| **40-49** | 13,497 | 4,037 | 711 | 239 | 228 | 939 | 10,436 | 5,629 | 1,099 | 332 | 289 | 853 | 5,623 | | 5,310 | 1,605 | 645 | 660 | 1,006 |  |
| **50-59** | 2,281 | 9,055 | 4,759 | 1,847 | 1,622 | 2,263 | 1,255 | 9,445 | 7,190 | 3,079 | 2,408 | 2,317 | 423 | | 5,790 | 6,616 | 4,044 | 5,005 | 3,581 |  |
| **60-70** | n/a | 1,535 | 6,073 | 6,513 | 9,113 | 6,218 | n/a | 908 | 5,919 | 8,003 | 14,028 | 6,607 | n/a | | 260^1^ | 2,891 | 5,634 | 18,960 | 9,950 |  |
| **Women** |  |  |  |  |  |  |  |  |  |  |  |  |  | |  |  |  |  |  |  |
| **40-49** | 32,831 | 986 | 146 | 60 | 119 | 1,430 | 17,285 | 1,214 | 178 | 59 | 86 | 827 | 6,943 | | 1,247 | 295 | 128 | 214 | 557 |  |
| **50-59** | 18,843 | 6,707 | 1,025 | 308 | 366 | 1,868 | 17,471 | 11,228 | 1,980 | 540 | 454 | 2,109 | 10,022 | | 12,220 | 3,574 | 1,280 | 1,300 | 2,645 |  |
| **60-70** | 1,522 | 9,822 | 5,742 | 1,994 | 1,589 | 2,956 | 1,304 | 14,829 | 11,580 | 4,693 | 3,039 | 4,298 | 568 | | 12,079 | 14,068 | 8,507 | 9,135 | 6,854 |  |

CVD, cardiovascular disease. Categories of participants 40-49 years old at estimated 10-year CVD risk ≥10% combined for the presentation of results. n/a, not applicable. LDL, low density lipoprotein.

## Supplementary Figure S1 Life years and QALYs gained with long-term statin therapy, by sex, age and 10-year cardiovascular risk


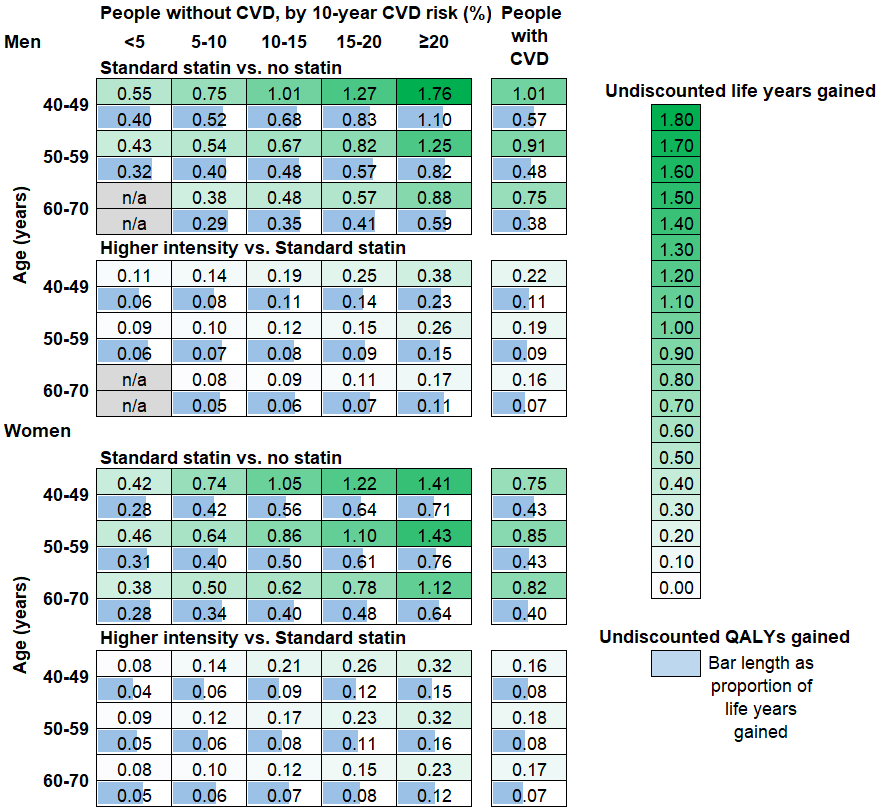


QALY, quality-adjusted life year; CVD, cardiovascular disease; n/a, not applicable.

## Supplementary Figure S2 (a) Incremental cost-effectiveness ratios and (b) intervention with the highest probability of being cost-effective in categories of UK Biobank participants by sex, age, and 10-year cardiovascular risk

1. **Incremental cost-effectiveness ratios (£/QALY gained)**


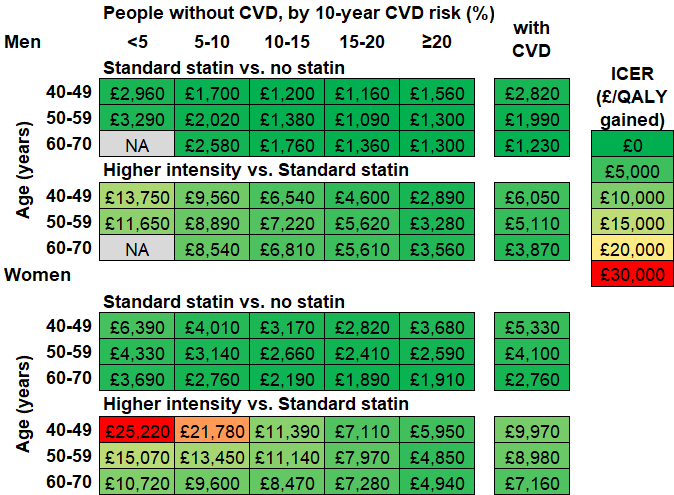


**(b) Intervention with the highest probability of being cost-effective at £20,000/QALY**
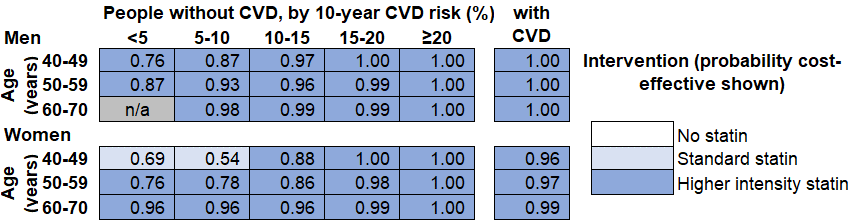


CVD, cardiovascular disease; ICER, Incremental Cost-Effectiveness Ratio (with costs and QALYs discounted at 3.5% per year); QALY, quality-adjusted life years; n/a, not applicable.

## Supplementary Table S3 Undiscounted and discounted life years and QALYs gained with statin therapy and incremental discounted healthcare costs (£)

| LDL-C | Sex | Statin therapy | Age | 10-year CVD risk/ with CVD | Life years gained, discounted | Life years gained, undiscounted | QALYs gained, undiscounted | QALYs gained, discounted | Total cost (£), discounted | Incremental hospital care costs (£), discounted | Incremental primary care costs (£), discounted | Statin cost (£), discounted | Statin initiation and monitoring cost (£), discounted |
| --- | --- | --- | --- | --- | --- | --- | --- | --- | --- | --- | --- | --- | --- |
| <3.4 | men | Standard | 40-49 | <5 | 0.38 | 0.09 | 0.28 | 0.07 | 331 | -65 | 29 | 304 | 64 |
| <3.4 | men | Standard | 40-49 | [5,10) | 0.46 | 0.13 | 0.32 | 0.09 | 310 | -99 | 54 | 288 | 66 |
| <3.4 | men | Standard | 40-49 | [10,15) | 0.64 | 0.20 | 0.40 | 0.13 | 361 | -123 | 141 | 273 | 70 |
| <3.4 | men | Standard | 40-49 | with CVD | 0.64 | 0.22 | 0.38 | 0.14 | 539 | -169 | 158 | 271 | 279 |
| <3.4 | men | Standard | 50-59 | <5 | 0.33 | 0.09 | 0.25 | 0.07 | 322 | -50 | 23 | 286 | 63 |
| <3.4 | men | Standard | 50-59 | [5,10) | 0.36 | 0.11 | 0.26 | 0.08 | 285 | -84 | 31 | 273 | 65 |
| <3.4 | men | Standard | 50-59 | [10,15) | 0.41 | 0.13 | 0.28 | 0.10 | 265 | -112 | 50 | 260 | 67 |
| <3.4 | men | Standard | 50-59 | [15,20) | 0.49 | 0.17 | 0.32 | 0.12 | 273 | -131 | 84 | 251 | 69 |
| <3.4 | men | Standard | 50-59 | ≥20 | 0.62 | 0.24 | 0.39 | 0.16 | 367 | -127 | 182 | 239 | 72 |
| <3.4 | men | Standard | 50-59 | with CVD | 0.60 | 0.26 | 0.33 | 0.14 | 494 | -198 | 213 | 233 | 246 |
| <3.4 | men | Standard | 60-70 | [5,10) | 0.29 | 0.10 | 0.22 | 0.08 | 277 | -63 | 23 | 253 | 64 |
| <3.4 | men | Standard | 60-70 | [10,15) | 0.33 | 0.12 | 0.24 | 0.09 | 257 | -85 | 34 | 243 | 65 |
| <3.4 | men | Standard | 60-70 | [15,20) | 0.37 | 0.15 | 0.26 | 0.11 | 249 | -101 | 51 | 233 | 67 |
| <3.4 | men | Standard | 60-70 | ≥20 | 0.47 | 0.20 | 0.31 | 0.14 | 290 | -112 | 116 | 216 | 70 |
| <3.4 | men | Standard | 60-70 | with CVD | 0.54 | 0.27 | 0.28 | 0.14 | 432 | -213 | 230 | 199 | 216 |
| <3.4 | men | Higher intensity | 40-49 | <5 | 0.07 | 0.02 | 0.04 | 0.01 | 191 | -7 | 37 | 161 | -1 |
| <3.4 | men | Higher intensity | 40-49 | [5,10) | 0.09 | 0.02 | 0.04 | 0.01 | 199 | -9 | 56 | 153 | -1 |
| <3.4 | men | Higher intensity | 40-49 | ≥10 | 0.12 | 0.04 | 0.06 | 0.02 | 194 | -21 | 71 | 145 | -1 |
| <3.4 | men | Higher intensity | 40-49 | with CVD | 0.14 | 0.05 | 0.07 | 0.02 | 207 | -13 | 76 | 144 | 1 |
| <3.4 | men | Higher intensity | 50-59 | <5 | 0.07 | 0.02 | 0.04 | 0.01 | 176 | -4 | 29 | 151 | -0 |
| <3.4 | men | Higher intensity | 50-59 | [5,10) | 0.07 | 0.02 | 0.04 | 0.01 | 169 | -11 | 36 | 144 | -1 |
| <3.4 | men | Higher intensity | 50-59 | [10,15) | 0.07 | 0.02 | 0.04 | 0.01 | 169 | -16 | 48 | 138 | -1 |
| <3.4 | men | Higher intensity | 50-59 | [15,20) | 0.09 | 0.03 | 0.05 | 0.02 | 171 | -19 | 58 | 133 | -1 |
| <3.4 | men | Higher intensity | 50-59 | ≥20 | 0.13 | 0.05 | 0.08 | 0.03 | 165 | -26 | 65 | 127 | -1 |
| <3.4 | men | Higher intensity | 50-59 | with CVD | 0.13 | 0.06 | 0.06 | 0.03 | 191 | -17 | 84 | 124 | 1 |
| <3.4 | men | Higher intensity | 60-70 | [5,10) | 0.06 | 0.02 | 0.04 | 0.01 | 151 | -8 | 25 | 134 | -0 |
| <3.4 | men | Higher intensity | 60-70 | [10,15) | 0.06 | 0.02 | 0.04 | 0.01 | 148 | -12 | 32 | 129 | -1 |
| <3.4 | men | Higher intensity | 60-70 | [15,20) | 0.07 | 0.03 | 0.04 | 0.02 | 147 | -16 | 40 | 123 | -1 |
| <3.4 | men | Higher intensity | 60-70 | ≥20 | 0.10 | 0.04 | 0.06 | 0.02 | 146 | -21 | 53 | 115 | -1 |
| <3.4 | men | Higher intensity | 60-70 | with CVD | 0.12 | 0.06 | 0.05 | 0.03 | 164 | -27 | 83 | 106 | 1 |
| <3.4 | women | Standard | 40-49 | <5 | 0.31 | 0.06 | 0.21 | 0.05 | 393 | -14 | 31 | 315 | 61 |
| <3.4 | women | Standard | 40-49 | [5,10) | 0.45 | 0.11 | 0.25 | 0.07 | 441 | -12 | 95 | 294 | 64 |
| <3.4 | women | Standard | 40-49 | ≥10 | 0.53 | 0.15 | 0.28 | 0.09 | 512 | -1 | 162 | 286 | 65 |
| <3.4 | women | Standard | 40-49 | with CVD | 0.58 | 0.16 | 0.35 | 0.10 | 670 | -25 | 109 | 291 | 296 |
| <3.4 | women | Standard | 50-59 | <5 | 0.32 | 0.08 | 0.22 | 0.06 | 362 | -28 | 33 | 295 | 62 |
| <3.4 | women | Standard | 50-59 | [5,10) | 0.38 | 0.11 | 0.24 | 0.07 | 378 | -28 | 63 | 281 | 63 |
| <3.4 | women | Standard | 50-59 | [10,15) | 0.46 | 0.14 | 0.26 | 0.08 | 419 | -25 | 112 | 267 | 65 |
| <3.4 | women | Standard | 50-59 | [15,20) | 0.57 | 0.18 | 0.30 | 0.10 | 445 | -43 | 154 | 268 | 67 |
| <3.4 | women | Standard | 50-59 | ≥20 | 0.61 | 0.20 | 0.32 | 0.11 | 516 | -26 | 216 | 259 | 68 |
| <3.4 | women | Standard | 50-59 | with CVD | 0.61 | 0.21 | 0.33 | 0.12 | 661 | -41 | 165 | 265 | 273 |
| <3.4 | women | Standard | 60-70 | <5 | 0.28 | 0.08 | 0.20 | 0.06 | 314 | -49 | 24 | 277 | 62 |
| <3.4 | women | Standard | 60-70 | [5,10) | 0.32 | 0.10 | 0.22 | 0.07 | 319 | -53 | 42 | 267 | 63 |
| <3.4 | women | Standard | 60-70 | [10,15) | 0.36 | 0.12 | 0.23 | 0.08 | 330 | -56 | 66 | 255 | 64 |
| <3.4 | women | Standard | 60-70 | [15,20) | 0.42 | 0.15 | 0.26 | 0.10 | 351 | -58 | 98 | 246 | 66 |
| <3.4 | women | Standard | 60-70 | ≥20 | 0.51 | 0.19 | 0.28 | 0.12 | 398 | -72 | 166 | 235 | 68 |
| <3.4 | women | Standard | 60-70 | with CVD | 0.61 | 0.25 | 0.30 | 0.12 | 628 | -70 | 216 | 235 | 248 |
| <3.4 | women | Higher intensity | 40-49 | <5 | 0.06 | 0.01 | 0.03 | 0.01 | 199 | 4 | 29 | 167 | -0 |
| <3.4 | women | Higher intensity | 40-49 | [5,10) | 0.08 | 0.02 | 0.03 | 0.01 | 240 | 16 | 68 | 155 | -0 |
| <3.4 | women | Higher intensity | 40-49 | ≥10 | 0.12 | 0.03 | 0.06 | 0.02 | 221 | 11 | 59 | 151 | -0 |
| <3.4 | women | Higher intensity | 40-49 | with CVD | 0.13 | 0.04 | 0.07 | 0.02 | 214 | 9 | 50 | 154 | 0 |
| <3.4 | women | Higher intensity | 50-59 | <5 | 0.06 | 0.01 | 0.04 | 0.01 | 187 | 1 | 30 | 156 | -0 |
| <3.4 | women | Higher intensity | 50-59 | [5,10) | 0.07 | 0.02 | 0.03 | 0.01 | 206 | 8 | 51 | 148 | -0 |
| <3.4 | women | Higher intensity | 50-59 | [10,15) | 0.09 | 0.03 | 0.04 | 0.01 | 224 | 13 | 71 | 141 | -1 |
| <3.4 | women | Higher intensity | 50-59 | [15,20) | 0.13 | 0.04 | 0.06 | 0.02 | 208 | 1 | 65 | 142 | -1 |
| <3.4 | women | Higher intensity | 50-59 | ≥20 | 0.15 | 0.05 | 0.08 | 0.03 | 198 | 1 | 60 | 137 | -1 |
| <3.4 | women | Higher intensity | 50-59 | with CVD | 0.14 | 0.04 | 0.06 | 0.02 | 227 | 15 | 70 | 141 | 1 |
| <3.4 | women | Higher intensity | 60-70 | <5 | 0.06 | 0.02 | 0.04 | 0.01 | 162 | -6 | 22 | 147 | -0 |
| <3.4 | women | Higher intensity | 60-70 | [5,10) | 0.06 | 0.02 | 0.04 | 0.01 | 174 | -2 | 35 | 141 | -0 |
| <3.4 | women | Higher intensity | 60-70 | [10,15) | 0.07 | 0.02 | 0.04 | 0.01 | 187 | 3 | 50 | 135 | -0 |
| <3.4 | women | Higher intensity | 60-70 | [15,20) | 0.08 | 0.03 | 0.04 | 0.01 | 197 | 6 | 62 | 130 | -1 |
| <3.4 | women | Higher intensity | 60-70 | ≥20 | 0.11 | 0.04 | 0.06 | 0.02 | 178 | -6 | 60 | 125 | -1 |
| <3.4 | women | Higher intensity | 60-70 | with CVD | 0.14 | 0.05 | 0.06 | 0.02 | 230 | 17 | 87 | 125 | 1 |
| 3.4-4.1 | men | Standard | 40-49 | <5 | 0.58 | 0.14 | 0.43 | 0.11 | 287 | -116 | 32 | 306 | 65 |
| 3.4-4.1 | men | Standard | 40-49 | [5,10) | 0.67 | 0.18 | 0.46 | 0.13 | 266 | -154 | 60 | 293 | 67 |
| 3.4-4.1 | men | Standard | 40-49 | ≥10 | 0.89 | 0.28 | 0.58 | 0.19 | 328 | -180 | 160 | 277 | 71 |
| 3.4-4.1 | men | Standard | 40-49 | with CVD | 0.89 | 0.32 | 0.52 | 0.19 | 556 | -223 | 234 | 269 | 276 |
| 3.4-4.1 | men | Standard | 50-59 | <5 | 0.50 | 0.14 | 0.38 | 0.11 | 284 | -95 | 26 | 289 | 64 |
| 3.4-4.1 | men | Standard | 50-59 | [5,10) | 0.54 | 0.16 | 0.40 | 0.12 | 249 | -130 | 36 | 277 | 66 |
| 3.4-4.1 | men | Standard | 50-59 | [10,15) | 0.60 | 0.19 | 0.42 | 0.14 | 233 | -158 | 57 | 266 | 68 |
| 3.4-4.1 | men | Standard | 50-59 | [15,20) | 0.68 | 0.24 | 0.46 | 0.17 | 237 | -180 | 91 | 257 | 69 |
| 3.4-4.1 | men | Standard | 50-59 | ≥20 | 0.86 | 0.33 | 0.54 | 0.22 | 355 | -172 | 211 | 243 | 73 |
| 3.4-4.1 | men | Standard | 50-59 | with CVD | 0.78 | 0.33 | 0.43 | 0.18 | 416 | -321 | 250 | 238 | 249 |
| 3.4-4.1 | men | Standard | 60-70 | [5,10) | 0.45 | 0.15 | 0.34 | 0.12 | 252 | -98 | 28 | 258 | 64 |
| 3.4-4.1 | men | Standard | 60-70 | [10,15) | 0.50 | 0.18 | 0.37 | 0.14 | 227 | -127 | 39 | 249 | 66 |
| 3.4-4.1 | men | Standard | 60-70 | [15,20) | 0.54 | 0.21 | 0.39 | 0.16 | 212 | -150 | 56 | 239 | 67 |
| 3.4-4.1 | men | Standard | 60-70 | ≥20 | 0.67 | 0.28 | 0.45 | 0.20 | 278 | -145 | 130 | 223 | 70 |
| 3.4-4.1 | men | Standard | 60-70 | with CVD | 0.68 | 0.34 | 0.36 | 0.18 | 313 | -365 | 255 | 203 | 219 |
| 3.4-4.1 | men | Higher intensity | 40-49 | <5 | 0.11 | 0.03 | 0.07 | 0.02 | 188 | -16 | 43 | 162 | -1 |
| 3.4-4.1 | men | Higher intensity | 40-49 | [5,10) | 0.12 | 0.03 | 0.07 | 0.02 | 193 | -22 | 61 | 155 | -1 |
| 3.4-4.1 | men | Higher intensity | 40-49 | ≥10 | 0.17 | 0.05 | 0.09 | 0.03 | 197 | -31 | 82 | 147 | -1 |
| 3.4-4.1 | men | Higher intensity | 40-49 | with CVD | 0.20 | 0.07 | 0.10 | 0.04 | 224 | -16 | 96 | 143 | 1 |
| 3.4-4.1 | men | Higher intensity | 50-59 | <5 | 0.10 | 0.03 | 0.07 | 0.02 | 172 | -13 | 33 | 153 | -1 |
| 3.4-4.1 | men | Higher intensity | 50-59 | [5,10) | 0.10 | 0.03 | 0.07 | 0.02 | 168 | -18 | 40 | 147 | -1 |
| 3.4-4.1 | men | Higher intensity | 50-59 | [10,15) | 0.11 | 0.03 | 0.06 | 0.02 | 169 | -24 | 53 | 141 | -1 |
| 3.4-4.1 | men | Higher intensity | 50-59 | [15,20) | 0.12 | 0.04 | 0.07 | 0.02 | 172 | -28 | 65 | 136 | -1 |
| 3.4-4.1 | men | Higher intensity | 50-59 | ≥20 | 0.18 | 0.07 | 0.10 | 0.04 | 179 | -34 | 85 | 130 | -1 |
| 3.4-4.1 | men | Higher intensity | 50-59 | with CVD | 0.17 | 0.07 | 0.08 | 0.03 | 191 | -36 | 100 | 127 | 1 |
| 3.4-4.1 | men | Higher intensity | 60-70 | [5,10) | 0.09 | 0.03 | 0.06 | 0.02 | 150 | -12 | 26 | 137 | -1 |
| 3.4-4.1 | men | Higher intensity | 60-70 | [10,15) | 0.10 | 0.03 | 0.06 | 0.02 | 146 | -19 | 34 | 132 | -1 |
| 3.4-4.1 | men | Higher intensity | 60-70 | [15,20) | 0.10 | 0.04 | 0.07 | 0.02 | 144 | -23 | 42 | 127 | -1 |
| 3.4-4.1 | men | Higher intensity | 60-70 | ≥20 | 0.13 | 0.05 | 0.08 | 0.03 | 151 | -28 | 61 | 119 | -1 |
| 3.4-4.1 | men | Higher intensity | 60-70 | with CVD | 0.14 | 0.07 | 0.07 | 0.03 | 146 | -55 | 91 | 109 | 1 |
| 3.4-4.1 | women | Standard | 40-49 | <5 | 0.49 | 0.10 | 0.32 | 0.07 | 398 | -27 | 49 | 316 | 61 |
| 3.4-4.1 | women | Standard | 40-49 | [5,10) | 0.64 | 0.16 | 0.35 | 0.09 | 455 | -27 | 123 | 296 | 64 |
| 3.4-4.1 | women | Standard | 40-49 | ≥10 | 0.87 | 0.24 | 0.44 | 0.13 | 603 | 1 | 247 | 289 | 66 |
| 3.4-4.1 | women | Standard | 40-49 | with CVD | 0.77 | 0.22 | 0.45 | 0.14 | 668 | -65 | 150 | 289 | 294 |
| 3.4-4.1 | women | Standard | 50-59 | <5 | 0.47 | 0.12 | 0.32 | 0.08 | 359 | -45 | 45 | 297 | 62 |
| 3.4-4.1 | women | Standard | 50-59 | [5,10) | 0.54 | 0.15 | 0.34 | 0.10 | 377 | -50 | 80 | 284 | 64 |
| 3.4-4.1 | women | Standard | 50-59 | [10,15) | 0.65 | 0.20 | 0.37 | 0.12 | 435 | -40 | 138 | 271 | 65 |
| 3.4-4.1 | women | Standard | 50-59 | [15,20) | 0.76 | 0.24 | 0.41 | 0.14 | 485 | -37 | 192 | 264 | 67 |
| 3.4-4.1 | women | Standard | 50-59 | ≥20 | 0.89 | 0.30 | 0.45 | 0.16 | 618 | -21 | 311 | 259 | 69 |
| 3.4-4.1 | women | Standard | 50-59 | with CVD | 0.77 | 0.26 | 0.41 | 0.15 | 631 | -108 | 201 | 265 | 273 |
| 3.4-4.1 | women | Standard | 60-70 | <5 | 0.42 | 0.12 | 0.30 | 0.09 | 314 | -64 | 35 | 281 | 62 |
| 3.4-4.1 | women | Standard | 60-70 | [5,10) | 0.46 | 0.14 | 0.31 | 0.10 | 313 | -76 | 54 | 272 | 63 |
| 3.4-4.1 | women | Standard | 60-70 | [10,15) | 0.52 | 0.18 | 0.34 | 0.12 | 320 | -85 | 80 | 260 | 65 |
| 3.4-4.1 | women | Standard | 60-70 | [15,20) | 0.59 | 0.21 | 0.36 | 0.14 | 351 | -82 | 117 | 251 | 66 |
| 3.4-4.1 | women | Standard | 60-70 | ≥20 | 0.71 | 0.27 | 0.40 | 0.16 | 437 | -76 | 207 | 239 | 68 |
| 3.4-4.1 | women | Standard | 60-70 | with CVD | 0.74 | 0.29 | 0.37 | 0.15 | 546 | -175 | 229 | 240 | 252 |
| 3.4-4.1 | women | Higher intensity | 40-49 | <5 | 0.10 | 0.02 | 0.05 | 0.01 | 213 | 5 | 41 | 167 | -0 |
| 3.4-4.1 | women | Higher intensity | 40-49 | [5,10) | 0.11 | 0.03 | 0.04 | 0.01 | 254 | 14 | 84 | 157 | -1 |
| 3.4-4.1 | women | Higher intensity | 40-49 | ≥10 | 0.19 | 0.05 | 0.08 | 0.02 | 272 | 19 | 100 | 153 | -1 |
| 3.4-4.1 | women | Higher intensity | 40-49 | with CVD | 0.17 | 0.05 | 0.09 | 0.02 | 231 | 11 | 66 | 153 | 1 |
| 3.4-4.1 | women | Higher intensity | 50-59 | <5 | 0.10 | 0.02 | 0.06 | 0.01 | 194 | 1 | 37 | 157 | -0 |
| 3.4-4.1 | women | Higher intensity | 50-59 | [5,10) | 0.10 | 0.03 | 0.05 | 0.01 | 216 | 7 | 59 | 151 | -1 |
| 3.4-4.1 | women | Higher intensity | 50-59 | [10,15) | 0.12 | 0.04 | 0.05 | 0.01 | 240 | 14 | 83 | 144 | -1 |
| 3.4-4.1 | women | Higher intensity | 50-59 | [15,20) | 0.16 | 0.05 | 0.07 | 0.02 | 245 | 14 | 92 | 140 | -1 |
| 3.4-4.1 | women | Higher intensity | 50-59 | ≥20 | 0.20 | 0.06 | 0.10 | 0.03 | 228 | -0 | 92 | 138 | -1 |
| 3.4-4.1 | women | Higher intensity | 50-59 | with CVD | 0.17 | 0.05 | 0.08 | 0.03 | 233 | 9 | 83 | 141 | 1 |
| 3.4-4.1 | women | Higher intensity | 60-70 | <5 | 0.09 | 0.03 | 0.06 | 0.02 | 166 | -7 | 25 | 149 | -0 |
| 3.4-4.1 | women | Higher intensity | 60-70 | [5,10) | 0.09 | 0.03 | 0.05 | 0.02 | 178 | -5 | 40 | 144 | -1 |
| 3.4-4.1 | women | Higher intensity | 60-70 | [10,15) | 0.10 | 0.03 | 0.06 | 0.02 | 191 | -1 | 54 | 138 | -1 |
| 3.4-4.1 | women | Higher intensity | 60-70 | [15,20) | 0.12 | 0.04 | 0.06 | 0.02 | 205 | 2 | 70 | 133 | -1 |
| 3.4-4.1 | women | Higher intensity | 60-70 | ≥20 | 0.15 | 0.06 | 0.08 | 0.03 | 205 | -2 | 80 | 127 | -1 |
| 3.4-4.1 | women | Higher intensity | 60-70 | with CVD | 0.16 | 0.06 | 0.07 | 0.03 | 214 | -6 | 91 | 128 | 1 |
| ≥4.1 | men | Standard | 40-49 | <5 | 0.89 | 0.22 | 0.65 | 0.16 | 228 | -183 | 37 | 308 | 66 |
| ≥4.1 | men | Standard | 40-49 | [5,10) | 1.05 | 0.29 | 0.74 | 0.21 | 190 | -240 | 66 | 296 | 69 |
| ≥4.1 | men | Standard | 40-49 | ≥10 | 1.64 | 0.54 | 1.09 | 0.37 | 353 | -267 | 266 | 279 | 74 |
| ≥4.1 | men | Standard | 40-49 | with CVD | 1.45 | 0.58 | 0.78 | 0.31 | 717 | -319 | 507 | 260 | 269 |
| ≥4.1 | men | Standard | 50-59 | <5 | 0.73 | 0.20 | 0.55 | 0.16 | 254 | -132 | 30 | 292 | 65 |
| ≥4.1 | men | Standard | 50-59 | [5,10) | 0.82 | 0.25 | 0.60 | 0.19 | 196 | -195 | 43 | 281 | 67 |
| ≥4.1 | men | Standard | 50-59 | [10,15) | 0.94 | 0.31 | 0.67 | 0.23 | 178 | -232 | 70 | 271 | 69 |
| ≥4.1 | men | Standard | 50-59 | [15,20) | 1.09 | 0.37 | 0.76 | 0.27 | 188 | -256 | 111 | 262 | 71 |
| ≥4.1 | men | Standard | 50-59 | ≥20 | 1.65 | 0.65 | 1.09 | 0.44 | 491 | -193 | 360 | 248 | 76 |
| ≥4.1 | men | Standard | 50-59 | with CVD | 1.19 | 0.54 | 0.60 | 0.27 | 381 | -547 | 453 | 231 | 244 |
| ≥4.1 | men | Standard | 60-70 | [5,10) | 0.67 | 0.23 | 0.50 | 0.18 | 265 | -105 | 44 | 261 | 65 |
| ≥4.1 | men | Standard | 60-70 | [10,15) | 0.75 | 0.27 | 0.55 | 0.20 | 199 | -173 | 52 | 254 | 67 |
| ≥4.1 | men | Standard | 60-70 | [15,20) | 0.84 | 0.32 | 0.60 | 0.23 | 198 | -191 | 74 | 246 | 68 |
| ≥4.1 | men | Standard | 60-70 | ≥20 | 1.23 | 0.53 | 0.83 | 0.37 | 409 | -130 | 239 | 227 | 73 |
| ≥4.1 | men | Standard | 60-70 | with CVD | 0.92 | 0.46 | 0.46 | 0.23 | 66 | -697 | 342 | 202 | 219 |
| ≥4.1 | men | Higher intensity | 40-49 | <5 | 0.17 | 0.04 | 0.11 | 0.02 | 184 | -26 | 48 | 163 | -1 |
| ≥4.1 | men | Higher intensity | 40-49 | [5,10) | 0.20 | 0.05 | 0.12 | 0.03 | 184 | -37 | 65 | 157 | -1 |
| ≥4.1 | men | Higher intensity | 40-49 | ≥10 | 0.33 | 0.10 | 0.20 | 0.06 | 211 | -47 | 111 | 149 | -2 |
| ≥4.1 | men | Higher intensity | 40-49 | with CVD | 0.31 | 0.12 | 0.15 | 0.06 | 279 | -27 | 165 | 140 | 2 |
| ≥4.1 | men | Higher intensity | 50-59 | <5 | 0.15 | 0.04 | 0.10 | 0.03 | 170 | -16 | 32 | 155 | -1 |
| ≥4.1 | men | Higher intensity | 50-59 | [5,10) | 0.16 | 0.05 | 0.10 | 0.03 | 163 | -29 | 44 | 149 | -1 |
| ≥4.1 | men | Higher intensity | 50-59 | [10,15) | 0.18 | 0.06 | 0.11 | 0.04 | 164 | -37 | 58 | 144 | -1 |
| ≥4.1 | men | Higher intensity | 50-59 | [15,20) | 0.20 | 0.07 | 0.13 | 0.04 | 164 | -45 | 71 | 140 | -2 |
| ≥4.1 | men | Higher intensity | 50-59 | ≥20 | 0.33 | 0.12 | 0.20 | 0.08 | 208 | -42 | 119 | 133 | -2 |
| ≥4.1 | men | Higher intensity | 50-59 | with CVD | 0.25 | 0.11 | 0.11 | 0.05 | 197 | -75 | 146 | 124 | 1 |
| ≥4.1 | men | Higher intensity | 60-70 | [5,10) | 0.14 | 0.05 | 0.10 | 0.03 | 155 | -13 | 30 | 139 | -1 |
| ≥4.1 | men | Higher intensity | 60-70 | [10,15) | 0.15 | 0.05 | 0.10 | 0.04 | 143 | -29 | 38 | 135 | -1 |
| ≥4.1 | men | Higher intensity | 60-70 | [15,20) | 0.16 | 0.06 | 0.10 | 0.04 | 141 | -35 | 46 | 131 | -1 |
| ≥4.1 | men | Higher intensity | 60-70 | ≥20 | 0.24 | 0.10 | 0.15 | 0.06 | 175 | -30 | 85 | 122 | -2 |
| ≥4.1 | men | Higher intensity | 60-70 | with CVD | 0.19 | 0.10 | 0.08 | 0.04 | 110 | -115 | 115 | 109 | 1 |
| ≥4.1 | women | Standard | 40-49 | <5 | 0.76 | 0.16 | 0.49 | 0.11 | 384 | -64 | 71 | 316 | 62 |
| ≥4.1 | women | Standard | 40-49 | [5,10) | 1.08 | 0.28 | 0.61 | 0.16 | 445 | -83 | 164 | 299 | 65 |
| ≥4.1 | women | Standard | 40-49 | ≥10 | 1.71 | 0.52 | 0.89 | 0.28 | 716 | -67 | 427 | 288 | 69 |
| ≥4.1 | women | Standard | 40-49 | with CVD | 1.18 | 0.39 | 0.59 | 0.20 | 881 | -52 | 372 | 277 | 284 |
| ≥4.1 | women | Standard | 50-59 | <5 | 0.70 | 0.18 | 0.48 | 0.13 | 346 | -78 | 62 | 299 | 63 |
| ≥4.1 | women | Standard | 50-59 | [5,10) | 0.86 | 0.24 | 0.54 | 0.16 | 355 | -104 | 107 | 288 | 64 |
| ≥4.1 | women | Standard | 50-59 | [10,15) | 1.10 | 0.33 | 0.65 | 0.20 | 420 | -108 | 184 | 277 | 66 |
| ≥4.1 | women | Standard | 50-59 | [15,20) | 1.37 | 0.44 | 0.77 | 0.26 | 507 | -108 | 277 | 269 | 68 |
| ≥4.1 | women | Standard | 50-59 | ≥20 | 1.85 | 0.66 | 0.99 | 0.37 | 825 | -54 | 547 | 260 | 72 |
| ≥4.1 | women | Standard | 50-59 | with CVD | 1.09 | 0.40 | 0.52 | 0.20 | 659 | -228 | 359 | 259 | 268 |
| ≥4.1 | women | Standard | 60-70 | <5 | 0.59 | 0.17 | 0.43 | 0.13 | 303 | -87 | 44 | 284 | 62 |
| ≥4.1 | women | Standard | 60-70 | [5,10) | 0.70 | 0.22 | 0.47 | 0.16 | 297 | -115 | 73 | 276 | 64 |
| ≥4.1 | women | Standard | 60-70 | [10,15) | 0.81 | 0.27 | 0.53 | 0.18 | 297 | -142 | 108 | 266 | 65 |
| ≥4.1 | women | Standard | 60-70 | [15,20) | 0.97 | 0.34 | 0.60 | 0.22 | 330 | -152 | 158 | 258 | 67 |
| ≥4.1 | women | Standard | 60-70 | ≥20 | 1.36 | 0.52 | 0.78 | 0.31 | 525 | -122 | 331 | 246 | 69 |
| ≥4.1 | women | Standard | 60-70 | with CVD | 0.96 | 0.40 | 0.45 | 0.19 | 336 | -456 | 303 | 238 | 250 |
| ≥4.1 | women | Higher intensity | 40-49 | <5 | 0.15 | 0.03 | 0.08 | 0.02 | 226 | 4 | 55 | 167 | -1 |
| ≥4.1 | women | Higher intensity | 40-49 | [5,10) | 0.21 | 0.05 | 0.09 | 0.02 | 267 | 11 | 99 | 159 | -1 |
| ≥4.1 | women | Higher intensity | 40-49 | ≥10 | 0.36 | 0.10 | 0.17 | 0.05 | 291 | 8 | 131 | 154 | -2 |
| ≥4.1 | women | Higher intensity | 40-49 | with CVD | 0.24 | 0.08 | 0.11 | 0.04 | 309 | 33 | 127 | 148 | 1 |
| ≥4.1 | women | Higher intensity | 50-59 | <5 | 0.14 | 0.03 | 0.08 | 0.02 | 200 | -2 | 44 | 158 | -1 |
| ≥4.1 | women | Higher intensity | 50-59 | [5,10) | 0.17 | 0.04 | 0.09 | 0.02 | 222 | 0 | 69 | 153 | -1 |
| ≥4.1 | women | Higher intensity | 50-59 | [10,15) | 0.21 | 0.06 | 0.11 | 0.03 | 255 | 7 | 101 | 148 | -1 |
| ≥4.1 | women | Higher intensity | 50-59 | [15,20) | 0.27 | 0.08 | 0.13 | 0.04 | 277 | 12 | 123 | 144 | -1 |
| ≥4.1 | women | Higher intensity | 50-59 | ≥20 | 0.40 | 0.13 | 0.20 | 0.07 | 293 | 3 | 151 | 140 | -2 |
| ≥4.1 | women | Higher intensity | 50-59 | with CVD | 0.22 | 0.08 | 0.09 | 0.03 | 260 | -3 | 124 | 138 | 1 |
| ≥4.1 | women | Higher intensity | 60-70 | <5 | 0.13 | 0.04 | 0.08 | 0.02 | 172 | -6 | 28 | 150 | -1 |
| ≥4.1 | women | Higher intensity | 60-70 | [5,10) | 0.14 | 0.04 | 0.09 | 0.03 | 182 | -9 | 46 | 146 | -1 |
| ≥4.1 | women | Higher intensity | 60-70 | [10,15) | 0.16 | 0.05 | 0.09 | 0.03 | 196 | -9 | 64 | 141 | -1 |
| ≥4.1 | women | Higher intensity | 60-70 | [15,20) | 0.19 | 0.07 | 0.10 | 0.04 | 211 | -7 | 82 | 137 | -1 |
| ≥4.1 | women | Higher intensity | 60-70 | ≥20 | 0.28 | 0.10 | 0.15 | 0.06 | 240 | -6 | 116 | 132 | -1 |
| ≥4.1 | women | Higher intensity | 60-70 | with CVD | 0.19 | 0.08 | 0.08 | 0.03 | 182 | -56 | 109 | 127 | 1 |

CVD, cardiovascular disease; LDL-C, low density lipoprotein cholesterol; QALY, Quality Adjusted Life Year

## Supplementary Figure S3 Probability of statin therapy being cost-effective at different thresholds of cost-effectiveness (£/QALY)

1. **People without history of cardiovascular disease**

A1. Men 40-49 years old


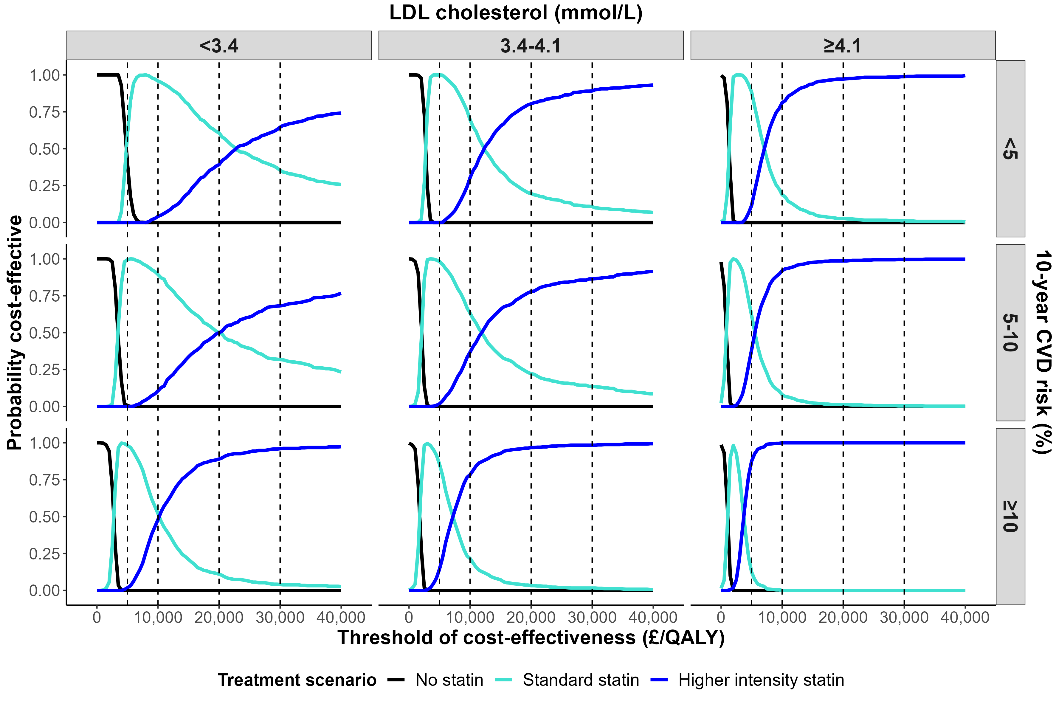


A2. Men 50-59 years old


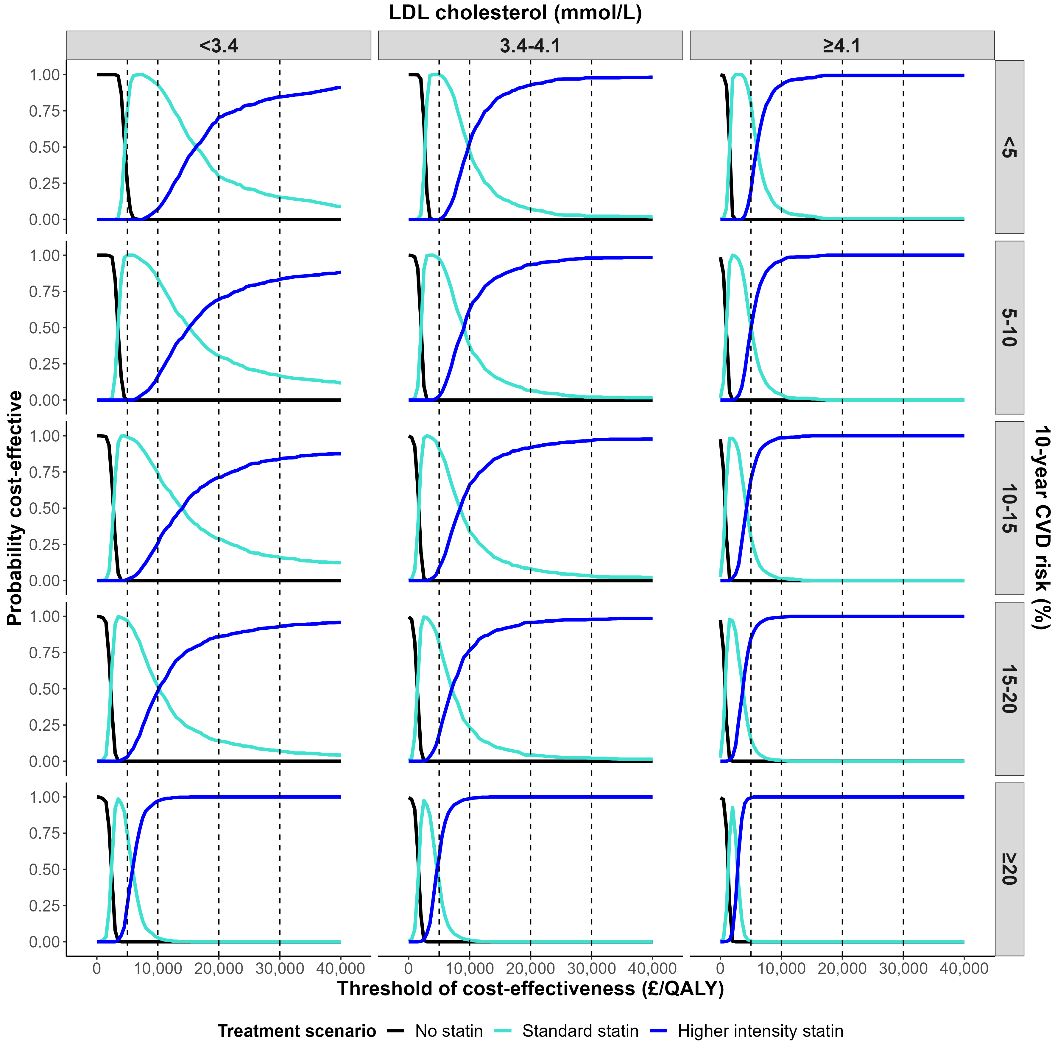


A3. Men 60-70 years old


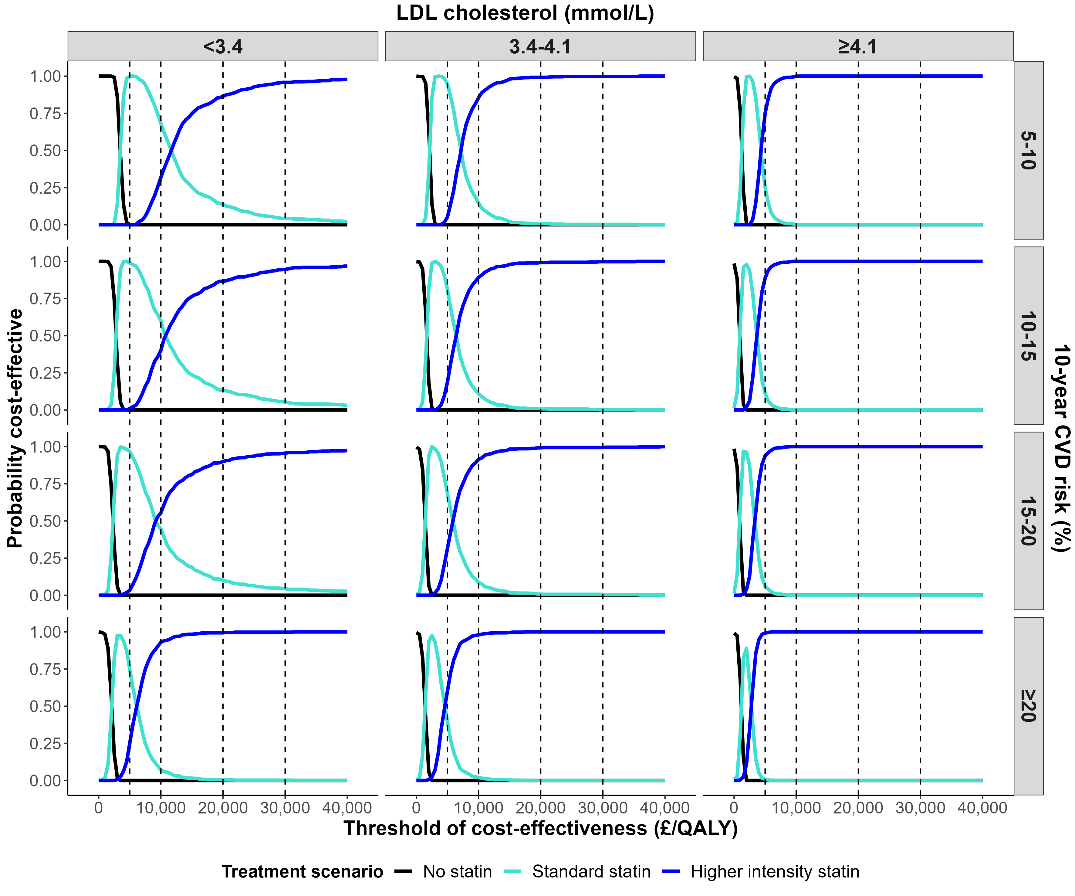


A4. Women 40-49 years old


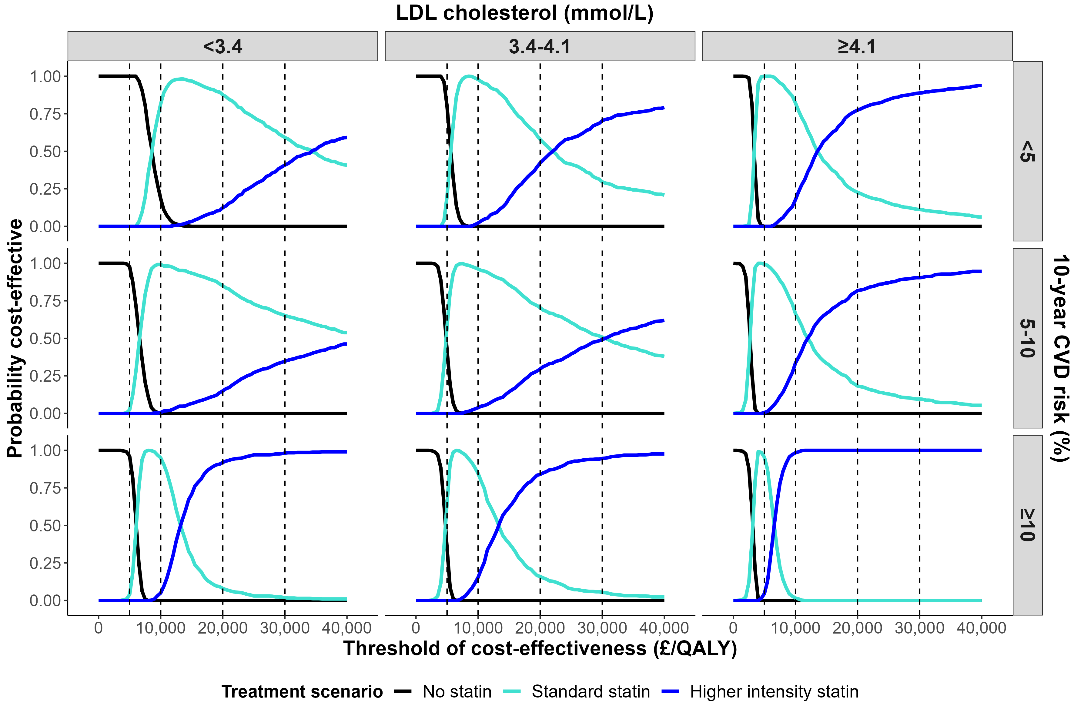


A5. Women 50-59 years old


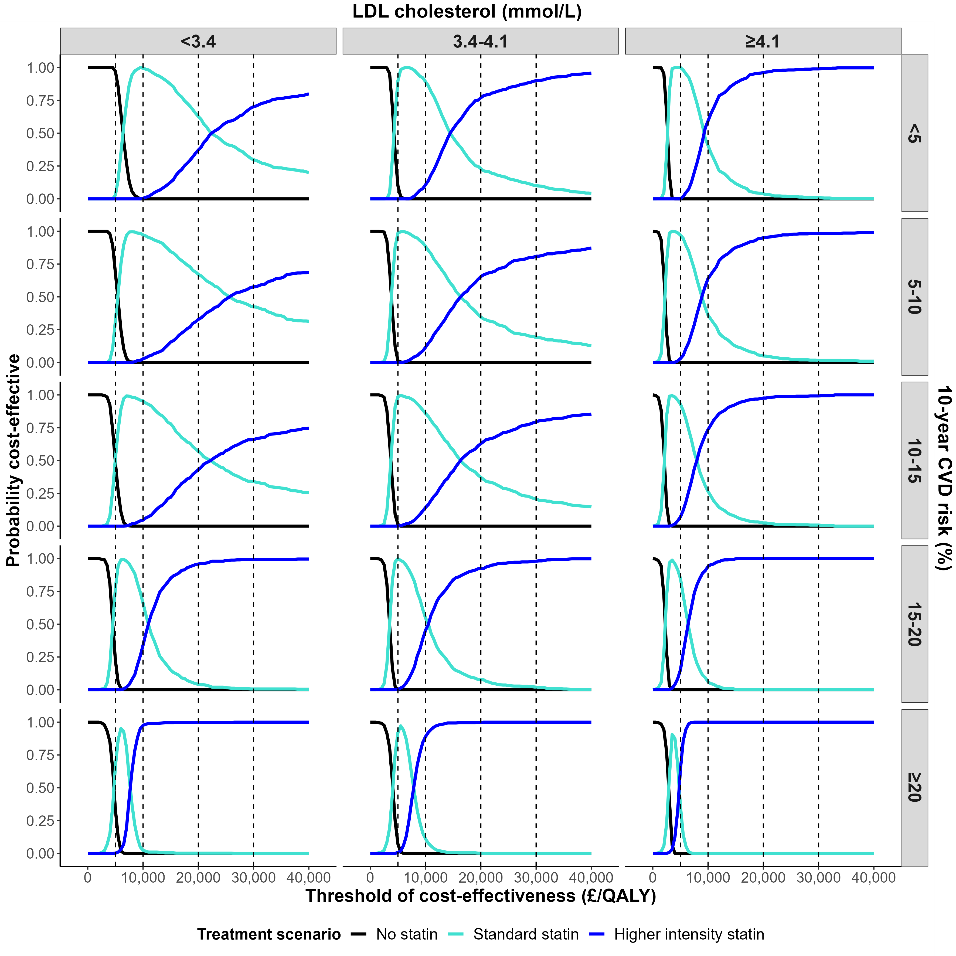


A6. Women 60-70 years old


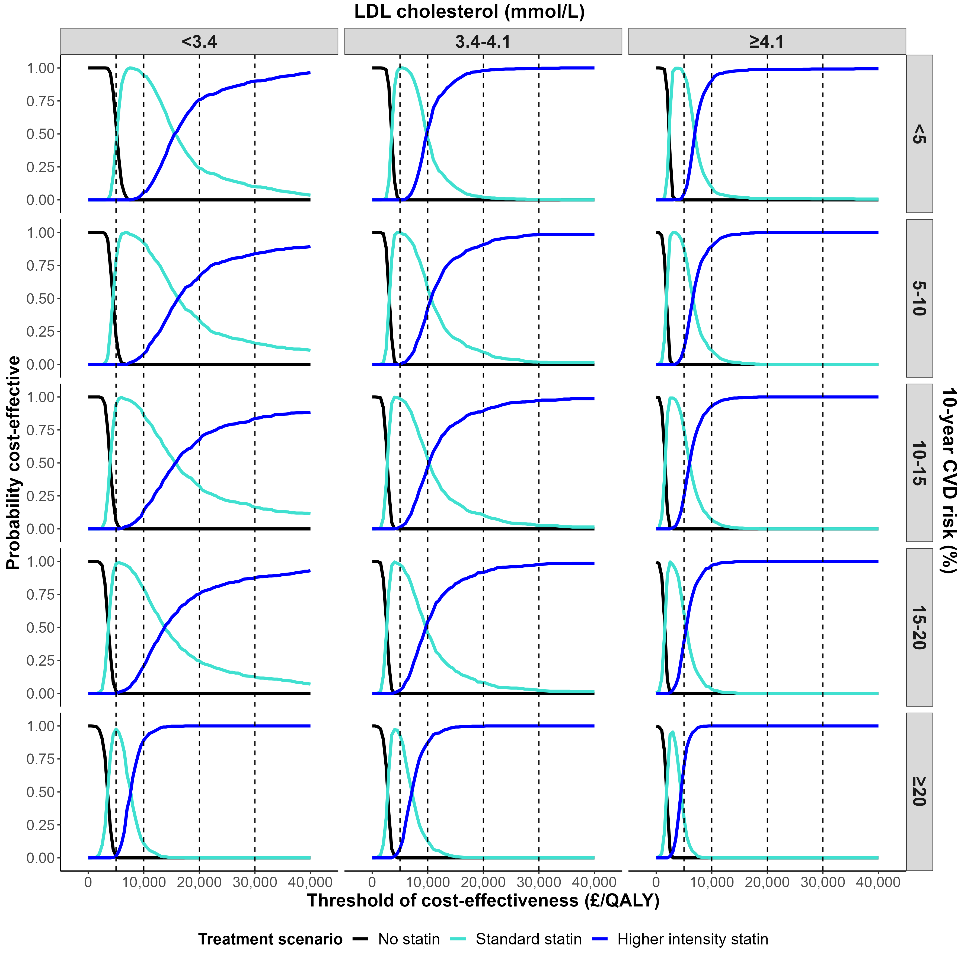


**B. People with history of CVD**

B1. Men


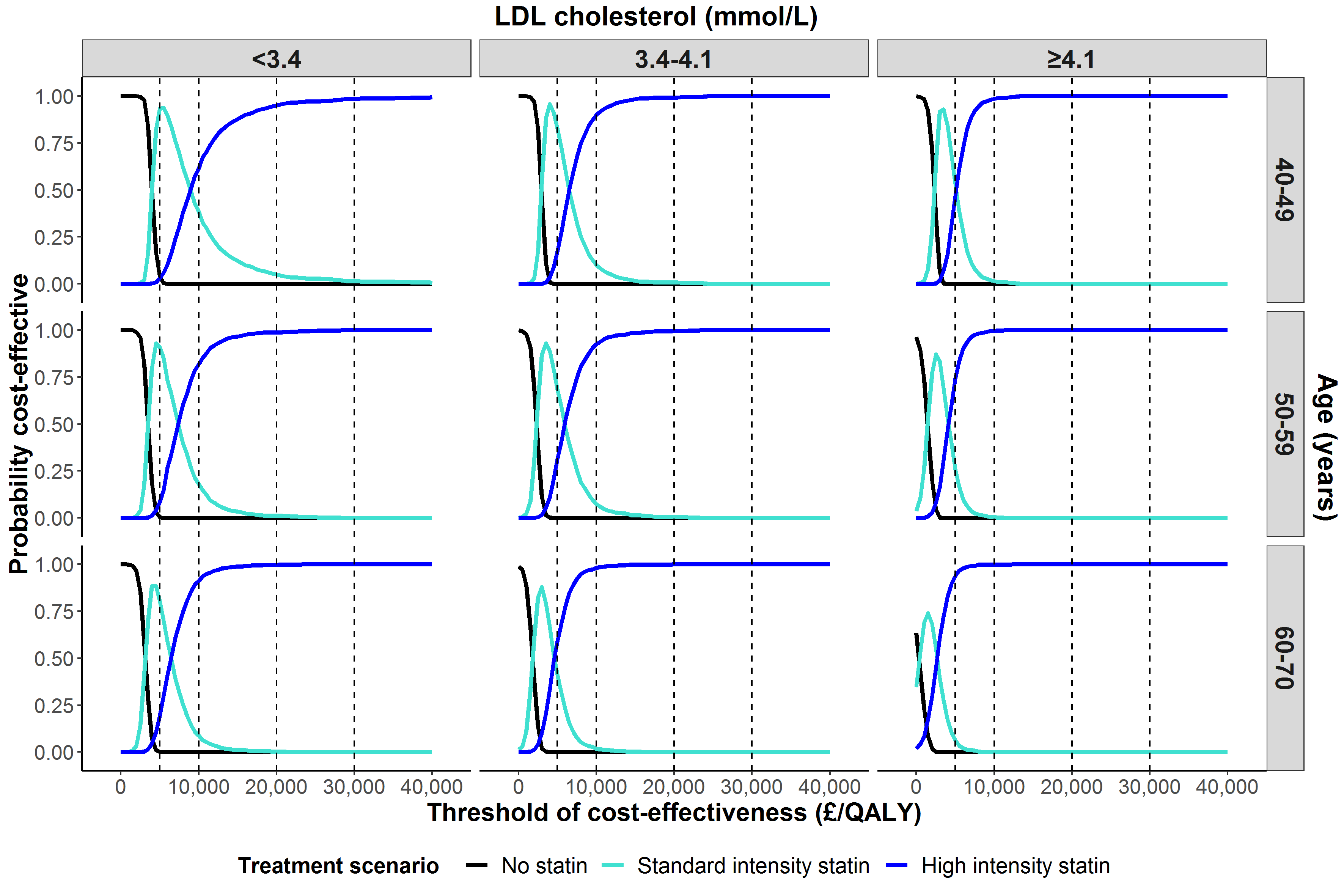


B2. Women


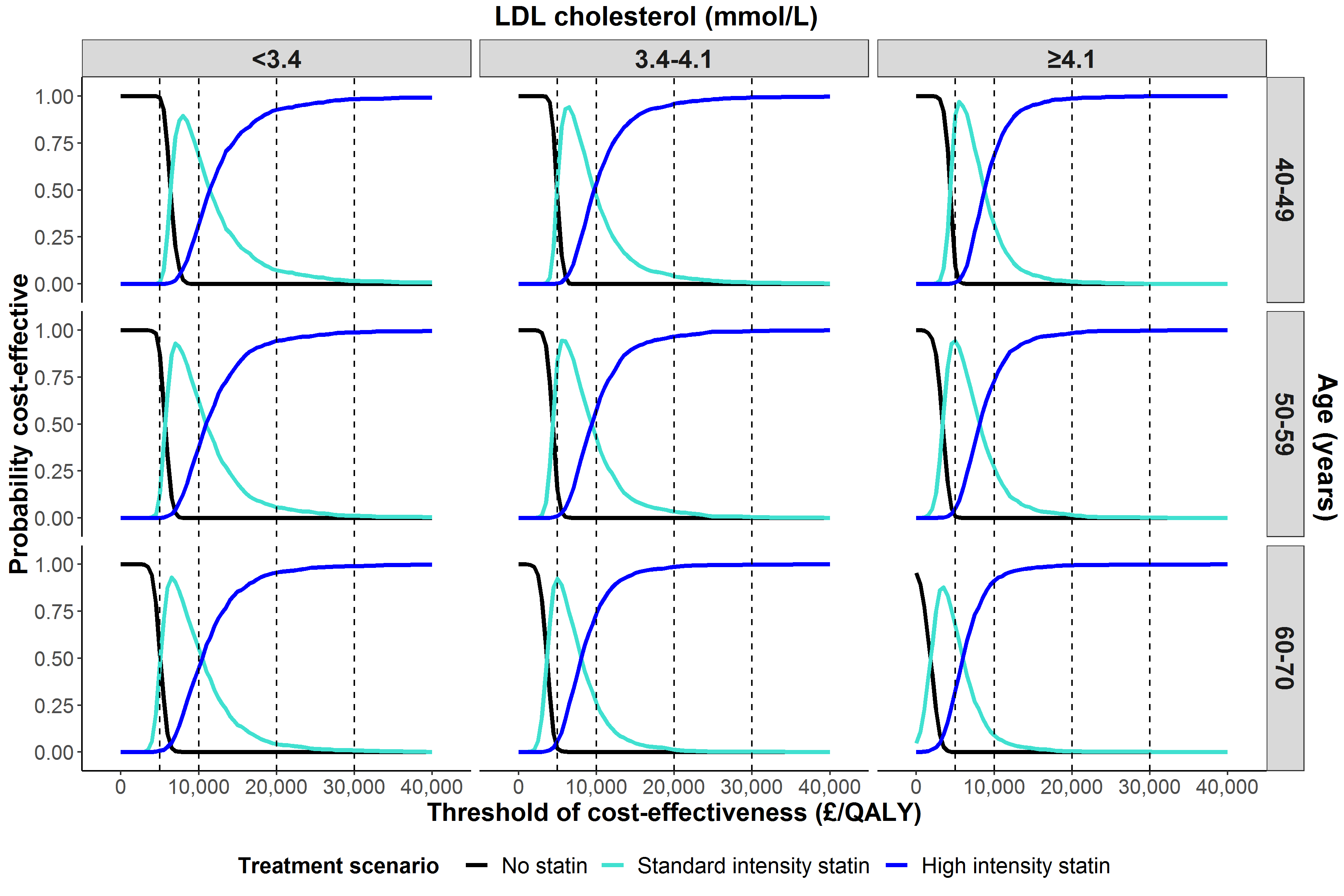


LDL, low density lipoprotein; CVD, cardiovascular disease; QALY, quality-adjusted life year.

## Supplementary Table S4 Impact of excess new diabetes due to statin therapy on QALYs gained

1. **In people without history of cardiovascular disease and without diabetes at baseline**

| **Sex, CVD risk** | **Men, high** | **Men, moderate** | **Women, high** | **Women, moderate** |
| --- | --- | --- | --- | --- |
| **Age (years)** | **60-70** | **40-49** | **60-70** | **40-49** |
| **10-year CVD (%)** | **15-20** | **5-10** | **15-20** | **5-10** |
| **LDL cholesterol (mmol/L)** | **≥4.1** | **<3.4** | **≥4.1** | **<3.4** |
| **QALYs gained (undiscounted) with Standard statin vs No statin** | | | | |
| Base-case (with excess diabetes) | 0.60 | 0.31 | 0.60 | 0.24 |
| Without excess diabetes | 0.62 | 0.34 | 0.63 | 0.27 |
| **QALYs lost due to excess diabetes with statin** | **-0.02** | **-0.03** | **-0.02** | **-0.03** |
| **QALYs gained (undiscounted) with Higher intensity vs Standard statin** | | | | |
| Base-case (with excess diabetes) | 0.10 | 0.04 | 0.10 | 0.02 |
| Without excess diabetes | 0.14 | 0.09 | 0.14 | 0.07 |
| **QALYs lost due to excess diabetes with statin** | **-0.03** | **-0.05** | **-0.03** | **-0.05** |

1. **In people with history of cardiovascular disease and without diabetes at baseline**

| **Sex, CVD risk** | **Men, very high** | **Men, high** | **Women, very high** | **Women, high** |
| --- | --- | --- | --- | --- |
| **Age (years)** | **60-70** | **40-49** | **60-70** | **40-49** |
| **LDL cholesterol (mmol/L)** | **≥4.1** | **<3.4** | **≥4.1** | **<3.4** |
| **QALYs gained (undiscounted) with Standard statin vs No statin** | | | | |
| Base-case (with excess diabetes) | 0.46 | 0.38 | 0.46 | 0.35 |
| Without excess diabetes | 0.48 | 0.41 | 0.48 | 0.37 |
| **QALYs lost due to excess diabetes with statin** | **-0.02** | **-0.03** | **-0.02** | **-0.02** |
| **QALYs gained (undiscounted) with Higher intensity vs Standard statin** | | | | |
| Base-case (with excess diabetes) | 0.08 | 0.07 | 0.07 | 0.07 |
| Without excess diabetes | 0.11 | 0.11 | 0.11 | 0.10 |
| **QALYs lost due to excess diabetes with statin** | **-0.03** | **-0.04** | **-0.04** | **-0.03** |

LDL, low density lipoprotein; QALY, quality-adjusted life year.

## Supplementary Figure S4 Sensitivity analyses of cost-effectiveness of standard statin versus no statin therapy

(a) People without history of cardiovascular disease


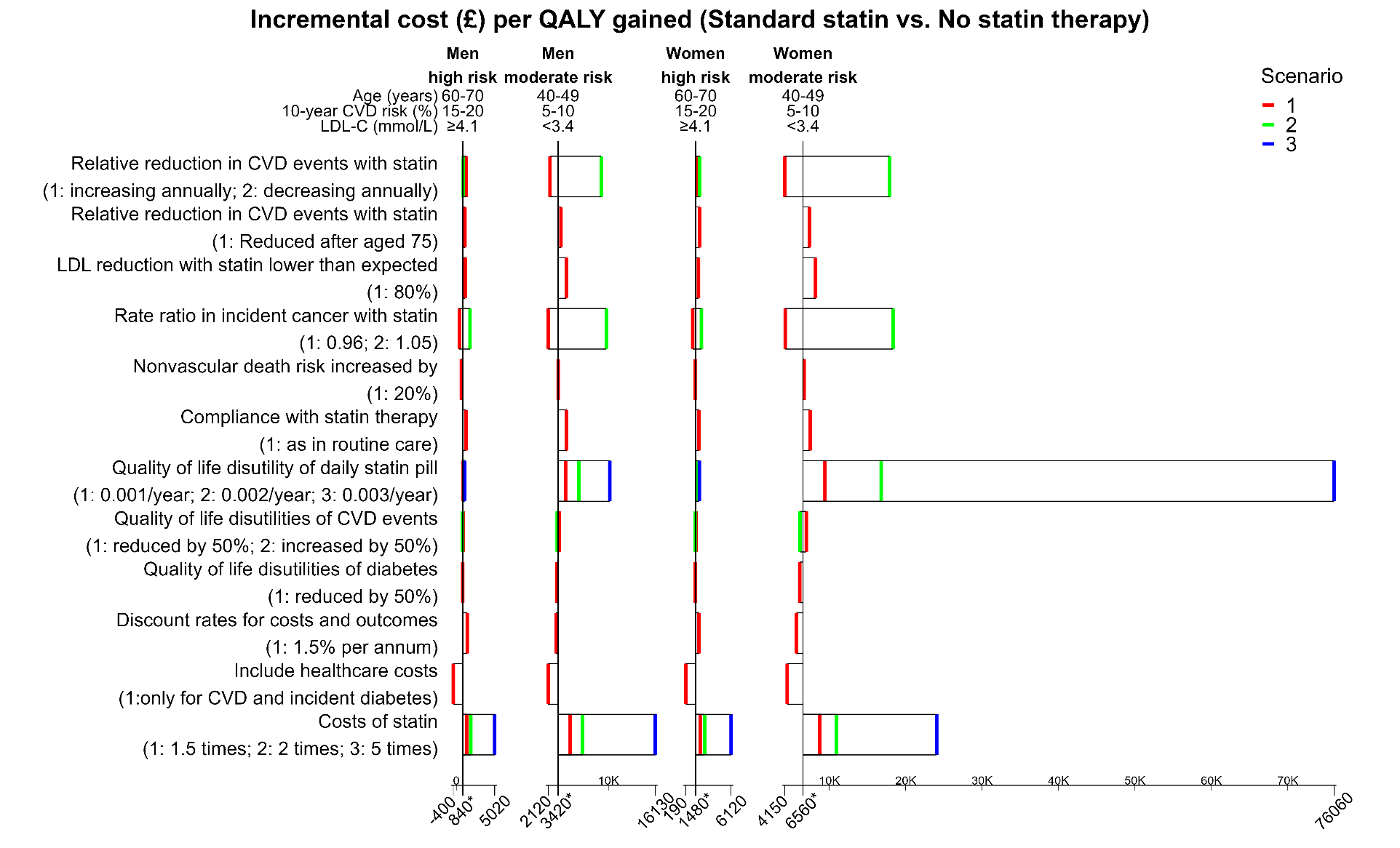


*Incremental cost per QALY in base-case cost-effectiveness analysis.

CVD, cardiovascular disease; LDL-C, low density lipoprotein cholesterol; QALY, Quality-Adjusted Life Year.

(b) people with history of cardiovascular disease


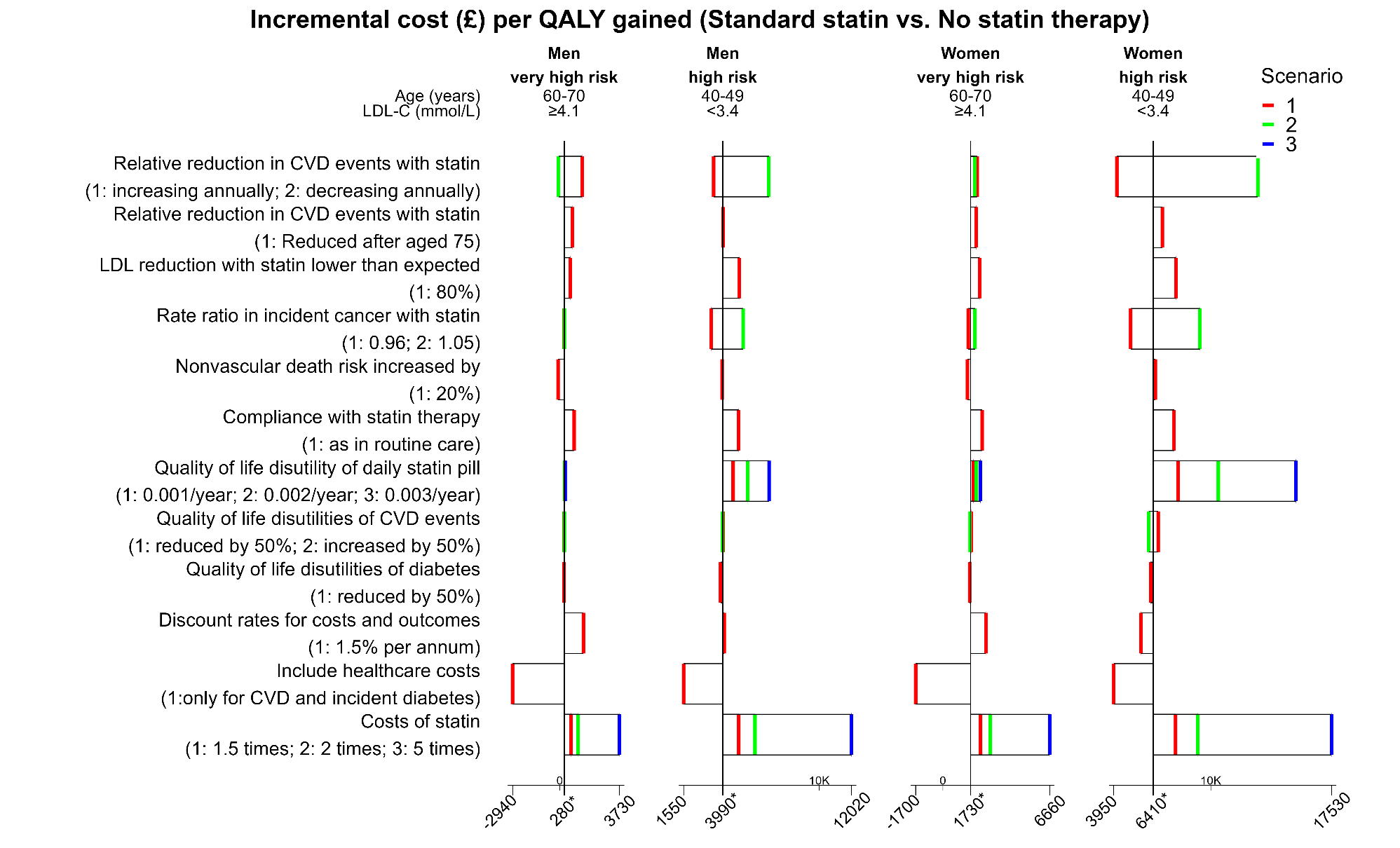


*Incremental cost per QALY in base-case cost-effectiveness analysis.

CVD, cardiovascular disease; LDL-C, low density lipoprotein cholesterol; QALY, Quality-Adjusted Life Year.

## Supplementary Figure S5 Sensitivity analyses of cost-effectiveness of higher intensity versus standard statin therapy

**(a) People without history of cardiovascular disease**


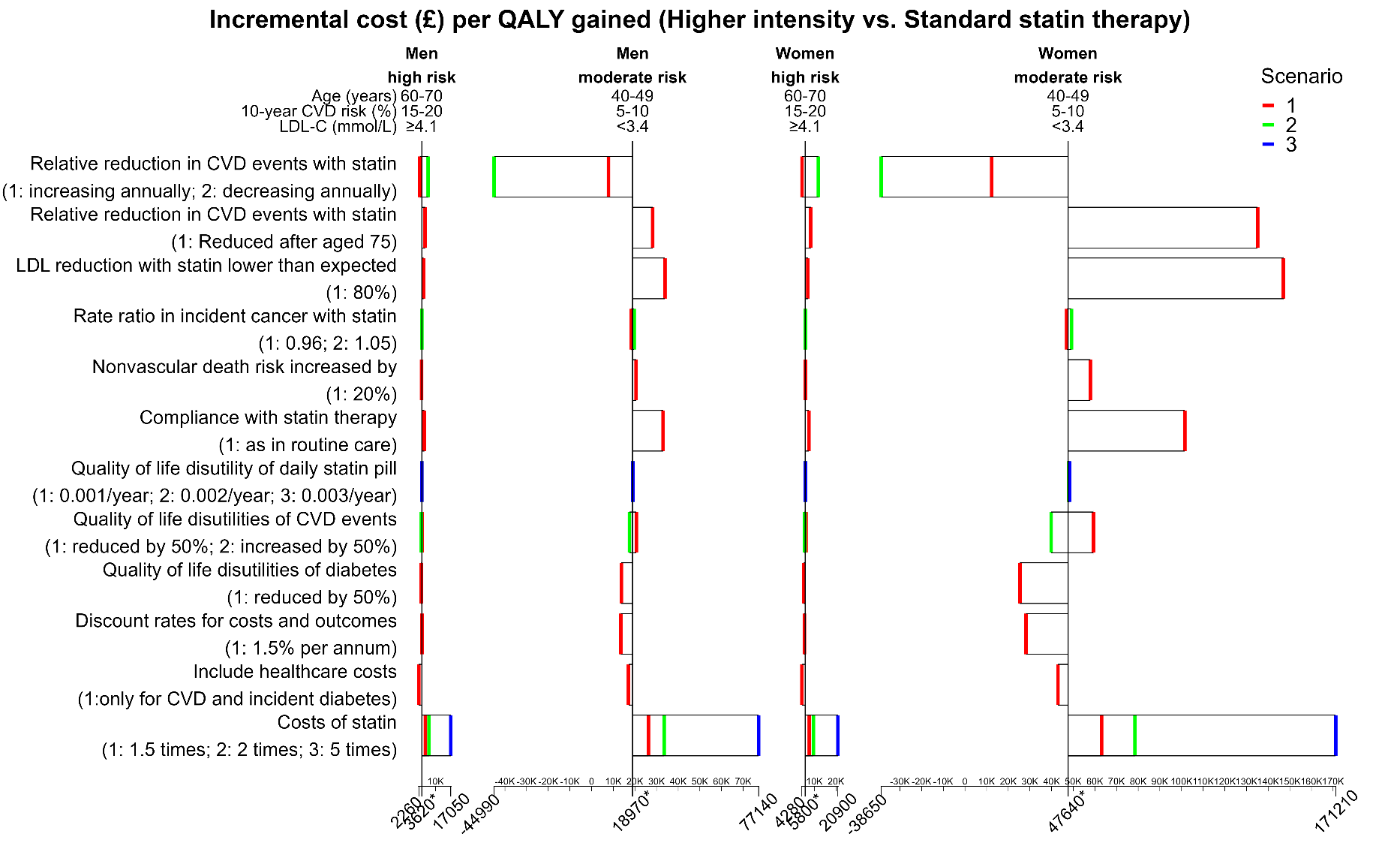


*Incremental cost per QALY in base-case cost-effectiveness analysis. CVD, cardiovascular disease; LDL-C, low density lipoprotein cholesterol; QALY, Quality-Adjusted Life Year. In the scenario analysis with decreasing relative reductions in CVD events with statin therapy over time, negative incremental discounted QALYs in patient categories with LDL<3.4mmol/L and negative incremental cost per QALY are projected.

**(b) People with history of cardiovascular disease**

**
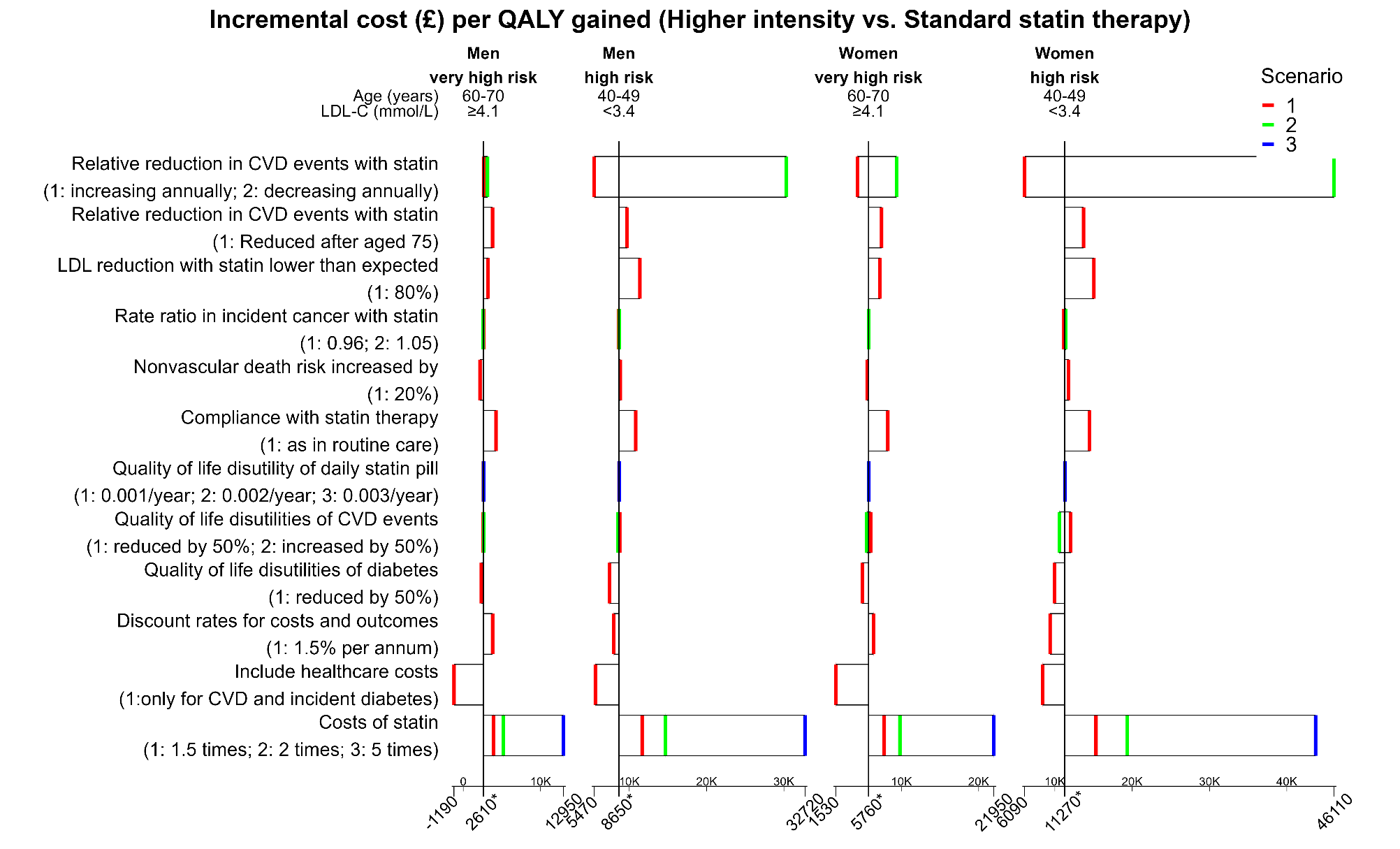
**

*Incremental cost per QALY in base-case cost-effectiveness analysis. CVD, cardiovascular disease; LDL-C, low density lipoprotein cholesterol; QALY, Quality-Adjusted Life Year.

## Supplementary Figure S6 QALYs gained per person and cost-effectiveness of long-term statin therapy in categories by sex, age, pre-treatment LDL cholesterol level and cardiovascular risk: comparison of basecase and scenario analysis with variability around the proportional reduction in LDL-C with statin therapy

1. **Standard statin therapy**


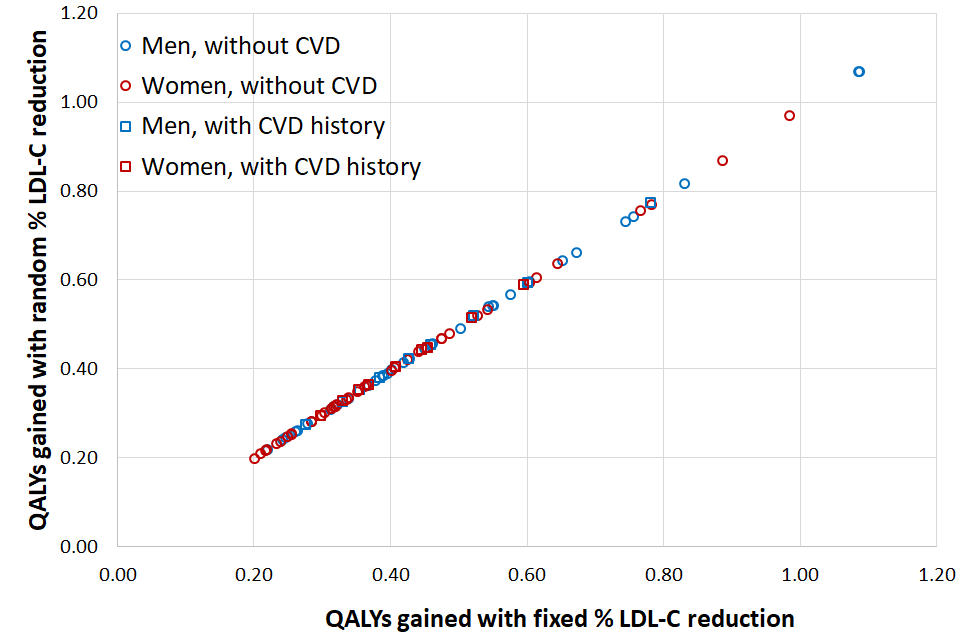

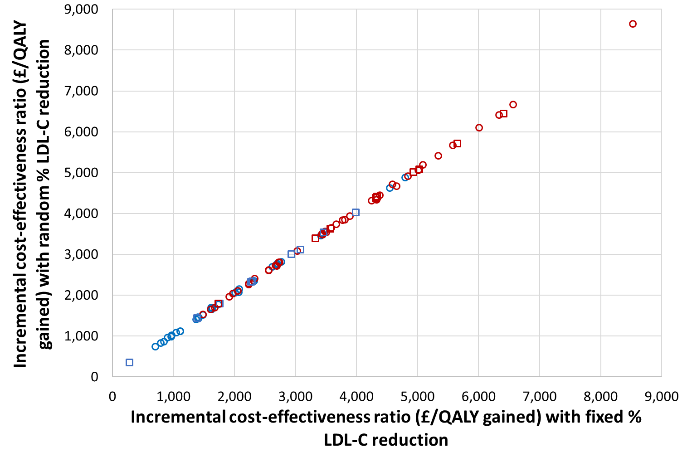


**(b) Higher intensity statin therapy**


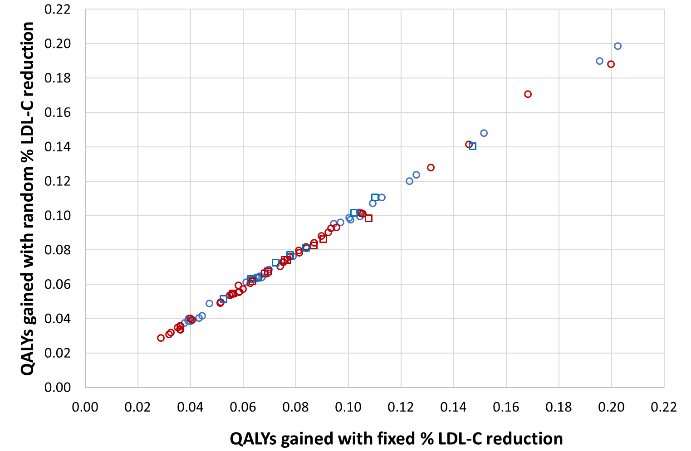

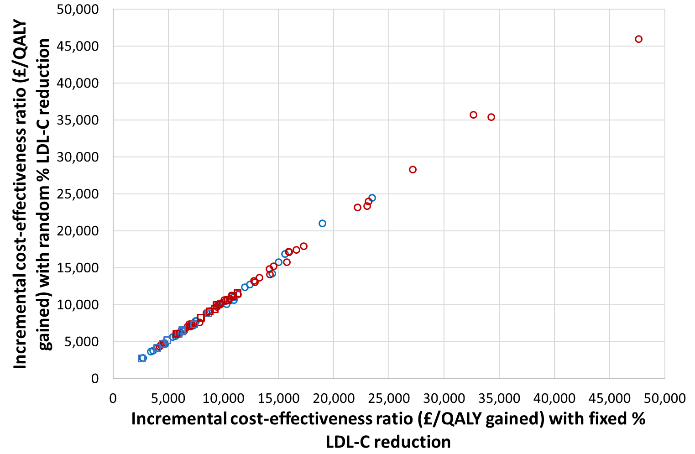


Results presented in participant categories by sex (men, women), age (40-49, 50-59, 60-70 years), pre-treatment LDL-C level (<3.4, 3.4-4.1, ≥4.1mmol/L) and cardiovascular risk (10-year cardiovascular risk <5%, 5%-10%, 10%-15%, 15%-20%, ≥20%) or history of cardiovascular disease history at statin initiation.

% LDL-C reduction at 43% for standard statin and 55% for higher intensity statin therapy (basecase) and sampled (as part of the 500 microsimulations) from normal distributions with mean 43% (standard deviation 14.5%) for standard statin and mean 55% (standard deviation 17.3%) for higher intensity statin therapy.

## Supplementary Table S5 Sensitivity analyses for cost-effectiveness (£/QALY) of statin therapy

1. **People without history of cardiovascular disease: Standard statin vs. No statin therapy**

| **Sex, cardiovascular risk** | **Men, high** | **Men, moderate** | **Women, high** | **Women, moderate** | **Men, low risk** | **Men, low risk** | **Women, low risk** | **Women, low risk** |
| --- | --- | --- | --- | --- | --- | --- | --- | --- |
| **Age (years)** | **60-70** | **40-49** | **60-70** | **40-49** | **50-59** | **40-49** | **50-59** | **40-49** |
| **10-year cardiovascular risk (%)** | **15-20** | **5-10** | **15-20** | **5-10** | **<5** | **<5** | **<5** | **<5** |
| **LDL cholesterol (mmol/L)** | **≥4.1** | **<3.4** | **≥4.1** | **<3.4** | **<3.4** | **<3.4** | **<3.4** | **<3.4** |
| **Base-case** | £840 | £3,420 | £1,480 | £6,560 | £4,540 | £4,790 | £6,340 | £8,530 |
| Relative reduction in CVD events with statin therapy increasing annually | £1,300 | £2,360 | £1,640 | £4,150 | £2,550 | £2,520 | £2,970 | £3,620 |
| Relative reduction in CVD events with statin therapy decreasing annually | £920 | £9,050 | £2,010 | £17,880 | £11,960 | £16,520 | £18,860 | £34,650 |
| Relative reduction in CVD events with statin therapy reduced in elderly | £1,120 | £3,790 | £1,990 | £7,410 | £5,690 | £5,710 | £8,440 | £10,960 |
| LDL reduction with statin 80% from expected | £1,160 | £4,500 | £1,850 | £8,200 | £5,820 | £6,220 | £8,030 | £10,800 |
| Rate ratio in incident cancer with statin therapy of 0.96 | £390 | £2,120 | £1,090 | £4,230 | £2,160 | £2,620 | £3,240 | £4,320 |
| Rate ratio in incident cancer with statin therapy of 1.05 | £1,790 | £9,720 | £2,230 | £18,340 | £242,270 | £31,410 | £131,210 | -£923,710* |
| Risk of nonvascular death increased by 20% | £650 | £3,420 | £1,400 | £6,700 | £4,650 | £4,930 | £6,630 | £9,020 |
| Compliance with statin therapy as in routine care | £1,260 | £4,520 | £1,920 | £7,480 | £5,410 | £5,760 | £7,540 | £10,210 |
| With quality of life disutility of daily statin pill of 0.001/year | £910 | £4,390 | £1,610 | £9,440 | £6,310 | £6,920 | £9,910 | £16,270 |
| With quality of life disutility of daily statin pill of 0.002/year | £980 | £6,130 | £1,770 | £16,790 | £10,360 | £12,400 | £22,730 | £178,620 |
| With quality of life disutility of daily statin pill of 0.003/year | £1,080 | £10,180 | £1,960 | £76,060 | £28,760 | £60,050 | -£77,460* | -£19,900* |
| Quality of life disutilities of CVD events reduced by 50% | £870 | £3,550 | £1,540 | £6,990 | £4,740 | £4,960 | £6,760 | £9,030 |
| Quality of life disutilities of CVD events increased by 50% | £820 | £3,300 | £1,430 | £6,190 | £4,360 | £4,630 | £5,960 | £8,080 |
| Quality of life disutilities of diabetes reduced by 50% | £830 | £3,280 | £1,450 | £6,130 | £4,440 | £4,640 | £6,170 | £8,250 |
| Discount rates for costs and outcomes at 1.5% per annum | £1,420 | £3,140 | £1,910 | £5,700 | £3,700 | £3,740 | £4,790 | £5,960 |
| Include only healthcare costs for CVD and incident diabetes | [-£400] | £2,120 | £190 | £4,490 | £3,410 | £3,710 | £5,150 | £7,230 |
| Cost of statin x1.5 | £1,360 | £5,010 | £2,060 | £8,750 | £6,560 | £6,990 | £8,920 | £11,940 |
| Cost of statin x2 | £1,890 | £6,590 | £2,640 | £10,930 | £8,570 | £9,200 | £11,510 | £15,360 |
| Cost of statin x5 | £5,020 | £16,130 | £6,120 | £24,040 | £20,650 | £22,400 | £27,040 | £35,860 |
| With variability around the % reduction in LDL-C with statin therapy | £870 | £3,460 | £1,530 | £6,670 | £4,630 | £4,880 | £6,420 | £8,640 |

CVD, cardiovascular disease. Negative values in brackets represent cost savings. *Negative values indicate result due to decrease in QALYs.

1. **People without history of cardiovascular disease: Higher intensity vs standard statin therapy**

| **Sex, cardiovascular risk** | | **Men, high** | | **Men, moderate** | | **Women, high** | | **Women, moderate** | | **Men, low risk** | | **Men, low risk** | | **Women, low risk** | | **Women, low risk** |  |
| --- | --- | --- | --- | --- | --- | --- | --- | --- | --- | --- | --- | --- | --- | --- | --- | --- | --- |
| **Age (years)** | | **60-70** | | **40-49** | | **60-70** | | **40-49** | | **50-59** | | **40-49** | | **50-59** | | **40-49** |  |
| **10-year cardiovascular risk (%)** | | **15-20** | | **5-10** | | **15-20** | | **5-10** | | **<5** | | **<5** | | **<5** | | **<5** |  |
| **LDL cholesterol (mmol/L)** | | **≥4.1** | | **<3.4** | | **≥4.1** | | **<3.4** | | **<3.4** | | **<3.4** | | **<3.4** | | **<3.4** |  |
| **Base-case** | | £3,620 | | £18,970 | | £5,800 | | £47,640 | | £15,570 | | £23,490 | | £23,050 | | £34,290 |  |
| Relative reduction in CVD events with statin therapy increasing annually | | £2,750 | | £7,800 | | £4,320 | | £12,370 | | £7,070 | | £8,060 | | £9,090 | | £11,410 |  |
| Relative reduction in CVD events with statin therapy decreasing annually | | £6,580 | | -£44,990* | | £11,880 | | -£38,650* | | £411,880 | | -£54,160* | | -£185,710* | | -£71,380* |  |
| Relative reduction in CVD events with statin therapy reduced in elderly | | £5,170 | | £28,220 | | £8,370 | | £135,170 | | £23,810 | | £39,040 | | £42,510 | | £65,690 |  |
| LDL reduction with statin 80% from expected | | £4,450 | | £33,890 | | £7,030 | | £147,070 | | £23,110 | | £38,700 | | £33,770 | | £54,030 |  |
| Rate ratio in incident cancer with statin therapy of 0.96 | | £3,590 | | £18,360 | | £5,750 | | £47,000 | | £15,020 | | £22,730 | | £22,620 | | £33,550 |  |
| Rate ratio in incident cancer with statin therapy of 1.05 | | £3,660 | | £19,680 | | £5,850 | | £49,190 | | £16,050 | | £24,610 | | £23,560 | | £35,210 |  |
| Risk of nonvascular death increased by 20% | | £3,550 | | £20,560 | | £5,840 | | £57,920 | | £16,170 | | £25,420 | | £25,150 | | £38,900 |  |
| Compliance with statin therapy as in routine care | | £4,800 | | £33,030 | | £7,500 | | £101,540 | | £22,570 | | £31,820 | | £30,380 | | £46,700 |  |
| With quality of life disutility of daily statin pill of 0.001/year | | £3,630 | | £19,010 | | £5,810 | | £47,830 | | £15,600 | | £23,540 | | £23,090 | | £34,360 |  |
| With quality of life disutility of daily statin pill of 0.002/year | | £3,630 | | £19,060 | | £5,820 | | £48,020 | | £15,620 | | £23,590 | | £23,130 | | £34,430 |  |
| With quality of life disutility of daily statin pill of 0.003/year | | £3,640 | | £19,100 | | £5,830 | | £48,210 | | £15,650 | | £23,640 | | £23,170 | | £34,510 |  |
| Quality of life disutilities of CVD events reduced by 50% | | £3,790 | | £20,690 | | £6,150 | | £59,350 | | £16,590 | | £25,460 | | £25,960 | | £38,670 |  |
| Quality of life disutilities of CVD events increased by 50% | | £3,470 | | £17,520 | | £5,490 | | £39,790 | | £14,670 | | £21,810 | | £20,720 | | £30,800 |  |
| Quality of life disutilities of diabetes reduced by 50% | | £3,360 | | £13,760 | | £5,160 | | £25,390 | | £13,250 | | £17,670 | | £18,610 | | £25,730 |  |
| Discount rates for costs and outcomes at 1.5% per annum | | £3,710 | | £13,500 | | £5,420 | | £28,210 | | £11,280 | | £15,000 | | £15,530 | | £20,480 |  |
| Include only healthcare costs for CVD and incident diabetes | | £2,260 | | £17,040 | | £4,280 | | £42,940 | | £14,250 | | £21,960 | | £21,480 | | £32,420 |  |
| Cost of statin x1.5 | | £5,300 | | £26,240 | | £7,680 | | £63,090 | | £22,260 | | £33,390 | | £32,680 | | £48,600 |  |
| Cost of statin x2 | | £6,980 | | £33,510 | | £9,570 | | £78,530 | | £28,950 | | £43,280 | | £42,310 | | £62,920 |  |
| Cost of statin x5 | | £17,050 | | £77,140 | | £20,900 | | £171,210 | | £69,070 | | £102,630 | | £100,100 | | £148,800 |  |
| With variability around the % reduction in LDL-C with statin therapy | £3,780 | | £21,020 | | £6,000 | | £46,000 | | £16,860 | | £24,500 | | £23,380 | | £35,380 | | |

CVD, cardiovascular disease. Negative values in brackets represent cost savings. *Negative values indicate result due to decrease in QALYs.

1. **People with history of cardiovascular disease**

|  | **Standard statin vs. No statin therapy** | | | | **Higher intensity vs standard statin therapy** | | | |
| --- | --- | --- | --- | --- | --- | --- | --- | --- |
| **Sex, cardiovascular risk** | **Men, very high** | **Men, high** | **Women, very high** | **Women, high** | **Men, very high** | **Men, high** | **Women, very high** | **Women, high** |
| **Age (years)** | **60-70** | **40-49** | **60-70** | **40-49** | **60-70** | **40-49** | **60-70** | **40-49** |
| **LDL cholesterol (mmol/L)** | **≥4.1** | **<3.4** | **≥4.1** | **<3.4** | **≥4.1** | **<3.4** | **≥4.1** | **<3.4** |
| **Base-case** | £280 | £3,990 | £1,730 | £6,410 | £2,610 | £8,650 | £5,760 | £11,270 |
| Relative reduction in CVD events with statin therapy increasing annually | £1,400 | £3,420 | £2,140 | £4,140 | £2,630 | £5,470 | £4,330 | £6,090 |
| Relative reduction in CVD events with statin therapy decreasing annually | [-£70] | £6,860 | £1,980 | £12,930 | £3,140 | £30,320 | £9,380 | £46,110 |
| Relative reduction in CVD events with statin therapy reduced in elderly | £790 | £4,020 | £2,080 | £6,980 | £3,790 | £9,710 | £7,440 | £13,710 |
| LDL reduction with statin 80% from expected | £660 | £5,030 | £2,290 | £7,830 | £3,220 | £11,370 | £7,240 | £15,050 |
| Rate ratio in incident cancer with statin therapy of 0.96 | £270 | £3,270 | £1,580 | £5,000 | £2,640 | £8,610 | £5,760 | £11,170 |
| Rate ratio in incident cancer with statin therapy of 1.05 | £290 | £5,260 | £2,000 | £9,320 | £2,570 | £8,730 | £5,770 | £11,430 |
| Risk of nonvascular death increased by 20% | [-£100] | £3,950 | £1,530 | £6,540 | £2,170 | £8,840 | £5,570 | £11,790 |
| Compliance with statin therapy as in routine care | £900 | £4,980 | £2,450 | £7,710 | £4,240 | £10,830 | £8,250 | £14,500 |
| With quality of life disutility of daily statin pill of 0.001/year | £300 | £4,640 | £1,900 | £7,950 | £2,610 | £8,670 | £5,780 | £11,290 |
| With quality of life disutility of daily statin pill of 0.002/year | £320 | £5,540 | £2,090 | £10,470 | £2,620 | £8,690 | £5,790 | £11,310 |
| With quality of life disutility of daily statin pill of 0.003/year | £340 | £6,880 | £2,340 | £15,310 | £2,630 | £8,700 | £5,810 | £11,330 |
| Quality of life disutilities of CVD events reduced by 50% | £270 | £4,010 | £1,770 | £6,720 | £2,550 | £8,760 | £6,040 | £12,050 |
| Quality of life disutilities of CVD events increased by 50% | £290 | £3,970 | £1,700 | £6,130 | £2,670 | £8,550 | £5,520 | £10,580 |
| Quality of life disutilities of diabetes reduced by 50% | £270 | £3,850 | £1,670 | £6,250 | £2,340 | £7,450 | £4,950 | £9,940 |
| Discount rates for costs and outcomes at 1.5% per annum | £1,480 | £4,080 | £2,680 | £5,640 | £3,800 | £7,990 | £6,410 | £9,380 |
| Include only healthcare costs for CVD and incident diabetes | [-£2,940] | £1,550 | [-£1,700] | £3,950 | [-£1,190] | £5,610 | £1,530 | £8,420 |
| Cost of statin x1.5 | £710 | £4,990 | £2,350 | £7,800 | £3,900 | £11,660 | £7,790 | £15,320 |
| Cost of statin x2 | £1,140 | £6,000 | £2,960 | £9,190 | £5,190 | £14,670 | £9,810 | £19,380 |
| Cost of statin x5 | £3,730 | £12,020 | £6,660 | £17,530 | £12,950 | £32,720 | £21,950 | £43,720 |
| With variability around the % reduction in LDL-C with statin therapy | £360 | £4,030 | £1,790 | £6,450 | £2,710 | £8,820 | £6,040 | £11,590 |

LDL, low density lipoprotein; CVD, cardiovascular disease. Negative values in brackets represent cost savings.

## Supplementary Figure S7 Scenario analysis of QALYs gained and cost-effectiveness with long-term standard statin therapy in categories by sex, age, pre-treatment LDL cholesterol level and 10-year cardiovascular risk with added hypothetical disutility of daily pill of 0.002 QALYs/year

QALY, quality-adjusted life year; LDL, low density lipoprotein; CVD, cardiovascular disease; ICER, incremental cost-effectiveness ratio with costs and QALYs discounted at 3.5% per year. For the category of women 40-49 years old with 10-year cardiovascular risk<5% and LDL cholesterol<3.4mmol/L, small discounted QALYs are projected as gains in QALYs accrue later in life.

## Supplementary Table S6 A scenario analysis of stopping statin treatment at 80 years of age

1. **People without history of cardiovascular disease**

| **Sex, cardiovascular risk** | | **Men, high** | **Men, moderate** | **Women, high** | **Women, moderate** |
| --- | --- | --- | --- | --- | --- |
| **Age (years)** | | **60-70** | **40-49** | **60-70** | **40-49** |
| **10-year cardiovascular risk (%)** | | **15-20** | **5-10** | **15-20** | **5-10** |
| **LDL cholesterol (mmol/L)** | | **≥4.1** | **<3.4** | **≥4.1** | **<3.4** |
| **Standard statin therapy** | | | | | |
| Standard statin until 80 years of age vs No statin | Undiscounted QALYs gained | 0.21 | 0.19 | 0.16 | 0.13 |
|  | £/QALY^1^ | [£485] | £3,499 | £849 | £7,637 |
| Lifetime Standard statin vs Standard statin until 80 years of age | Undiscounted QALYs gained | 0.39 | 0.13 | 0.45 | 0.12 |
|  | £/QALY^1^ | £1,893 | £3,215 | £1,808 | £4,617 |
| **Higher intensity statin therapy** | | | | | |
| Higher intensity statin until 80 years of age vs No statin | Undiscounted QALYs gained | 0.23 | 0.20 | 0.16 | 0.13 |
|  | £/QALY^1^ | £215 | £5,814 | £2,400 | £12,641 |
| Lifetime higher intensity statin vs Higher intensity statin until 80 years of age | Undiscounted QALYs gained | 0.47 | 0.16 | 0.55 | 0.15 |
|  | £/QALY^1^ | £1,975 | £3,374 | £1,952 | £4,905 |

1. **People with CVD**

| **Sex, cardiovascular risk** | | **Men, very high** | **Men, high** | **Women, very high** | **Women, high** |
| --- | --- | --- | --- | --- | --- |
| **Age (years)** | | **60-70** | **40-49** | **60-70** | **40-49** |
| **LDL cholesterol (mmol/L)** | | **≥4.1** | **<3.4** | **≥4.1** | **<3.4** |
| **Standard statin** | | | | | |
| Standard statin until 80 years of age vs No statin | Undiscounted QALYs gained | 0.25 | 0.30 | 0.18 | 0.22 |
|  | £/QALY^1^ | [£1220] | £3,810 | £230 | £6,810 |
| Lifetime Standard statin vs Standard statin until 80 years of age | Undiscounted QALYs gained | 0.20 | 0.08 | 0.27 | 0.13 |
|  | £/QALY^1^ | £3,020 | £5,160 | £3,290 | £5,230 |
| **Higher intensity statin** | | | | | |
| Higher intensity statin until 80 years of age vs No statin | Undiscounted QALYs gained | 0.29 | 0.36 | 0.20 | 0.26 |
|  | £/QALY^1^ | [£780] | £4,600 | £1,140 | £7,830 |
| Lifetime higher intensity statin vs Higher intensity statin until 80 years of age | Undiscounted QALYs gained | 0.25 | 0.10 | 0.33 | 0.16 |
|  | £/QALY^1^ | £3,050 | £5,210 | £3,370 | £5,320 |

^1^Costs and outcomes discounted at 3.5%/year.

Values in brackets represent cost savings. LDL, low density lipoprotein; QALY, quality-adjusted life year.

## Supplementary Table S7 A scenario analysis of delaying statin treatment in 40 to 44 years old people without history of cardiovascular disease

|  |  | **Men 40-44 years old,**  **by 10-year CVD risk (%)** | | | **Women 40-44 years old,**  **by 10-year CVD risk (%)** | | |
| --- | --- | --- | --- | --- | --- | --- | --- |
|  |  | **<5** | **5-10** | **≥10** | **<5** | **5-10** | **≥10** |
| Immediate initiation vs Delayed by 5 years lifetime standard statin | Undiscounted QALYs gained | 0.01 | 0.02 | 0.05 | 0.00 | 0.01 | 0.03 |
|  | £/QALY^1^ | £10,384 | £239 | [£2,582] | £33,948 | £5,907 | £1,624 |
| Immediate initiation vs Delayed by 5 years lifetime higher intensity statin therapy | Undiscounted QALYs gained | 0.01 | 0.02 | 0.06 | 0.01 | 0.01 | 0.03 |
|  | £/QALY^1^ | £14,536 | £2,043 | [£1,979] | £41,888 | £9,279 | £3,054 |

^1^Wth costs and outcomes discounted at 3.5%/year.

Values in brackets represent cost savings. CVD, cardiovascular disease; LDL, low density lipoprotein; QALY, quality-adjusted life year.

## Supplementary references

1. Wu R, Williams C, Zhou J, et al. Long-term cardiovascular risks and statin treatment impact on socioeconomic inequalities: microsimulation model. *Brit J Gen Pract*. 2023:BJGP.2023.0198.

2. Li Y, Sperrin M, van Staa T. R package “QRISK3”: an unofficial research purposed implementation of ClinRisk’s QRISK3 algorithm into R. *F1000Research* 2020;8(2139).

3. UK Biobank. Guidelines for Data Processing and Analysis of the International Physical Activity Questionnaire (IPAQ) 2005 [cited 24/08/2022]. Available from: <https://biobank.ndph.ox.ac.uk/showcase/ukb/docs/ipaq_analysis.pdf>.

4. Lourida I, Hannon E, Littlejohns TJ, et al. Association of Lifestyle and Genetic Risk With Incidence of Dementia. *JAMA*. 2019;322(5):430-37.

5. Hippisley-Cox J, Coupland C, Brindle P. Development and validation of QRISK3 risk prediction algorithms to estimate future risk of cardiovascular disease: prospective cohort study. *BMJ*. 2017;357:j2099.

6. Carter AR, Gill D, Davey Smith G, Taylor AE, Davies NM, Howe LD. Cross-sectional analysis of educational inequalities in primary prevention statin use in UK Biobank. *Heart*. 2022;108(7):536-42.

7. Downs J, Gotto A, Clearfield M, et al. Protocol for a prospective collaborative overview of all current and planned randomized trials of cholesterol treatment regimens. *Am J Cardiol*. 1995;75(16).

8. Sudlow C, Gallacher J, Allen N, et al. UK Biobank: an open access resource for identifying the causes of a wide range of complex diseases of middle and old age. *PLoS Med*. 2015;12(3):e1001779.

9. Zhou J, Wu R, Williams C, et al. Prediction Models for Individual-Level Healthcare Costs Associated with Cardiovascular Events in the UK. *Pharmacoeconomics*. 2023;41(5):547-59.

10. Cholesterol Treatment Trialists' Collaboration. Efficacy and safety of statin therapy in older people: a meta-analysis of individual participant data from 28 randomised controlled trials. *Lancet*. 2019;393(10170):407-15.

11. Sattar N, Preiss D, Murray HM, et al. Statins and risk of incident diabetes: a collaborative meta-analysis of randomised statin trials. *Lancet*. 2010;375(9716):735-42.

12. Preiss D, Seshasai SR, Welsh P, et al. Risk of incident diabetes with intensive-dose compared with moderate-dose statin therapy: a meta-analysis. *JAMA*. 2011;305(24):2556-64.

13. Law MR, Wald NJ, Rudnicka AR. Quantifying effect of statins on low density lipoprotein cholesterol, ischaemic heart disease, and stroke: systematic review and meta-analysis. *BMJ*. 2003;326(7404):1423.

14. Ference BA, Ginsberg HN, Graham I, et al. Low-density lipoproteins cause atherosclerotic cardiovascular disease. 1. Evidence from genetic, epidemiologic, and clinical studies. A consensus statement from the European Atherosclerosis Society Consensus Panel. *Eur Heart J*. 2017;38(32):2459-72.

15. Cholesterol Treatment Trialists' (CTT) Collaboration. Lack of effect of lowering LDL cholesterol on cancer: meta-analysis of individual data from 175,000 people in 27 randomised trials of statin therapy. *PLoS One*. 2012;7(1):e29849.

16. Pate A, Elliott RA, Gkountouras G, Thompson A, Emsley R, van Staa T. The impact of statin discontinuation and restarting rates on the optimal time to initiate statins and on the number of cardiovascular events prevented. *Pharmacoepidemiol Drug Saf*. 2020;29(6):644-52.

17. Hutchins R, Viera AJ, Sheridan SL, Pignone MP. Quantifying the utility of taking pills for cardiovascular prevention. *Circ Cardiovasc Qual Outcomes*. 2015;8(2):155-63.

18. National Institute for Health and Care Excellence. NICE health technology evaluations: the manual. 2022 [cited 19/03/2022]; (PMG36). Available from: <https://www.nice.org.uk/process/pmg36>.

19. Karlson BW, Wiklund O, Palmer MK, Nicholls SJ, Lundman P, Barter PJ. Variability of low-density lipoprotein cholesterol response with different doses of atorvastatin, rosuvastatin, and simvastatin: results from VOYAGER. *Eur Heart J Cardiovasc Pharmacother*. 2016;2(4):212-7.
